# Specialist Referral for Cardiovascular Risk in Patients With Prostate Cancer: A Randomized Clinical Trial

**DOI:** 10.1001/jamainternmed.2026.4773

**Published:** 2026-08-30

**Authors:** Darryl P. Leong, Celestia Higano, Cristina Cano Garcia, Vincent Fradet, D. Robert Siemens, Tamim Niazi, Bobby Shayegan, Avirup Guha, Filipe Cirne, Joseph B. Selvanayagam, Philippe D. Violette, Felipe H. Valle, Luke T. Lavallée, Emmanuelle Duceppe, Patrick Anderson, Denis Xavier, Jose Patricio Lopez-Lopez, Nicolas Villareal Trujillo, Ariel Galapo Kann, Rafaela K. Piccoli, Marina Mourtzakis, David Sarid, Srivatsa Narasimha, Patrick P.W. Luke, Ludhmila A. Hajjar, Himu Lukka, Laurence Klotz, Kumar Balasubramanian, Rajibul Mian, Steven Agapay, Sumathy Rangarajan, Sarah Karampatos, Kelvin K. H. Ng, Alvaro Avezum, P. J. Devereaux, Jehonathan H. Pinthus

**Affiliations:** 1 Population Health Research Institute, McMaster University and Hamilton Health Sciences, Hamilton, Ontario, Canada; 2 Department of Medicine, McMaster University, Hamilton, Ontario, Canada; 3 Department of Urologic Sciences, University of British Columbia and Vancouver Prostate Centre, Vancouver, Ontario, Canada; 4 Department of Surgery, McMaster University, Hamilton, Ontario, Canada; 5 Department of Urology, Goethe University Frankfurt am Main, Frankfurt, Germany; 6 Département de Chirurgie, Université Laval, Quebec City, Quebec, Canada; 7 Department of Urology, Queens University, Kingston, Ontario, Canada; 8 Department of Oncology, McGill University, Montreal, Quebec, Canada; 9 Division of Cardiovascular Disease, Augusta University, Georgia, Augusta; 10 Flinders University and Flinders Medical Centre, Bedford Park, South Australia, Australia; 11 Woodstock General Hospital, Woodstock, Ontario, Canada; 12 Division of Cardiology, Hospital de Clínicas de Porto Alegre, Porto Alegre, Brazil; 13 Division of Urology, University of Ottawa, Ottawa, Ontario, Canada; 14 Department of Medicine, Centre Hospitalier de l’Université de Montreal, Montreal, Quebec, Canada; 15 Niagara Health, St Catharine’s, Canada and Department of Surgery, McMaster University, Hamilton, Ontario, Canada; 16 St John’s Medical College and Research Institute, Bangalore, India; 17 Masira Research Institute, Universidad de Santander and Centro del Cancer, Clinica Foscal Internacional, Bucaramanga, Colombia; 18 FOSCAL Internacional and Universidad Autónoma de Bucaramanga, Bucaramanga, Colombia; 19 Hospital Alemao Oswaldo Cruz, São Paulo, Brazil; 20 Department of Oncology, Universidade Regional do Noroeste do Estado do Rio Grande do Sul, Ijuí, Brazil; 21 Department of Kinesiology and Health Sciences, University of Waterloo, Waterloo, Ontario, Canada; 22 Tel Aviv Sourasky Medical Centre, Tel Aviv, Israel; 23 Department of Urologic Oncology, Sri Shankara Cancer Hospital and Research Centre, Bangalore, India; 24 Department of Surgery, London Health Science Centre, London, Ontario, Canada; 25 Faculdade de Medicina da Universidade de São Paolo, São Paolo, Brazil; 26 Department of Oncology, McMaster University, Hamilton, Ontario, Canada; 27 Sunnybrook Health Sciences Centre and Department of Surgery, University of Toronto, Toronto, Ontario, Canada; 28 International Research Center & Cardiology Department, Hospital Alemão Oswaldo Cruz, São Paulo, Brazil; 29 Division of Perioperative Medicine, McMaster University, Hamilton, Ontario, Canada; 30 McMaster University, Hamilton, Ontario, Canada

## Abstract

**Importance:**

Patients with prostate cancer have a high burden of cardiovascular risk factors, often suboptimally controlled, and adverse cardiovascular outcomes.

**Objective:**

To determine whether the routine referral of patients with prostate cancer to a cardiovascular specialist to implement a systematic risk factor strategy is more likely to reduce adverse cardiovascular outcomes and improve risk factor control than usual care.

**Design, Settings, and Participants:**

This randomized clinical trial included patients with prostate cancer from 55 sites in 8 countries between 2015 and 2025. Eligible patients were diagnosed with prostate cancer during the past 12 months; had received treatment with androgen deprivation therapy (ADT) for the first time within the past 6 months; or planned to start ADT in the next month. Patients taking a statin with a systolic blood pressure of 130 mm Hg or lower were ineligible. Data were analyzed from May 25 to August 7, 2026.

**Intervention:**

Patients were allocated in a 1:1 ratio to receive usual care alone or usual care plus routine referral to an internist or cardiologist. The specialists provided a systematic intervention, including a target of systolic blood pressure of 130 mm Hg or lower and a statin medication, irrespective of the patient’s cholesterol levels (even if not usual or guideline-driven practice); encourage smoking cessation; and provide guidance on diet and exercise.

**Main Outcomes and Measures:**

Hierarchical composite of cardiovascular death, myocardial infarction, stroke, heart failure, suboptimal cholesterol (total cholesterol, >155 mg/dL [to convert to mmol/L, multiply by 0.0259]) and suboptimal blood pressure (systolic blood pressure, >130 mm Hg) as evaluated by the win ratio.

**Results:**

The analysis included 2487 patients with prostate cancer (mean [SD] age, 68 [8] years). During median (IQR) follow-up of 5.8 (2.7-8.2) years, the win ratio in favor of the intervention was 1.60 (95% CI, 1.42-1.81), mostly attributable to lower cholesterol in the intervention group (mean difference, 12 mg/dL; 95% CI, 9-15 mg/dL) as a consequence of greater protocol-mandated statin use. Mean (SD) close-out systolic blood pressure values were 131.1 (16.9) mm Hg in the intervention group and 132.9 (18.3) mm Hg in the control group. There was no difference in time to cardiovascular death, myocardial infarction, stroke, or heart failure between groups (subdistribution hazard ratio, 1.08; 95% CI, 0.79-1.49).

**Conclusions and Relevance:**

In this randomized clinical trial, routine referral of patients with prostate cancer to a cardiovascular specialist lead to improved outcomes, specifically through better cholesterol control. However, it is uncertain whether this reduced clinical cardiovascular events.

**Trial Registration:**

ClinicalTrials.gov Identifier: NCT03127631

## Key Points

**Question:**

Should all patients with prostate cancer be referred to a cardiovascular specialist?

**Findings:**

In this randomized trial of 2487 patients with prostate cancer, those referred were more likely to experience a favorable outcome. This was due to better cholesterol control rather than a reduced risk of clinical cardiovascular events.

**Meaning:**

These findings indicate that routine referral of patients with prostate cancer to a cardiovascular specialist leads to improved risk factor control; however, the effect of improved cardiovascular risk factor treatment on long-term cardiovascular outcomes in this population remains uncertain.

## Teaser

This randomized clinical trial evaluates the effect of routine referral of patients with prostate cancer to a cardiovascular specialist for systematic risk factor control compared with usual care.

## Introduction

Cardiovascular disease is a leading cause of morbidity and mortality among individuals with prostate cancer. In US population-based analyses, cardiovascular disease is the most frequent cause of death among those with nonmetastatic prostate cancer, accounting for 23% of deaths (in contrast to 17% of deaths from prostate cancer in this population).^1^ Even among those with metastatic disease, cardiovascular mortality rates exceed those observed in the general population, with a standardized mortality ratio of 1.48 (95% CI, 1.41-1.54). Data from the Swedish national health care registry^2^ and Kaiser Permanente Southern California^3^ demonstrate incidence rates of ischemic heart disease, stroke, and heart failure exceeding 20 per 1000 person-years among patients with prostate cancer, consistent with high risk, and the risk appears to be even higher after initiating androgen deprivation therapy (ADT).^2^

Several factors likely contribute to this elevated cardiovascular risk. Patients with prostate cancer frequently have prevalent cardiovascular risk factors and established cardiovascular disease,^5^ and these risk factors are often inadequately controlled.^6^ In addition, ADT, which is used in many patients with prostate cancer,^8^ has been associated with adverse metabolic effects, including an increased risk of diabetes and hypertension^9^ as well as cardiovascular disease.^4^

A diagnosis of cancer is often considered a teachable moment.^12^ This patient population is one already engaged in the health care system and undergoing common clinical examinations and investigations. Recognizing these risks and opportunities, professional organizations recommend baseline and annual routine cardiovascular risk assessment and management in patients with prostate cancer, particularly among those receiving ADT. Recommended strategies include blood pressure control, lipid-lowering therapy, smoking cessation, dietary modification and exercise^13^ and cardiologist referral for high-risk individuals.^15^ Despite these recommendations, direct randomized evidence supporting cardiovascular strategies in this population is lacking.^17^

To address this gap, we conducted a pragmatic randomized clinical trial to determine whether the routine referral of patients with prostate cancer to an internist or cardiologist, who implemented a systematic cardiovascular risk reduction strategy (the Randomized Intervention for Cardiovascular and Lifestyle Risk Factors in Prostate Cancer Patients [RADICAL PC2]), would improve risk factor control and reduce adverse cardiovascular outcomes compared with usual care.

## Methods

This trial was reviewed and approved by the institutional review boards at all participating centers. Written informed consent was obtained from all participants and all data were deidentified. We followed the Consolidated Standards of Reporting Trials (CONSORT) reporting guideline.^18^ The most recent revision of the trial protocol, version 6, is available in Supplement 1 and the statistical analysis plan, in Supplement 2. The trial methods have been previously reported.^19^

### Participants and Settings

Patients were screened for eligibility in urology, medical oncology, and radiation oncology clinics in 55 sites in academic and community settings in 8 countries (Australia, Brazil, Canada, Colombia, India, Israel, Singapore, and United States). Inclusion criteria required patients to have a physician’s diagnosis of prostate cancer and to meet one of the following: first diagnosis of prostate cancer within the previous 12 months; first treatment with ADT for prostate cancer within the previous 6 months; or a plan to commence ADT for prostate cancer within 1 month. Patients were ineligible if they were younger than 45 years or already consulting a cardiologist annually or taking a statin and having a systolic blood pressure of 130 mm Hg or lower. Demographic characteristics, including race and ethnicity, were collected by self-report; other included Arab, Chinese, Hispanic, indigenous, Persian, South Asian, and Southeast Asian.

### Randomization

**Figure 1.**
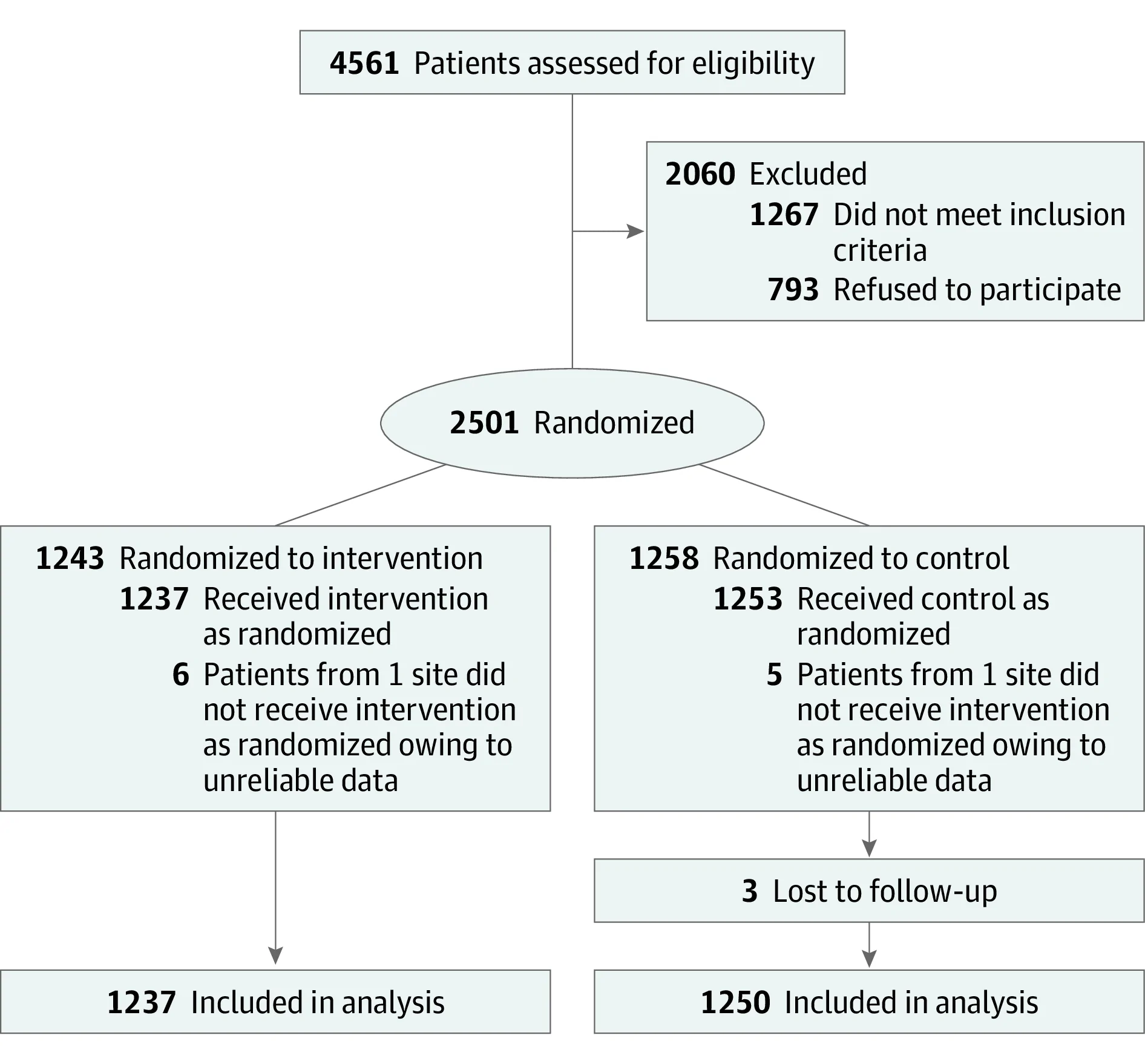
Flow Diagram of Study Participants

Study personnel randomized eligible patients using a web-based, computer-generated randomization schedule, in a 1:1 ratio, to the intervention or the control group. Randomization was stratified by center and ADT use (none, <6, 6-24, and >24 months), and permuted block sizes of 2, 4, and 6 were used to ensure concealment of randomization. We randomized 2501 participants between October 21, 2015, and July 2, 2025 (Figure 1). Of these, 3 (0.1%) withdrew consent during follow-up. One site was disqualified due to data reliability concerns; their 11 participants were not included in the analysis.

### Study Groups and Intervention

The intervention group was referred to an internist or cardiologist who was requested to offer the following: (1) standardized advice on healthy diet (Diet Intervention Handout^20^) and exercise (A Home-Based Exercise Program^21^); (2) advice to quit smoking, and information on available aids according to local practice; (3) statin prescription (irrespective of cholesterol levels); and (4), for baseline systolic blood pressure greater than 130 mm Hg, prescription for a blood pressure−lowering medication.

Initially the trial required an antiplatelet to be prescribed to all participants in the intervention group. Following the publication of data suggesting that low-dose aspirin for primary prevention of cardiovascular disease may have a lower likelihood of net clinical benefit than previously anticipated,^22^ the protocol was amended to leave the prescription of an antiplatelet to the discretion of the treating internist or cardiologist.

Individuals in the control group received usual care according to local standards and could be referred to an internist or cardiologist at the discretion of their routine health care professionals, if determined to be clinically indicated. Patients, data collectors, and health care professionals were unblinded to treatment allocation. However, outcome adjudicators were blinded to treatment allocation.

### Follow-Up Period

Participants were followed up at 3-, 6-, 12-, 18-, and 24-month visits, then annually until study close-out. Owing to restrictions of face-to-face visits during the COVID-19 pandemic, the protocol was amended to permit data collection by telephone, including the recording of vital signs using a home blood pressure monitor. Annual blood testing for cholesterol concentration levels was considered standard of care in this patient population; investigators were asked to order the test annually. The 36-month visit was by telephone for all participants.

### Primary and Safety Outcomes

The powered first primary effectiveness outcome was the hierarchical composite of cardiovascular death, myocardial infarction (MI), stroke, heart failure, suboptimal cholesterol control (defined as total cholesterol >155 mg/dL [to convert to mmol/L, multiply by 0.0259]) and suboptimal systolic blood pressure control, defined as >130 mm Hg. The nonpowered second primary effectiveness outcome was the time to the first occurrence of the composite of cardiovascular death, MI, stroke, and heart failure (ie, this outcome was prespecified as a primary outcome but the study design, including sample size and follow-up duration, was not predicated on observing a defined number of these outcome events).

Safety outcomes were major bleeding, myositis, liver injury, and kidney injury. Clinical outcomes were adjudicated according to standardized definitions (available in the trial protocol in Supplement 1) by an independent events adjudication committee. An independent data monitoring committee reviewed unblinded adverse event and outcome data on a periodic basis. However, no formal interim analyses to assess for efficacy were performed.

Trial coordination—including maintenance of the web randomization system, database, and statistical analyses—was conducted at the Population Health Research Institute, McMaster University, and Hamilton Health Sciences (Hamilton, Canada). The trial coordination team did not have access to participant randomization allocation or participant health records.

### Statistical Analysis

A sample size of 2500 participants was determined to have 86% power to detect a win ratio of 1.22 or greater in the intervention group, with 34% of control-intervention pairs tied. A win ratio of 1.22 would indicate that if a pair of individuals were chosen at random—1 from the intervention group and 1 from the control group—and were not tied (ie, they differed with respect to a cardiovascular event, cholesterol, or blood pressure control), the individual in the intervention group would be 22% more likely to be the winner. As a hierarchical metric, pairs of participants are first evaluated for differences in clinical cardiovascular events, then for differences in cholesterol levels if tied for clinical cardiovascular events, and finally for differences in systolic blood pressure if also tied for cholesterol levels. No imputation of missing data was performed.

Analysis of the first primary effectiveness outcome was based on the intention-to-treat principle and was conducted using the win ratio approach. The win ratio was calculated as the number of intervention-control pairs in which the intervention is the winner divided by the number of intervention-control pairs in which the intervention is the loser. The individual components of the win ratio were, in hierarchical order: time to cardiovascular death; time to first MI; time to first stroke; time to first heart failure event; total cholesterol level greater than 155 mg/dL; and systolic blood pressure greater than 130 mm Hg. Cholesterol concentration and blood pressure measurements were taken from the most recent follow-up visit (ie, if these values were unavailable at the close-out visit, the next most recent values were used in the analysis).

For the second primary effectiveness outcome (time to the first occurrence of the composite of cardiovascular death, MI, stroke, and heart failure), cumulative incidence curves were constructed and the subdistribution hazard ratio for the intervention, along with 95% CIs, was estimated using competing risks regression. The occurrence of noncardiovascular death in the absence of a primary effectiveness outcome was considered a competing risk.

The first primary effectiveness outcome was tested with a type I error rate of 5%. For the second primary effectiveness outcome, to ensure that the overall type I error rate at the final analysis was maintained, a gatekeeping hierarchical hypothesis testing strategy was used: the second primary effectiveness outcome was only tested (at 5% α threshold) if the test of the first primary effectiveness outcome met a *P* < .05 threshold. Exploratory analyses were performed for each component of the first primary effectiveness outcome. Statistical tests were 2-tailed. Data were analyzed from May 25 to August 7, 2026, using SAS (version 9.4, SAS Institute).

## Results

The analysis included 2487 participants (mean [SD] age, 68 [8] years]). Complete baseline characteristics are presented in Table 1. Intervention and control groups were well balanced. At baseline, 337 participants (14%) had metastatic disease, 669 (27%) had undergone prostatectomy, 687 (28%) prostate radiotherapy, and 249 (10%) were under observational strategies for prostate cancer, while 1128 (45%) had been or were receiving ADT.

**Table 1.** Baseline Characteristics of Study Participants

Characteristic	No. (%)		Standardized mean/proportion difference
	Intervention (n = 1237)	Control (n = 1250)	
**Demographic**			
Age, mean (SD), y	68 (8)	68 (8)	−0.0065
Race and ethnicity^a^			
Black	97 (8)	79 (6)	0.0593
Latino	64 (5)	79 (6)	−0.0493
White	1028 (83)	1034 (83)	0.0102
Other	45 (4)	53 (4)	−0.0310
Education level			
<High school	274 (22)	288 (23)	−0.0213
High school diploma	331 (27)	349 (28)	−0.0261
>High school	618 (50)	601 (48)	0.0376
Employed (yes)	480 (39)	456 (36)	0.048
**Cancer-related risks**			
Tobacco			
Never	549 (44)	571 (46)	−0.0261
Former	565 (46)	530 (42)	0.066
Current	119 (10)	146 (12)	−0.0668
NCCN risk (nonmetastatic prostate cancer)			
Low	82 (7)	104 (8)	−0.0643
Intermediate	500 (41)	455 (37)	0.0827
High	499 (40)	504 (40)	0.0004
Metastases	153 (12)	184 (15)	−0.0688
**Treatment**			
Prostatectomy	343 (28)	326 (26)	0.0372
Prostate radiotherapy	349 (28)	338 (27)	0.0262
Observation	111 (9)	138 (11)	−0.0689
Chemotherapy	39 (3)	33 (3)	0.0306
ADT			
GnRH agonist	332 (27)	345 (28)	−0.0171
GnRH antagonist	49 (4)	54 (4)	−0.018
**Cardiovascular conditions and comorbidities**			
Myocardial infarction	39 (3)	29 (2)	0.0511
CAD	57(5)	56(4)	0.0061
Stroke	25 (2)	33 (3)	−0.041
Peripheral arterial disease	15 (1)	17 (1)	−0.0131
Heart failure	15 (1)	16 (1)	−0.0061
Atrial fibrillation	35 (3)	31 (2)	0.0217
Hypertension	536 (43)	501 (40)	0.066
Diabetes	187 (15)	172 (14)	0.0386
**Medications**			
Antiplatelet	203 (16)	196 (16)	0.0199
Anticoagulant	42 (3)	31 (2)	0.0542
Statin therapy	372 (30)	365 (29)	0.0191
Antihypertensive medication	573 (46)	553 (44)	0.0418
**Clinical measures**			
BMI score, mean (SD)	28 (5)	28 (4)	0.0976
Systolic blood pressure, mm Hg	138 (19)	139 (18)	−0.0421
Diastolic blood pressure, mm Hg	83 (11)	83 (11)	−0.0349
Cholesterol concentration, mg/dL^b^			
Total cholesterol	185.6 (42.5)	189.5 (42.5)	−0.0952
LDL	104.4 (38.7)	108.3 (38.7)	−0.0765
HDL	50.3 (15.5)	54.1 (15.5)	−0.1435
Triglycerides^c^	150.6 (88.6)	150.6 (97.4)	−0.0036
HbA_1c_, %	5.7 (1.2)	5.7 (1.2)	0.0275
eGFR, mL/min/1.73m^2^	80 (23)	81 (34)	−0.0215

Abbreviations: ADT, androgen deprivation therapy; BMI, body mass index (calculated as weight in kilograms divided by height in meters squared); CAD, coronary artery disease including myocardial infarction, percutaneous coronary intervention, coronary artery bypass surgery, and angina; eGFR, estimated glomerular filtrate rate; GnRH, gonadotropin-releasing hormone; HbA_1c_, glycated hemoglobin; HDL, high-density lipoprotein; LDL, low-density lipoprotein; NCCN, US National Comprehensive Cancer Network.

^a^ Race and ethnicity data were collected self-reported; other included Arab, Chinese, indigenous, Persian, South Asian, and Southeast Asian.

^b^ To convert to mmol/L, multiply by 0.0259.

^c^ To convert to mmol/L, multiply by 0.0113.

Tobacco was never used by 1120 participants (45%), formerly used by 1095 (44%), and currently used by 265 (11%). Diabetes was reported in 359 participants (14%) and hypertension in 1037 (42%). There was a history of coronary artery disease, including MI, angina, percutaneous coronary intervention, or coronary artery bypass surgery in 113 participants (5%), stroke in 58 (2%), peripheral arterial disease in 32 (1%), heart failure in 31 (1%), and atrial fibrillation in 66 (3%). Mean (SD) body mass index (BMI; calculated as weight in kilograms divided by height in meters squared) was 28.2 (4.5). Mean (SD) blood pressure was 139 (18) mm Hg; concentration of cholesterol, 185.6 (42.5) mg/dL; low-density lipoprotein (LDL) cholesterol, 108.3 (38.7) mg/dL; high-density lipoprotein (HDL) cholesterol, 50.3 (15.5) mg/dL; and triglycerides, 150.6 (88.6) mg/dL (to convert to mmol/L, multiply by 0.0113). A median (IQR) follow-up of 5.8 (2.7-8.2) years was completed for 2446 participants (98%).

### Adoption of Cardiovascular Interventions

During the follow-up period, in the control group, 265 participants (21%) visited an internist or cardiologist who addressed cardiovascular risk at the request of the patient’s routine physician, with a median (IQR) of 3 (1-6) visits per participant. In the intervention group, 1119 participants (90%) visited an internist or cardiologist, according to the trial protocol, with a median (IQR) of 4 (3-6) visits per participant.

At baseline, 737 participants (30%) were taking a statin medication. At study close-out, 774 participants in the intervention group (63%) and 505 in the control group (40%) were taking a statin (*P* < .001). At baseline, 1126 participants (45%) were taking a blood pressure−lowering medication. At close-out, more individuals in the intervention group (688 [56%]) were taking a blood pressure−lowering medication than the control group (607 [49%]; *P* < .001). At baseline, 399 participants (16%) were taking an antiplatelet and 73 (3%), an anticoagulant. At study close-out, 239 in the intervention group (19%) and 176 in the control group (14%) were taking an antiplatelet (*P* < .001), while 85 in the intervention group (7%) and 86 in the control group (7%) were taking an anticoagulant (*P* = .92).

Overall, the intervention increased the implementation of the cardiovascular risk-reduction strategy. Compared with participants receiving usual care, those assigned to a cardiologist or internist were substantially more likely to receive statin therapy and modestly more likely to receive blood pressure−lowering medication during follow-up.

### Primary Effectiveness Outcomes

During a median (range) follow-up of 5.8 (1.1-9.5) years, major cardiovascular events were relatively uncommon. There were 73 cardiovascular deaths (2.9%), 55 first MI (2.2%), 47 first strokes (1.9%), and 30 first heart failure (1.2%) events. Overall, 171 individuals (6.9%) experienced at least 1 component of the composite cardiovascular outcome. Across both treatment groups, mean (SD) cholesterol levels decreased from 185.6 (42.5) mg/dL) to 166.3 (42.5) mg/dL, and systolic blood pressure decreased from 139 (18) mm Hg to 132 (18) mm Hg during follow-up.

For the first primary effectiveness outcome, there were 1 147 016 pairwise comparisons, among which 257 190 (22%) were ties; the intervention was the winner in 547 745 (48%) pairs; and the control was the winner in 342 081 (30%) pairs. The win ratio in favor of the intervention was 1.60 (95% CI, 1.42-1.81; *P* < .001). The contributions of the different components of the hierarchical primary effectiveness outcome are shown in Table 2. The favorable win ratio reflected superior control of cardiovascular risk factors in the intervention group, particularly cholesterol control, rather than differences in cardiovascular event rates. Although numerical trends favored the intervention for MI and stroke, no consistent pattern of benefit was observed across individual cardiovascular event components. Net wins favored the control group for cardiovascular death and heart failure.

**Table 2.** Intervention and Control Groups: Wins, Win Ratios, and Contributions to Net Wins, Stratified by Individual Outcomes

Hierarchical component	Ties, No.	No. (%)		Component-specific win ratio	Net wins in intervention group (A)−(B)	Contribution to net wins^a^ = (A)−(B)/205 664, %
		(A) Intervention wins	(B) Control wins			
Overall	257 190	547 745	342 081	1.60	205 664	NA
Cardiovascular death	1 112 052	15 019 (1.3)	19 945 (1.7)	0.75	−4926	−2.4
Myocardial infarction	1 075 919	23 230 (2.1)	12 903 (1.2)	1.80	10 327	5.0
Stroke	1 056 475	10 054 (0.9)	9390 (0.9)	1.07	664	0.3
Heart failure	1 045 847	3850 (0.4)	6778 (0.6)	0.57	−2928	−1.4
Cholesterol >155 mg/dL^b^	512 124	369 060 (35.3)	164 663 (15.7)	2.24	204 397	99.4
Systolic blood pressure >130 mm Hg	257 190	126 532 (24.7)	128 402 (25.1)	0.99	−1870	−0.9

Abbreviation: NA, not applicable.

^a^ Contribution to net wins = (stratum-specific number of net wins in intervention group)/(overall number of net wins in intervention group (ie, 205 664). A positive value for the number of net wins and contribution to net wins indicates more wins in the intervention group than the control group in that stratum.

^b^ To convert to mmol/L, multiply by 0.0259.

**Figure 2.**
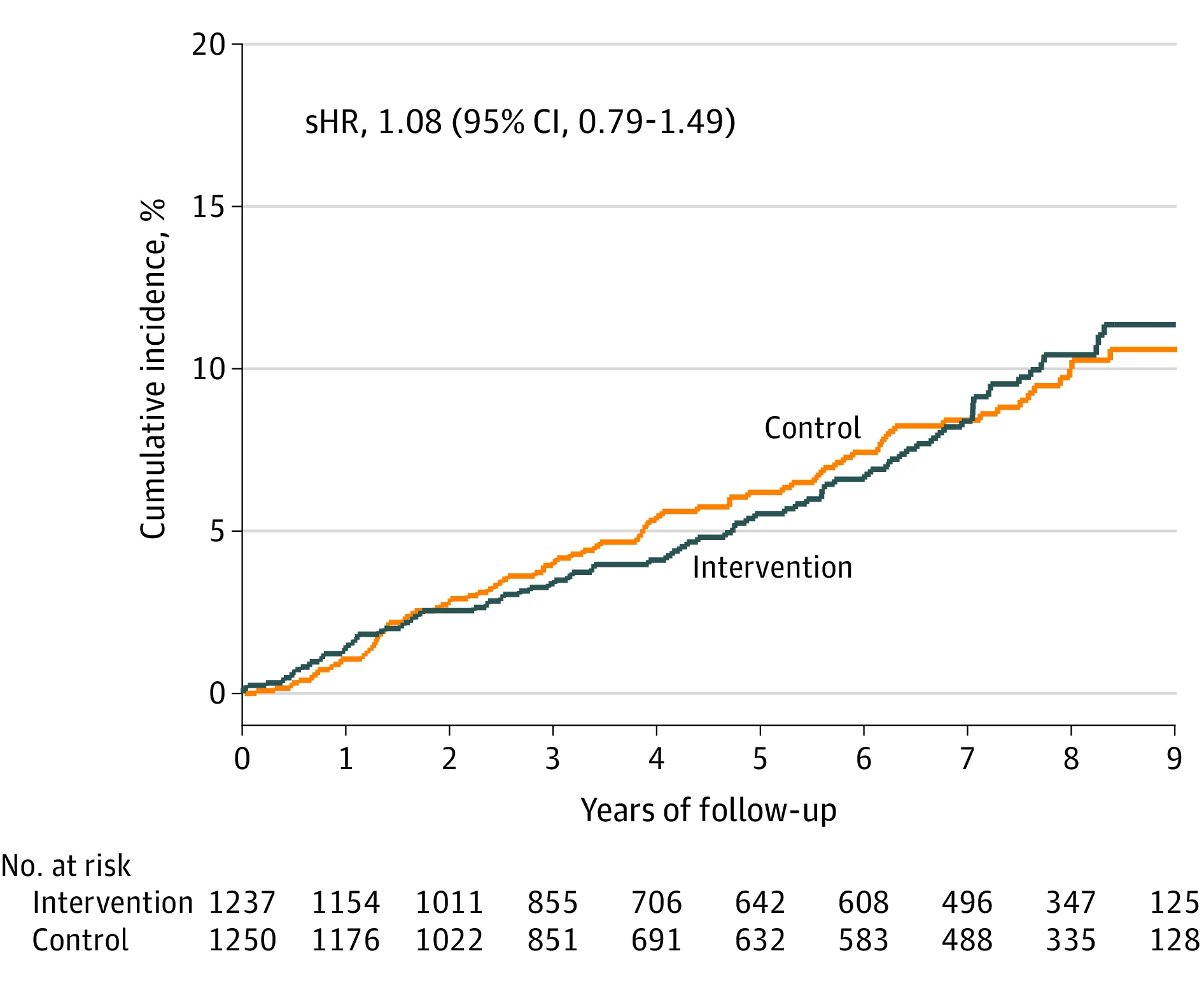
Line Graph of Cumulative Incidence Curves for the Second Primary Effectiveness Outcome Second primary outcome was cardiovascular death, myocardial infarction, stroke, and heart failure. sHR, indicates subdistribution hazard ratio.

The cumulative incidence curves of the composite of cardiovascular death, MI, stroke, and heart failure are presented in Figure 2. Despite significant improvements in cholesterol levels, no differences were observed between treatment groups for the composite outcome of cardiovascular death, MI, stroke, and heart failure (subdistribution hazard ratio, 1.08; 95% CI, 0.79-1.49; *P* = .62).

### Exploratory Analyses

For individual clinical components of the primary outcome, the subdistribution hazard ratio in the intervention group for cardiovascular death was 1.15 (95% CI, 0.73-1.82; *P* = .56), MI was 0.77 (95% CI, 0.44-1.37; *P* = .38), stroke was 1.01 (95% CI, 0.53-1.90; *P* = .98), and heart failure was 1.81 (95% CI, 0.83-3.92; *P* = .14) (eFigures 1-4 in Supplement 3). These indicate numerically higher hazards of cardiovascular death and heart failure in the intervention group but numerically lower hazards of MI, although none of these was statistically significant.

**Figure 3.**
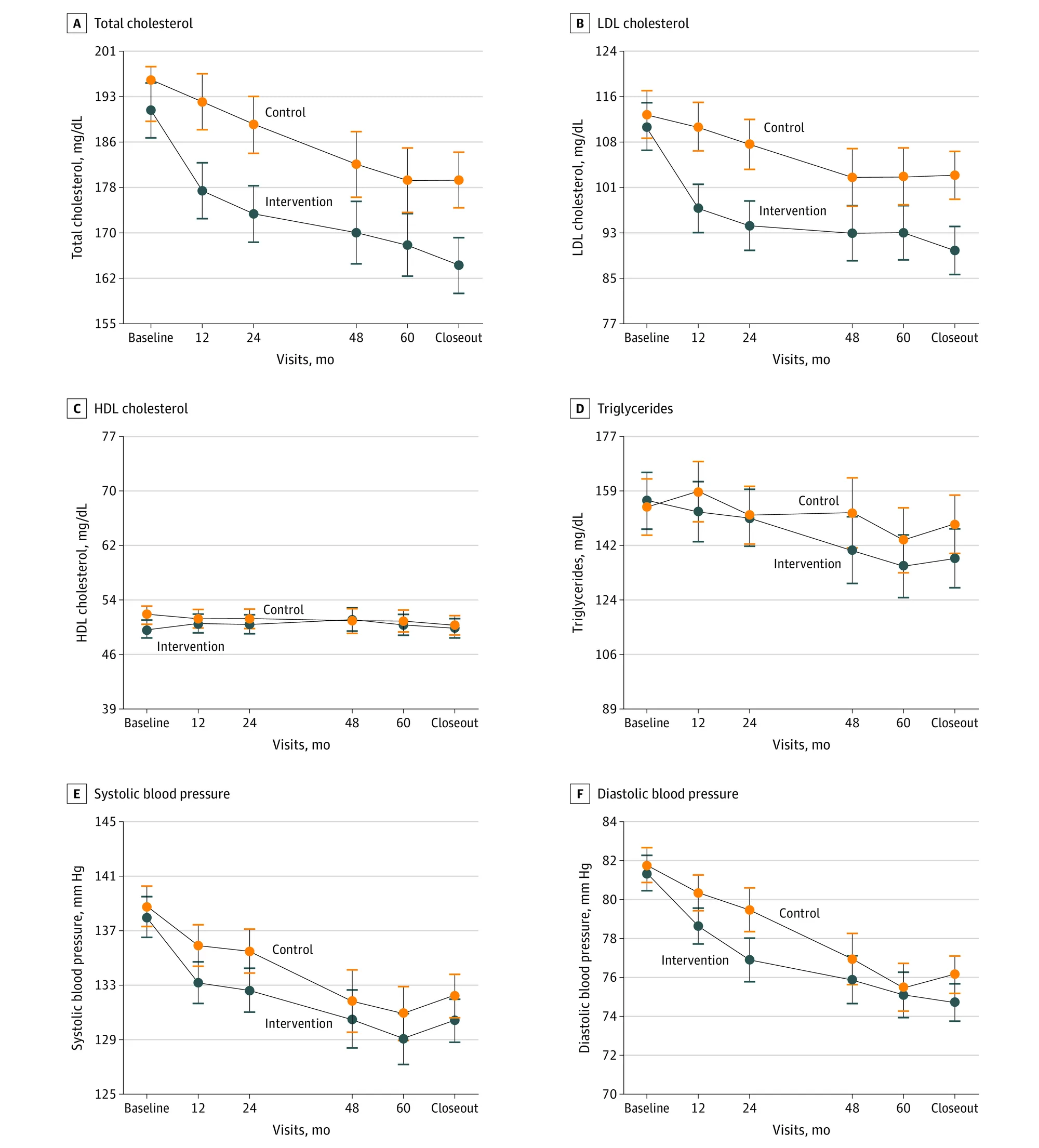
Line Graphs of the Effects of Referral to a Cardiovascular Specialist on Lipid Levels and Blood Pressure in Patients With Prostate Cancer Point estimates with 95% CIs at each study visit. To convert cholesterol levels from mg/dL to mmol/L, multiply by 0.0259; to convert triglycerides from mmol/L, multiply by 0.0113. HDL indicates high-density lipoprotein; LDL, low-density lipoprotein.

Participants assigned to an internist or cardiologist achieved lower cholesterol levels throughout the follow-up period than those receiving usual care (Figure 3). Overall, the total cholesterol concentration in the intervention group was 12.0 (95% CI, 9.0-15.0) mg/dL lower than the control group . Similar patterns were observed for LDL cholesterol and triglycerides; HDL cholesterol did not differ between groups (eTable 1 in Supplement 3).

Although systolic and diastolic blood pressure were lower in the intervention group than in the control group during the first 24 months, the difference between groups was not consistent at all time points (Figure 3). Mean (SD) close-out systolic blood pressure values were 131.1 (16.9) mm Hg in the intervention group and 132.9 (18.3) mm Hg in the control group (eTable 1 in Supplement 3); and close-out diastolic blood pressure values were 76.2 (10.4) mm Hg and 77.6 (11.2) mm Hg, respectively.

### Safety Outcomes

No cases of myositis were reported. Incidence rates per 1000 person-years for major bleeding were 0.06, kidney injury were 0.06, and liver injury were 0.01, with no between-group differences observed (eTable 2 in Supplement 3).

## Discussion

The main finding in this multicenter, international, pragmatic, randomized clinical trial of patients with recently diagnosed prostate cancer or prostate cancer that was newly treated with ADT is that routine referral to a cardiologist or internist improved cardiovascular risk factor treatment, including greater use of statin and antihypertensive medications, with lower cholesterol levels. Despite these improvements, the intervention did not reduce the incidence of major cardiovascular events during a median follow-up of 5.8 years. Importantly, observed cardiovascular event rates were substantially lower than anticipated, limiting the ability of the study to detect differences in clinical outcomes.

### Cardiovascular Events and Risk Factors

Cardiovascular risk factors are often suboptimally addressed in patients with prostate cancer. Among 90 494 US veterans diagnosed with prostate cancer, more than half of those whose glycated hemoglobin, LDL cholesterol, and blood pressure were measured had values above the thresholds of 7%, 130 mg/dL, and 140/90 mm Hg, respectively.^7^ We observed similar rates of inadequate control of cholesterol and blood pressure in patients with prostate cancer despite contact with a health care practitioner.^6^ These observations provided the rationale for evaluating whether systematic referral to a cardiologist or internist with explicit guidelines for risk reduction could improve risk factor management and, ultimately, reduce cardiovascular events.

### Effects of Referral to a Cardiovascular Disease Specialist

In this trial, routine referral to an internist or cardiologist increased the implementation of cardiovascular risk reduction strategies. Participants in the intervention group were more likely to receive statin and antihypertensive medications than those receiving usual care. These differences translated into sustained reductions in total cholesterol, LDL cholesterol, and triglyceride levels.

The favorable primary outcome was driven predominantly by improved cholesterol control. While numerical differences favored the intervention for MI and stroke, there were no statistically significant differences in any individual cardiovascular event component. Accordingly, the win ratio should be interpreted primarily as evidence of improved cardiovascular risk factor management rather than evidence of reduced cardiovascular morbidity. It is important to note that our intervention incorporated a recommendation for statin therapy, irrespective of cholesterol levels. We opted for this approach—broader than guidelines recommend—to simplify the intervention. Because the risk profile of patients who were initiated on statin therapy as part of our study is heterogeneous, this may have lessened our ability to demonstrate clinical benefit.

The disconnect between improved risk factor treatment and the absence of a reduction in cardiovascular events deserves further consideration. First, cardiovascular event rates were substantially lower than anticipated, based on prior observational studies,^2^ reducing statistical power for clinical outcomes. Second, the baseline use of statin and antihypertensive medications was already common, potentially limiting the magnitude of benefit achievable from the intervention. Third, referral to a cardiologist or internist was permissible in the control group when clinically indicated and may have attenuated the differences between cohorts. Together, these factors may have reduced the ability to demonstrate differences in cardiovascular outcomes despite significant improvements in risk factor treatment.

Importantly, the uptake of the intervention in the trial was suboptimal. The pragmatic nature of the study design illustrates the practical difficulty of addressing the holistic care of patients undergoing active cancer management, and particularly, the challenges of achieving patient adherence to treatments with a noncancer focus. A diagnosis of cancer has long been considered a teachable moment but data to support this contention are scarce.^12^

Our findings are consistent with a systematic review of the long-term adherence to physical activity regimens following an exercise intervention for patients with cancer, which demonstrated poor maintenance of activity prescriptions.^23^ Further work is needed to identify means to enhance the adoption of preventive strategies in a sustainable manner, as these will be increasingly important as cancer survivorship increases and cancer treatment options grow.

### Clinical Implications

Current clinical guidelines recommend the routine assessment and management of cardiovascular risk factors, particularly for patients treated with ADT.^14^ Our data indicate that a referral to a cardiologist or internist may be helpful in this context to address dyslipidemia and hypertension. However, we could not show that cardiovascular specialist referral reduced adverse cardiovascular events, suggesting that universal referral may be unnecessary. Rather, selective referral of individuals with poor risk factor control may provide the greatest value.

### Limitations

The principal limitation of the trial is the lower than expected incidence of cardiovascular events, which limited the power to detect differences in clinical outcomes. Crossovers, including the adoption of components of the intervention in the control group and nonadherence to the intervention group, further reduced study power. As a result, we cannot exclude a clinically meaningful effect on clinical outcomes—either beneficial or harmful—of routine referral to a cardiovascular specialist. However, the absence of an observed treatment effect on cardiovascular events should not be interpreted as evidence that improving cholesterol levels and blood pressure control lacks clinical benefit.

In addition, we cannot rule out the possibility that the open-label design of this pragmatic trial led to differential ascertainment of outcomes between the intervention and control groups. We attempted to minimize this possibility by the use of prespecified clinical outcome event definitions and blinded adjudication of all clinical outcomes. Nevertheless, mild heart failure may have been identified more readily and death may have been more frequently attributed to cardiovascular conditions among patients being treated by a cardiologist than in those who were not.

Given that this was a pragmatic trial, patients’ primary care physicians were not given specific directives. It is unknown whether the improvements in cholesterol concentrations observed in the intervention group, or indeed improvements in the other outcomes, could be achieved by closer engagement of primary care physicians. Lastly, White patients were overrepresented in this trial, so the effects of the intervention among other racial and ethnic groups may differ from our findings.

## Conclusions

In this randomized clinical trial, routine referral of patients with newly diagnosed prostate cancer or those starting ADT to a cardiovascular specialist or internist leads to improved control of cholesterol levels; however, this did not translate to a reduction in cardiovascular death, MI, stroke, or heart failure.

## Notes

Supplement 1. Trial Protocol, version 6.0

Supplement 2. Statistical Analysis Plan

Supplement 3. eTable 1. Lipid and Blood Pressure Values eFigure 1. Clinical Event Cumulative Incidence Curves for Cardiovascular Death (CVD) eFigure 2. Clinical Event Cumulative Incidence Curves for Myocardial Infarction (MI) eFigure 3. Clinical Event Cumulative Incidence Curves for Stroke eFigure 4. Clinical Event Cumulative Incidence Curves for Heart Failure (HF) eTable 2. Safety Outcomes eAppendix

Supplement 4. Nonauthor Collaborators

Supplement 5. Data Sharing Statement
