# Supplementary material for: Specialist Referral for Cardiovascular Risk in Patients With Prostate Cancer: A Randomized Clinical Trial
Source: JAMA Intern Med. 2026 Aug 30:e264773. Online ahead of print. doi: 10.1001/jamainternmed.2026.4773 (PMC13527639; doi:10.1001/jamainternmed.2026.4773)
Supplement: Supplementary file 1 [file jamainternmed-e264773-s001.pdf]

1. The Role of Androgen Deprivation Therapy In CArdiovascular Disease – A Longitudinal Prostate Cancer Study (RADICAL PC1)

2. A RAndomizeD Intervention for CArdiovascular and Lifestyle Risk Factors in Prostate Cancer Patients (RADICAL PC2)

RADICAL PC1 is a prospective cohort study of men with a new diagnosis of prostate cancer

RADICAL PC2 is a randomized, controlled trial of a systematic approach to modifying cardiovascular and lifestyle risk factors in men with a new diagnosis of prostate cancer

Sponsor and Coordinating Centre:

RADICAL PC
Population Health Research Institute
Hamilton General Hospital Campus, DBCVSRI
237 Barton Street East, Hamilton, Ontario, Canada
L8L 2X2

Tel: 905-527-4322
Fax: 905-297-3779
radicalpc@phri.ca

FINAL Version 6.0, 2025-12-23

This protocol has been developed by the RADICAL PC Operations Committee and its contents are the confidential intellectual property of this group. It is an offence to reproduce or use the information and data in this protocol for any purpose other than the RADICAL PC Trial without prior approval from the Project Office of the RADICAL PC Trial.

INVESTIGATOR'S AGREEMENT

I, ____, the investigator, have examined this RADICAL PC trial protocol and have fully discussed the objectives of this trial and the contents of this protocol with the RADICAL PC Coordinating Centre representative(S) from the Population Health Institute at Hamilton Health Sciences Corporation (Sponsor).

I agree to conduct the study according to this protocol and to comply with its requirements, subject to ethical and safety considerations.

I agree to comply with the International Council for Harmonization Tripartite Guideline on Good Clinical Practice (GCP), Tri-Council Policy Statement (TCPS2) and other applicable guidelines, and all applicable laws and regulations.

I agree to ensure that the confidential information contained in this document will not be used for any purpose other than the evaluation or conduct of the clinical investigation without the prior written consent of the Sponsor.

I agree the Sponsor owns all data and intellectual property generated from this trial. I further agree that any presentation or publications relating to the trial must be approved by the Trial Steering Committee.

I acknowledge the Sponsor will indemnify my hospital and I for any third-party claims resulting directly from PHRI's negligence or wilful misconduct in this trial, provided that such claim is not caused by my hospital's or my negligence or wilful misconduct.

Investigator Name: ____

Investigator Signature: _______________________ **Date**: ______________

Facility/Site: ____

Country: ____

PROTOCOL APPROVAL

Signature: Date:

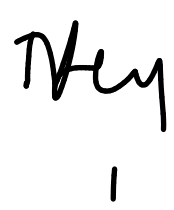

Electronically signed by: Darryl Leong
Reason: I approve this document
Date: Jan 5, 2026 19:12:15 EST

2026-01-05

Darryl Leong
Population Health Research Institute, Hamilton Health Sciences

Signature: Date:

Jehonathan Pinthus

Electronically signed by: Jehonathan Pinthus
Reason: I approve this document
Date: Jan 6, 2026 09:50:40 EST

2026-01-06

Jehonathan Pinthus
Hamilton Health Sciences

ABBREVIATIONS

ACE-I = angiotensin-converting enzyme inhibitor

ADT = androgen deprivation therapy

CRF = case report form

CV = cardiovascular

CVD = cardiovascular disease (coronary artery disease, cerebrovascular disease, heart failure, or cardiovascular death)

DSMB = Data Safety Monitoring Board

DSS = digital symbol substitution

ECOG = Eastern Cooperative Oncology Group performance status

FFQ = food frequency questionnaire

FSH= follicle stimulating hormone

GnRH = gonadotropin-releasing hormone

LDL = low-density lipoprotein cholesterol

MACE = major adverse cardiovascular events (any of myocardial infarction, stroke, heart failure, cardiovascular death, need for arterial revascularization, or hospitalization for unstable angina)

MI = myocardial infarction

PC = prostate cancer

PHQ-9 = Patient Health Questionnaire-9

PSA = prostate-specific antigen

SAGE = Standard Assessment of Global Activities in the Elderly

STUDY FLOW CHART

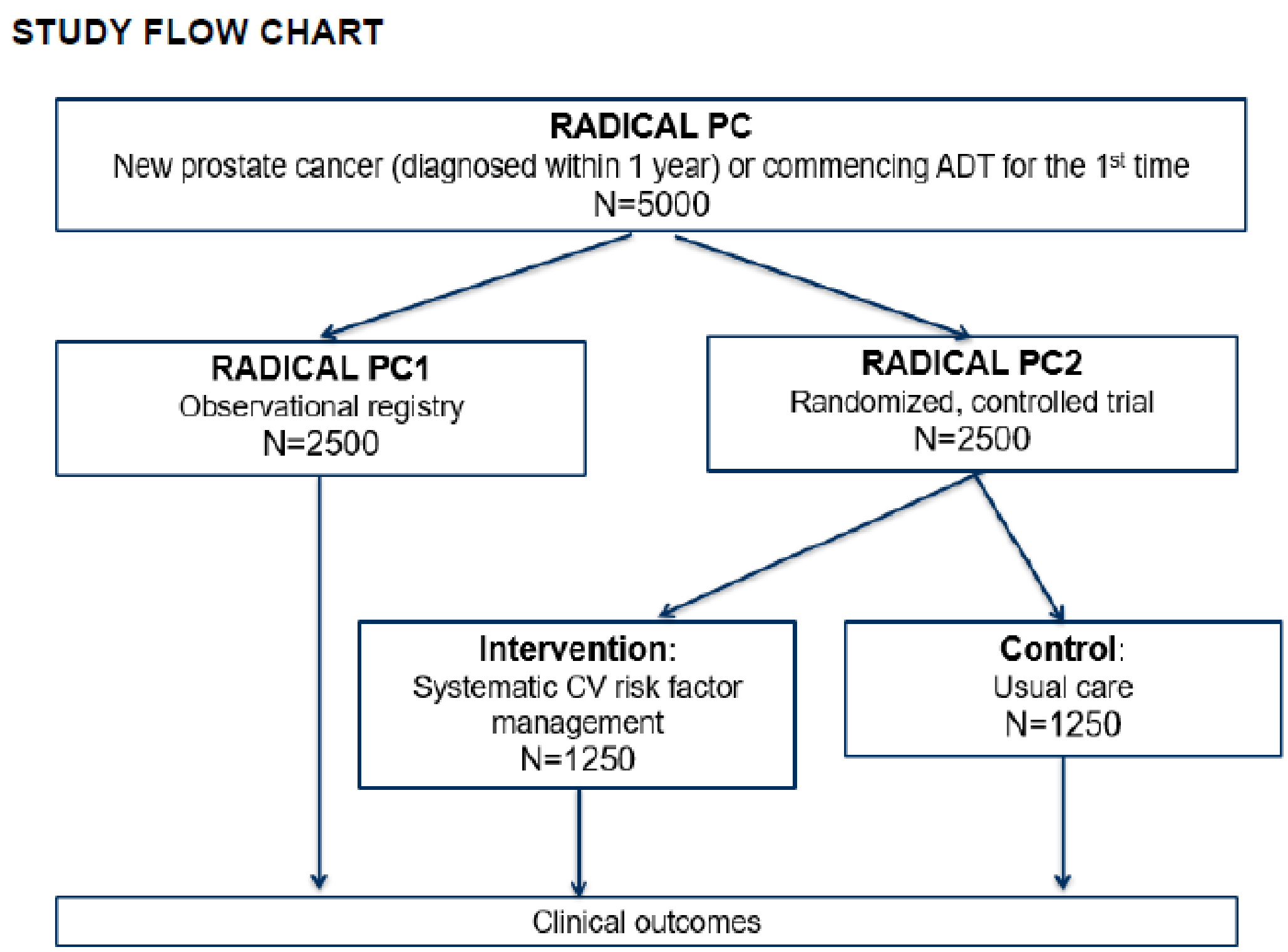

Table of Contents

1.0 OVERVIEW 9
2.0 BACKGROUND 9
3.0 OBJECTIVES – RADICAL PC1 21
3.1 Co-Primary Objectives 21
3.2 Secondary Objectives 21
4.0 OBJECTIVES – RADICAL PC2 22
4.1 Primary Objective 22
4.2 Secondary Objective 22
5.0 SPECIFIC HYPOTHESES – RADICAL PC1 22
5.1 Primary Hypothesis 22
5.2 Secondary Hypotheses 23
6.0 HYPOTHESES – RADICAL PC2 23
6.1 Primary Hypothesis 23
6.2 Secondary Hypotheses 23
6.3. Tertiary Hypotheses 23
7.0 STUDY DESIGN – RADICAL PC 1 24
8.0 STUDY DESIGN – RADICAL PC2 24
9.0 ENDPOINTS FOR RADICAL PC1 26
9.1 Primary Endpoint 26
9.2 Secondary Endpoints 26
9.3 Tertiary Endpoints 26
10.0 EFFECTIVENESS ENDPOINTS FOR RADICAL PC2 27
10.1 Powered First Primary Endpoint 27
10.2 Second Primary Endpoint 27
10.3 Exploratory analyses 27
11.0 SAFETY OUTCOMES 27
12.0 INTERVENTIONS IN RADICAL PC2 28
13.0 ELIGIBILITY CRITERIA 30
13.1 Inclusion Criteria 30
13.2 Exclusion Criteria 30

14.0 PATIENT SCREENING AND RECRUITMENT 31
15.0 STUDY PROCEDURES 31
16.0 STUDY INTERVENTION ADHERENCE 37
17.0 GUIDANCE FOR THE IMPLEMENTATION OF STUDY INTERVENTIONS 37
18.0 STUDY TIMELINE 40
19.0 COMMITTEES 40
20.0 STUDY COORDINATION AND EXECUTION 41
21.0 DETERMINATION OF SAMPLE SIZE 41
21.1 RADICAL PC1 41
21.2 RADICAL PC2 41
22.0 STATISTICAL ANALYSIS 43
23.0 CLINICAL EVENT DEFINITIONS 49
References 58

1.0 OVERVIEW

This study protocol describes two prospective studies, one of which is embedded in the other. RADICAL PC1 is an unbiased prospective cohort study of men within one year of their first diagnosis of PC, or who are commencing ADT for the first time. Its goal will be to identify factors associated with the development of CVD among men with PC, with a particular focus on ADT. RADICAL PC2 is a randomized, controlled trial in which eligible RADICAL PC1 participants will be enrolled.

RADICAL PC2 will test the effect of routine referral of men to a cardiologist or internist within one year of their first diagnosis of PC or who are commencing ADT for the first time, compared to usual care. In the intervention arm, the cardiologist or internist, will implement a systematic approach to modifying CV and lifestyle risk factors. Potentially eligible men identified at screening will be invited to participate in RADICAL PC1 or RADICAL PC2. The analysis of data from RADICAL PC1 will include data from RADICAL PC2.

2.0 BACKGROUND

The Epidemiology of CVD in Men with PC

CVD frequently affects men with PC. In a Swedish population study of 57,187 men with localized PC, CVD was the most common cause of death in men with a Charlson co-morbidity index ≥2, and was also the most common cause of death in those men with low-risk PC and a Charlson co-morbidity index of 0[1]. In a US Veterans Healthcare study of 37,443 population-based men with local or regional PC, the incidence rates and 95% confidence intervals per 1000 person-years of coronary heart disease, MI, sudden cardiac death and stroke from 2001-04 are displayed in Table 1[2].

Table 1 – Incidence of specific types of CVD per 1000 person-years in men with PC, stratified by ADT use and type

Treatment	Coronary heart disease	MI	Sudden cardiac death	Stroke
**No ADT**	81.4 (78.7-84.2)	7.3 (6.4-7.9)	11.5 (10.7-12.3)	10.8 (10.0-11.5)
**GnRH agonist**	144.0 (135.7-152.2)	12.8 (11.1-14.4)	21.6 (19.4-23.7)	18.5 (16.5-20.5)
**Orchiectomy**	210.5 (150.9-270.0)	24.3 (12.4-36.3)	23.3 (11.5-35.1)	26.2 (13.8-38.7)
**Combined androgen blockade**	157.7 (129.4-186.0)	10.2 (5.2-15.2)	20.1 (13.0-27.2)	14.8 (8.8-20.9)
**Oral antiandrogen**	143.2 (97.1-189.4)	11.2 (2.3-20.1)	18.8 (7.2-30.5)	14.9 (4.6-25.2)

In a Swedish population study of 41,362 men with PC, CVD was defined according to the International Classification of Diseases 10th Revision codes I00 to I99. The incidence of CVD per 1000 person-years was 139.7, 216.4, and 234.3 among those treated with antiandrogens, GnRH agonists, and orchiectomy respectively[3]. In this population, the incidence of ischemic heart disease, heart failure, and stroke is presented in Table 2.

Table 2 – Incidence of specific types of CVD per 1000 person-years in men with PC within 2 years before and after initiation of ADT[3].

**CVD Type**	**Incidence per 1000 person-years**		
	Antiandrogen	GnRH agonist	Orchiectomy
**For the 2 years before initiation of ADT**			
**Ischemic heart disease**	15.8	22.4	22.0
**Heart failure**	5.9	10.9	14.4
**Stroke**	13.2	17.8	17.3
**For the 2 years after initiation of ADT**			
**Ischemic heart disease**	20.4	30.1	31.2
**Heart failure**	10.3	24.6	27.0
**Stroke**	15.8	24.4	26.6

In a recent Kaiser Permanente Southern California database study of 7637 men with newly diagnosed, localized PC, the crude incidence rates of ischemic heart disease, stroke and heart failure were respectively 17.7, 2.3 and 22.0 per 1000 patient-years (where each of these categories was mutually exclusive)[4].

The American Heart Association considers a 20% 10-year risk of coronary heart disease to be high[5]. Thus, men with PC as a group are at high risk of CVD.

Explaining the High CVD Risk among Men with PC

The reasons underlying the high CVD risk among men with PC are unclear. Possible explanations include 1) the common determinants of CVD and PC, 2) improved survival with PC, which leaves the individual at risk of other illnesses such as CVD (whereas they might previously have been more likely to die from their PC), 3) CV risk associated with PC therapies, and 4) CV effects of the PC itself.

The Potential Role of ADT in Promoting CVD

The earliest experience in androgen deprivation in men with PC occurred in 1941, when Huggins *et al.* reported on 21 men with PC who underwent orchiectomy[6]. Since then, GnRH agonists have emerged as the most frequently prescribed means of ADT, as they have been shown to reduce the risk of PC-specific and all-cause mortality in men with non-metastatic PC[7], while avoiding the need for surgical castration. Moreover, ADT for 4-6 months has been demonstrated to improve overall survival when used in addition to conventional-dose radiotherapy in men with intermediate- and high-risk T1/T2 PC (i.e. where

the cancer has not spread beyond the prostate capsule). Consequently, ADT is prescribed in up to 40% of men within 6 months of PC diagnosis[8]. ADT is also frequently prescribed to men with biochemical failure (rising PSA) following definitive therapies such as radical prostatectomy or radiation therapy.

ADT has been implicated as a risk factor for CVD. A meta-analysis of six population-based observational studies, including 119,625 ADT recipients and 150,974 ADT non-recipients demonstrated a hazard ratio (95% confidence interval, CI) for cardiovascular mortality of 1.17 (1.04-1.32; p=0.01) among ADT recipients[9]. A separate, recent meta-analysis of eight observational studies of ADT demonstrated that GnRH agonists (relative risk, RR, 1.38; 95% CI 1.29-1.48), orchiectomy (RR 1.44, 95% CI 1.28-1.62), and anti-androgens (RR 1.21, 95% CI 1.07-1.37) were associated with an increased risk of non-fatal CVD[10]. The findings from these observational studies contrast with an earlier meta-analysis of randomized trials of ADT, which demonstrated no excess cardiovascular death in the ADT arms in over 4000 participants who experienced more than 500 cardiovascular deaths[7]. In this meta-analysis, ADT exhibited a relative risk for cardiovascular death of 0.93 (95% confidence interval 0.75-1.10) as compared with control.

There is ongoing speculation as to the reasons underlying the divergent conclusions of observational studies and randomized trials. Observational studies are limited by residual confounding. Indeed, the existing observational evidence linking ADT use with CVD comprises largely data from administrative databases, which are limited in the covariates recorded. The possible role of confounding in explaining the findings from observational studies is illustrated in the study by Kim *et al*[11]. In this study from the British Columbia Cancer Agency (which was not included in the studies cited in the two meta-analyses of observational studies of ADT and CVD[9,10]), the investigators found that ADT use was associated with a *lower* incidence of CV death than non-use of ADT in men with PC. The reduced risk of CVD death associated with >6 months ADT use persisted after adjustment for duration of ADT, PC stage, radiation dose administered and age at PC diagnosis (p=0.001)[11]. Importantly, adjustment was not systematically made for cardiovascular risk factors, and it is unknown whether the protective association seen between ADT use and CVD death would have been abolished had cardiovascular risk factors been accounted for. Based on a *post hoc* analysis of a subgroup of individuals in the study, the authors speculated that the inverse association between ADT use and CVD risk may have been explained by the higher prevalence of CV risk factors and higher Charlson scores observed among men who did not receive ADT[11].

The meta-analysis of trials of men with non-metastatic, non-hormone refractory PC, in which participants were randomized to an ADT arm or a control group, demonstrated a 14% (95% CI 7%-20%) reduction in all-cause mortality, and a 31% (95% CI 16%-44%) reduction in PC-specific mortality in the ADT arms[7]. This study also found no increase in the risk of CV death in the ADT groups (relative risk 0.93, 95% CI 0.79-1.10), with 255 of 2200 in the ADT groups experiencing CV death, as compared with 252 of 1941 in the control groups. While this

evidence suggests that an excess of CV mortality among ADT recipients is unlikely, the randomized trials have limitations with respect to this outcome. Among the eight randomized trials of ADT, four featured control arms that received ADT in a deferred manner, which may have diluted any adverse cardiovascular effects of ADT. Cardiovascular outcomes were not well characterized in the randomized ADT trials, and ascertainment of adverse cardiovascular events may not have been systematic, thus reducing the power of these trials to detect an effect of ADT on CVD incidence. Blinding was not performed in any of the trials included in the meta-analysis. Owing to the tendency to recruit men who are otherwise healthy into randomized trials, men with greater co-morbidity, including CVD, may have been excluded from these trials. Indeed, the largest burden of CVD death in men with PC is seen in those with greater co-morbidity. For these reasons, there remains uncertainty about the role, if any that is attributable to ADT in promoting CVD.

Potential Mediators of the Association between ADT and CVD

ADT is thought to induce metabolic changes that are known CVD risk factors. ADT is associated with dysglycemia[12], which is a risk factor for CVD. ADT has been linked with dyslipidemia[12], which is a major cause of coronary artery disease[13]. ADT use has also been associated with sarcopenia[14,15]. We have recently demonstrated that lower muscle strength, as evaluated by handgrip strength, is a powerful predictor of incident CVD and cardiovascular mortality[16]. ADT use has been associated with increased adiposity[14,15], especially of a central distribution[15]. Central adiposity has been shown to be an important risk factor for myocardial infarction[17,18]. ADT has also been demonstrated to promote arterial stiffness[14]. Thus there appear to exist biologically plausible mechanisms through which ADT might promote CVD. Importantly, the evidence to implicate these pathological pathways is nearly entirely from observational evidence; there is a paucity of data from the randomized trials of ADT to either implicate or exclude the role of ADT in promoting CVD risk factors, which may mediate an association between ADT and CVD[19].

Experimental evidence using androgen receptor knockout mice implicates androgen deficiency directly in hyperglycemia, insulin resistance, and visceral fat accumulation[20], which are hallmarks of the metabolic syndrome – a phenotype that is strongly linked to the development of CVD. In a mouse model of atherosclerotic disease, we have recently shown that ADT, using gonadotropin releasing hormone (GnRH) agonist or orchiectomy and to a lesser extent GnRH antagonist, causes obesity, sarcopenia, dysglycemia and atherosclerosis compared to control mice[21]. Hence our preclinical experience is consistent with an increased risk of CVD in recipients of ADT.

Finally, one should not assume necessarily that all types and modalities of ADT result in a similar metabolic and cardiovascular risk profile. While it is unknown yet if the application of intermittent androgen deprivation translates to lower rates of metabolic and cardiovascular complications compared to continuous androgen deprivation, data from mice models suggest that GnRH

antagonists may be associated with less weight gain and adiposity, higher lean body mass, and more favourable glucose tolerance tests than GnRH agonists or orchiectomy[21]. In this study, the aortic atherosclerotic plaque volume and percentage of necrotic core within it (a marker of plaque vulnerability) were significantly lower in mice treated with GnRH antagonists (Degarelix) compared to GnRH agonists (Leuprolide) or orchiectomy. Preliminary mechanistic data suggest that elevated serum FSH levels in patients treated by ADT may explain the differences in atherogenicity between the different ADT modalities. These experimental findings are consistent with limited data from randomized trials comparing GnRH agonists with GnRH antagonists. In a pooled analysis of six trials in which men with PC were randomized to GnRH agonist or GnRH antagonist, GnRH antagonist use resulted in a significantly lower risk of cardiac adverse events at one year (hazard ratio 0.44, 95% CI 0.26-0.74, p=0.002)[22].

CVD, Complex Multi-morbidity, and Function in Men with PC

There is a high prevalence of multi-morbidity among men with PC because PC affects predominantly older individuals, and because PC is most often a slow-growing cancer, rather than a rapidly lethal one, thus allowing time for the accumulation of co-morbid conditions (some of which may be related to the PC or its treatment, as described previously). This multi-morbid state may lead to loss of function (physical, cognitive, and psychological), which in turn may adversely influence PC treatments, and clinical outcomes[23]. Studies to date investigating the prevalence, incidence, determinants, and prognostic implications of loss of function in men with PC have in general been small, with limited power to address these questions[24-26]. A recent systematic review of clinical trials on the supportive care of men with PC found existing studies to be underpowered, poorly representative, and to be lacking in cost data[27].

Frailty, which may be defined as a state of reduced physiological reserve, and increased vulnerability to poor resolution of homeostasis after a stressor event, is common in older individuals with CVD. Men with PC, by virtue of their age, and of PC therapies including ADT, are likely to suffer from frailty. There is a strong and independent association between frailty, and CVD morbidity and mortality[28,29]. An adequate understanding of ADT, CVD and clinical outcomes in PC therefore requires characterization of physical, cognitive, and psychological function, in order to understand the prevalence, incidence, and implications of frailty in men with PC. Indeed, there have been attempts to characterize the frail phenotype. Fried *et al.* studied 5317 adults aged ≥65 years, who were followed for up to eight years[29]. Frailty was defined as the presence of at least three of the following markers: unintentional weight loss (≥10 pounds in the year prior to enrolment, or ≥5% during follow-up), low grip strength, self-reported exhaustion, slow gait speed, and low physical activity levels. Compared with the absence of frailty, frailty was associated with an adjusted hazard ratio (95% confidence interval) for a first hospitalization of 1.27 (1.11-1.46), and for death of 1.63 (1.27-2.08).

Addressing CV Risk in Men with PC

Irrespective of any role of ADT in promoting CVD, recognition is growing of the prognostic importance of CVD in men with PC. In an unpublished analysis of the US SEER database, we have shown that early after the diagnosis of PC, the risk of PC-death dominates clinical outcomes, however this is soon superseded by the risk of CV death (Figure 1). ***There is a paucity of specific evidence on how the risk of CVD can be reduced in men with PC.***

In the general population, much of the population attributable risk of ischemic heart disease is accounted for by nine modifiable risk factors[30]. The INTERHEART study demonstrated that smoking, abdominal obesity, blood cholesterol, hypertension, psychosocial factors, dietary factors, alcohol use, and physical inactivity accounted for at least 90% of the population attributable risk of acute myocardial infarction[30]. The reduction of these risk factors is thought to account for a substantial proportion of the reduction in CVD mortality observed in North America, and other countries[31]. The extent to which CV risk factor reduction in men with PC will improve clinical outcomes is unknown, however. Existing paradigms on CV risk factor modification should not necessarily be extrapolated to men with PC because they differ from the general population. Men with PC suffer from the competing risk of PC-death, and morbidity from the adverse effects of PC treatment. There is therefore a need to demonstrate the efficacy of CV risk factor modification in men with PC, as a high-risk, but unique and under-studied population.

The following paragraphs describe the current state of knowledge of the importance and treatment of common, modifiable CV risk factors.

Figure 1. Hazard of CV and PC death as a function of time from PC diagnosis in men from the SEER registry.

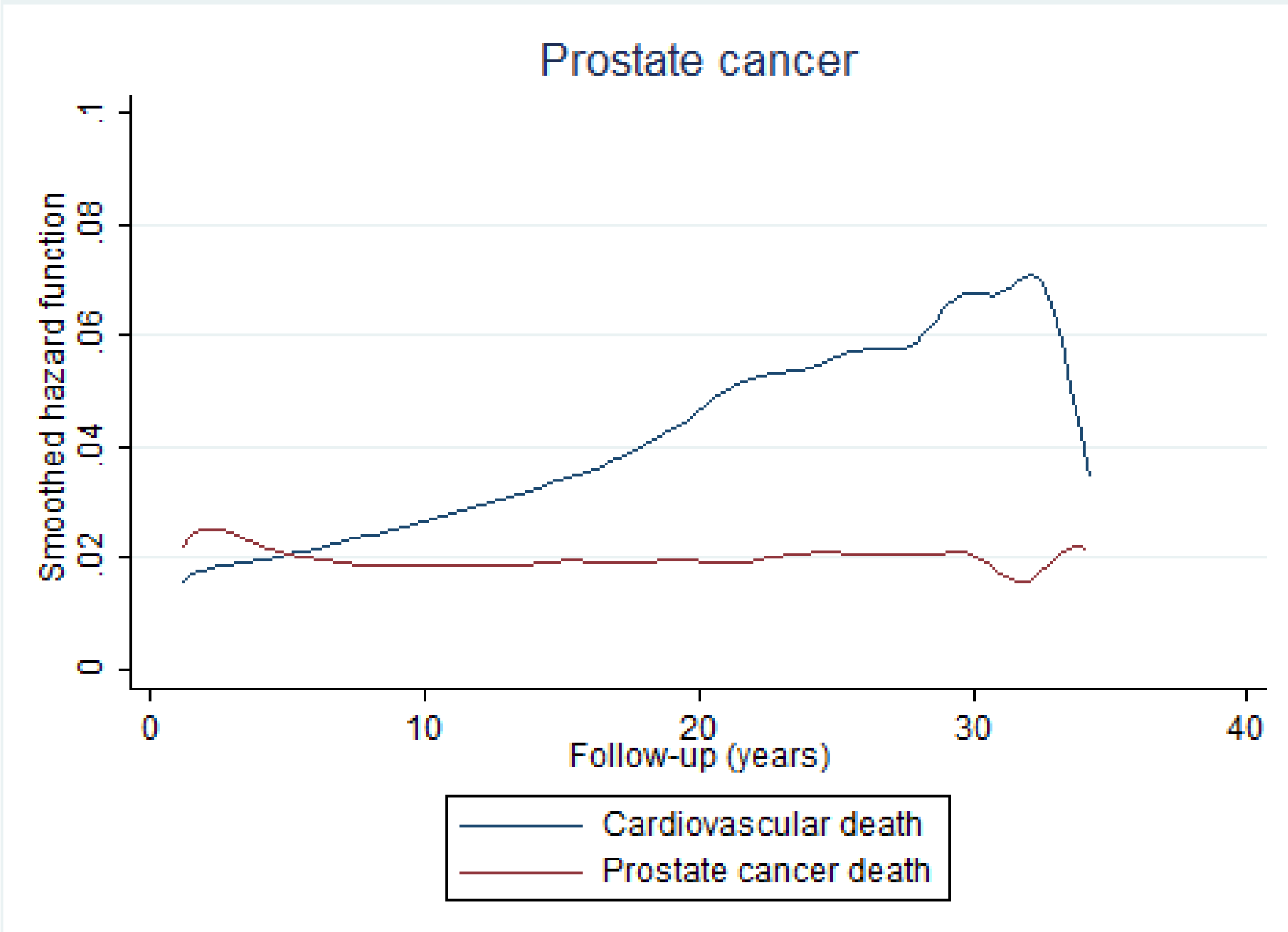

Cholesterol and CV Risk

There is a strong, continuous positive relationship between cholesterol concentrations and the risk of ischemic heart disease[13,32]. A meta-analysis of randomized controlled clinical trials of cholesterol lowering treatment, which included individual data from over 90,000 people who participated in trials evaluating various statins, demonstrated reductions of 12% in all-cause mortality, 23% in major coronary heart disease events, 26% in myocardial infarction, and 17% in strokes per 1 mmol/L LDL cholesterol reduction[33].

As a consequence of this large body of evidence on the prognostic importance of blood cholesterol, and on the CV benefits of cholesterol reduction in the appropriate individual, the current Canadian Cardiovascular Society guidelines recommend screening of plasma lipid concentrations in men aged ≥40 years[34]. They also recommend serum lipids, blood glucose, and estimated glomerular filtration rate be measured annually if the Framingham Risk Score is ≥5%. Lipid-lowering using statins (3-hydroxy-3-methylglutaryl–coenzyme A reductase inhibitors) is suggested for those with a Framingham Risk Score ≥20%[34].

There is scant direct evidence to suggest that men with PC will derive the same benefit from statins as do the general population, or whether the competing risk of PC death will limit any beneficial effect of statins. Moreover, statins are known to cause an increase in the risk myositis[35] and myalgia[36]. In a randomized trial designed to evaluate the myopathic effects of statins, atorvastatin 80mg daily

resulted in an increase in serum creatine kinase concentrations, and myalgia (9.4% versus 4.6%, p=0.054) as compared with placebo[36]. PC treatment using ADT may lead to loss of skeletal muscle mass as previously described, and it is unknown whether sarcopenia increases susceptibility to statin-induced myositis.

Thus, there is equipoise as to whether routine use of statins in men with PC will result in net clinical benefit. Interestingly, a recent systematic review and meta-analysis reported an association between statin use and a lower risk of PC recurrence (hazard ratio 0.68, 95% CI 0.49-0.93) in six observational studies examining the relationship between statin use and PC recurrence following radiotherapy[37]. The authors speculate that any benefit of statins on PC outcomes may be related to a radiation-sensitizing effect of statins. This emerging evidence adds to the importance of testing the effects of statins in men with PC.

Recent evidence suggests that patients with prostate cancer exhibit cholesterol concentrations above guideline-recommended targets in 51% of cases[38].

Hypertension and CV Risk

Epidemiological studies show a strong, linear relationship between elevations in blood pressure and risk of stroke, ischemic heart disease, and heart failure[39-41]. A meta-analysis of trials of blood pressure-lowering medication suggests that larger degrees of blood pressure lowering are associated with greater reductions in the risk of coronary heart disease and stroke[42]. However, some trials have suggested a "J" shaped relationship between blood pressure (reduction) and the risk of adverse cardiovascular outcomes[43]. There is clearly a lower limit of blood pressure beyond which further blood pressure reduction is detrimental, however it remains uncertain precisely what the optimal blood pressure is, and whether different populations or patient groups have the same optimal blood pressure. Randomized blood pressure-lowering trials have yielded mixed conclusions with respect to target blood pressure. In a randomized trial of 4418 individuals aged 65-85 years with hypertension, "strict" treatment to a target systolic blood pressure <140mmHg offered no advantage over "mild" treatment to a target systolic blood pressure <160mmHg[44]. In contrast, a trial of 1111 non-diabetic patients with hypertension randomized to usual control (target systolic blood pressure <140mmHg) or tight control (target systolic blood pressure <130mmHg) demonstrated a reduction in the composite secondary outcome with hazard ratio 0.50 (95% confidence interval 0.31-0.79)[45]. This risk reduction was driven in large part by a reduction in the need for coronary revascularization (absolute risk reduction 1.8%). The recent SPRINT trial, which included adults ≥50 years of age with a systolic blood pressure 130-180mmHg, and a 10-year cardiovascular risk of ≥15%, demonstrated that a target systolic blood pressure of 120mmHg, compared with a target of 140mmHg, was associated with a significant reduction in the incidence of all-cause mortality (hazard ratio 0.73, 95% confidence interval 0.60-0.90, p=0.003) and of the primary composite outcome of myocardial infarction, acute coronary syndrome, stroke, acute decompensated heart failure, or cardiovascular death (hazard ratio 0.75, 95% confidence interval 0.64-0.89,

p<0.001)[46]. The HOPE-3 trial of adults with a 10-year CVD risk of approximately 10% randomly assigned participants to receive fixed-dose angiotensin receptor-blocker and thiazide versus placebo. Despite achieving lower blood pressure, the intervention group did not experience a reduction in CVD compared with the placebo group[47]. Based on the evidence available at the time of writing (which did not include the findings of the HOPE-3 trial), the 2016 Canadian Hypertension Education Program recommends antihypertensive drug therapy for blood pressure with target blood pressure <140/90mmHg, and that in selected high-risk individuals, lower targets may be appropriate[48]. The Eighth Joint National Committee on Blood Pressure recommends drug therapy for blood pressure ≥150/90mmHg in the general population aged ≥60 years, and for blood pressure ≥140/90 in the general population aged <60 years[49]. More recent European guidelines recommend target systolic and diastolic blood pressure values of 120-129mmHg and 70-79mmHg respectively, while more relaxed systolic values <140mmHg might be acceptable in those >80 years or frail[53].

In selected individuals with controlled blood pressure, ACE-I in particular may have beneficial effects. The HOPE study was a randomized, controlled trial of the ACE-I ramipril versus placebo in individuals with high CV risk (but no left ventricular dysfunction). Ramipril was shown to reduce the risk of death, myocardial infarction, or stroke[50]. In this trial, the relative risk (95% confidence interval) of the composite endpoint of MI, stroke, or cardiovascular death in the ramipril arm was 0.78 (0.70-0.86). Among participants in the HOPE study with controlled blood pressure (131/76mmHg), the use of ramipril resulted in favourable reverse left ventricular modelling, with a reduction in left ventricular mass demonstrated[51]. In the EUROPA study, among individuals with stable coronary artery disease, the use of the ACE-I, perindopril, resulted in a 20% (95% confidence interval 9-29%) reduction in CV death, myocardial infarction, or cardiac arrest[52]. In this trial, the average baseline blood pressure was also within normal limits (137/82mmHg).

The PEACE trial of patients with stable coronary artery disease randomized participants to the ACE-I trandolapril or placebo[53]. Forty-six percent of participants had hypertension. In this study, trandolapril did not result in a significant reduction in the primary composite endpoint of CV death, MI, or coronary revascularization after a median 4.8 years. The hazard ratio in the trandolapril group was 0.96 (95% confidence interval 0.88-1.06). Thus while there is some evidence to suggest that ACE-I in individuals at high cardiovascular risk may be of benefit even if blood pressure is within normal limits, clinical trials of ACE-I have not produced consistent findings in this context.

A meta-analysis of seven trials in which 41,931 participants with hypertension or high CVD risk (but without heart failure) were randomized to receive an ACE-I or placebo demonstrated that ACE-I resulted in a 21% reduction in the risk of incident heart failure (pooled random effects odds ratio 0.79, 95% confidence interval 0.66-0.94). This collective evidence suggests that ACE-I may be effective at the prevention of heart failure in individuals with hypertension or at high CVD risk.

ACE-I are well tolerated and have an excellent safety record. In the EUROPA trial of 6119 individuals with stable coronary artery disease, there was no difference in the incidence of renal failure between the ACE-I group and the placebo group (both exhibited incidence rates of renal failure of 0.3% over more than three years' follow-up)[52]. In the SOLVD trial of 2569 patients with severe heart failure, the ACE-I, enalapril, led to only small increases in serum potassium concentration (0.2mmol/L) and in serum creatinine concentration (0.0088mmol/L) compared to the placebo group[54]. In this study, there was no difference in mortality rates attributable to renal failure or hyperkalemia between the groups, although there was a substantial advantage in favour of enalapril with respect to overall mortality[54]. In a study of 1818 ambulatory patients who were prescribed ACE-I, the incidence of a serum potassium concentration >5.5mmol/L was 2.0%, and the incidence of severe hyperkalemia (serum potassium concentration ≥6.0mmol/L) was 0.2%[55]. Guidelines recommending the use of ACE-I do not suggest that renal function or biochemistry be monitored routinely following ACE-I initiation[49,56]. Indeed, for patients with moderately impaired renal function, there is strong evidence that ACE-I are renoprotective, and so have been recommended for use among these patients by the US National Kidney Foundation[56].

Most recent data suggest that patients with prostate cancer exhibit systolic blood pressure above guideline-recommended targets in 75% of cases[38].

Smoking and CV Risk

There is a large body of epidemiologic data to suggest that smoking causes CVD, and that its cessation can lower CV risk[57,58]. A recent individual participant meta-analysis of 503,905 people aged ≥60 years, including 37,952 CV deaths, has shown that former smoking is associated with a hazard ratio of 1.37 (95% CI 1.25-1.49) for CV death compared with never smoking, and current smoking is associated with a hazard ratio of 2.07 (95% CI 1.82-2.36) for CV death compared with never smoking[59]. This study also demonstrated that the excess risk of CV death falls progressively with the duration of quitting smoking.

Physician advice alone on smoking cessation has been shown in one Cochrane review of randomized trials to improve the quit rate among smokers[60]. In this study, even brief advice was associated with a relative risk of quitting of 1.66 (95% CI 1.42-1.94). Furthermore, focusing physicians on patients' smoking habits has been shown to improve quitting rates significantly[61]. In this trial, physicians were randomized to a group that received advice on how to assist patients quit smoking alone (control group) or to have stickers on patients' charts reminding the physician to address the subject of quitting with the patient (reminder group). At six months, 1.3% and 7.0% of the control and reminder group respectively had quit, and at one year, 2.7% and 15% of the control and reminder group respectively had quit (all p-values <0.05 for the pairwise comparisons). Thus, there is direct evidence that a systematic approach to

prompt physicians to address patients' smoking habits is efficacious at increasing quitting rates.

Adiposity and Obesity

There is a positive relationship between obesity, and in particular, central adiposity, and CVD. A recent meta-analysis that included individual-level data from 221,934 subjects in 17 high-income countries, who experienced 14,297 CV events, demonstrated that per standard deviation increase in body mass index and waist circumference, the relative risks (95% confidence interval) of coronary heart disease were respectively 1.11 (1.05-1.17) and 1.12 (1.06-1.19)[62]. For ischemic stroke, the relative risks (95% confidence interval) were respectively 1.06 (0.99-1.13) and 1.14 (1.09-1.20)[62]. Furthermore, in a recent randomized, controlled trial, bariatric surgery ameliorated dysglycemia, a key CV risk factor, in obese individuals[63]. We have recently demonstrated that ADT use is associated with 1.6% increase in body weight at 12 months[64].

Diet and CV Risk

Epidemiologic data indicate that a healthy diet is associated with a lower risk of CVD[65]. In a recent trial in which individuals without CVD but at high CV risk were randomized to a control diet, or a Mediterranean diet supplemented with either extra- virgin olive oil, or nuts, the groups receiving the supplements experienced significant reductions in the risk of myocardial infarction, stroke, or CV death[66]. In the Lyon Diet Heart Study, survivors of a first myocardial infarction were randomized to a Mediterranean diet or a control low-fat diet. The trial was terminated at 27 months when an interim analysis demonstrated a 73% reduction in myocardial infarction or CV death among those randomized to the Mediterranean diet[67].

Physical Activity and CV Risk

According to the Global Burden of Disease estimates, physical inactivity is one of the leading causes of death and disability[68]. Observational evidence suggests that physical activity is associated with lower all-cause and all-cancer mortality[69]. In a meta-epidemiologic study of meta-analyses for the secondary prevention of CVD, exercise interventions were shown to be as effective as drug therapies for the prevention of mortality[70]. In men with PC, an exercise program that consisted of resistance training and cardiovascular exercise, and that included weekly supervision by an exercise physiologist, has been shown to be superior to written exercise advice with respect to objective measurements of physical function and muscle strength[71]. Such an intervention is costly, however, and long-term adherence and effectiveness remain uncertain. Sustainable and cost-effective improvement in physical function and muscle strength may require frequent reminders, and training on exercise techniques that is delivered by electronic media[72].

Alcohol Use and CV Risk

Epidemiologic studies, [73,74], have consistently shown that in populations from high-income countries, with predominantly European ethnic origins, a low level of alcohol intake is associated with a reduction in the risk of ischemic heart disease. Episodes of heavy alcohol use may be a trigger for myocardial infarction[73]. Therefore, heavy alcohol use should be avoided.

Aspirin in the Primary Prevention of CVD

There is long-standing debate with respect to the role of aspirin in the primary prevention of CVD. While there is clear evidence of the protective effect of aspirin in individuals at high CVD risk, aspirin has the potential for adverse effect. The most notable unwanted effect of aspirin is to increase the risk of bleeding. In a meta-analysis of studies from the general United Kingdom population comparing aspirin with controls, aspirin was associated with a risk ratio (95% confidence interval) of gastrointestinal bleeding of 1.88 (1.30-2.74)[75]. When studies using high-dose aspirin are excluded, low-dose aspirin is associated with a 60-70% increase in the risk of gastro-intestinal or major bleeding[75]. In a separate meta-analysis of placebo-controlled trials of aspirin for primary CVD prevention, aspirin used over 330,000 person-years was associated with a trend towards an increase in the risk of hemorrhagic stroke (rate ratio, 95% confidence interval 1.32, 1.00-1.75)[76]. The pooled risk ratio (95% confidence interval) of myocardial infarction, stroke, or vascular death associated with aspirin use was 0.88 (0.82-0.94)[76]. The European Society of Cardiology Working Group on Thrombosis recommends that the antithrombotic benefits of aspirin may outweigh the risk of bleeding when there is ≥2% per year risk of major adverse CV events in individuals without increased bleeding risk[77]. In contrast, other European Society of Cardiology guidelines state that aspirin is not recommended in individuals without CVD due to the increased risk of bleeding[78], while the American College of Chest Physicians recommend that low-dose aspirin be used in all persons aged ≥50 years without symptomatic CVD[79]. Thus, there is uncertainty as to which group (if any) is likely to exhibit benefit in excess of risk with the use of aspirin (or other antiplatelet drugs) for primary CVD prevention.

Recently, the role of aspirin in the primary prevention of CVD has been evaluated in two important trials. In the ASCEND (Effects of Aspirin for Primary Prevention in Persons with Diabetes Mellitus) trial 15,480 adults with diabetes aged ≥45 years were randomly allocated to receive either aspirin 100mg daily or placebo. During a mean 7.4 years' follow-up, 9.6% of the placebo group experienced the primary outcome of myocardial infarction, stroke, transient ischemic attack, or vascular death. Aspirin reduced the risk of a major cardiovascular event (rate ratio 0.88, 95% CI 0.79-0.97), but increased the risk of major bleeding (rate ratio 1.29, 95% CI 1.09-1.52). Consequently, there was no net effect on mortality (rate ratio 0.94, 95% CI 0.85-1.04)[80]. In the ASPREE (Effect of Aspirin on All-Cause Mortality in the Healthy Elderly) trial, 19,114 adults aged ≥70 years with no known cardiovascular disease were randomly allocated to enteric-coated aspirin 100mg daily or placebo. During follow-up, cardiovascular outcomes (including myocardial infarction, stroke, or heart failure

hospitalization) occurred at a rate of 11.3 per 1000 person-years in the placebo group. Aspirin did not reduce cardiovascular events (hazard ratio 0.95, 95% CI 0.83-1.08), but did increase major hemorrhage (hazard ratio 1.38, 95% CI 1.18-1.62). Aspirin use led to an increase in mortality (hazard ratio 1.14, 95% CI 1.01-1.29). This was related to an increase in the risk of cancer death (hazard ratio 1.31, 95% CI 1.10-1.56), and in the risk of developing cancer (981 aspirin participants versus 952 placebo recipients).There was insufficient power to determine whether outcomes were different among different cancer subtypes[81]. A meta-analysis containing pooled the data from older primary prevention trials and these recent new studies demonstrated that the cardiovascular benefits associated with aspirin were modest and negated by major bleeding events[82].Therefore, these new findings do not support the routine use of aspirin for the primary prevention of cardiovascular disease.

Summary

A number of healthcare strategies have been shown to reduce adverse CV outcomes in the general population in primary prevention settings. These include the use of statins[83], and ACE-I[50] among those at high CV risk, the treatment of hypertension, the adoption of healthy diets and regular exercise[84]. There is a paucity of evidence to support such approaches in the PC population specifically, as many men with PC may have been excluded from primary prevention CV trials because of their cancer diagnosis. Given the high absolute risk of CVD in the PC population, the potential for clinical benefit from CVD prevention is large but should not be taken for granted because these men have PC as an important competing risk, and the pharmacotherapeutic strategies are not without risk of adverse effects.

3.0 OBJECTIVES – RADICAL PC1

3.1 Co-Primary Objectives

1. To determine in an unbiased, contemporary sample, the prevalence of cardiovascular risk factors and disease, and the incidence of MACE in men with PC and in particular men treated with ADT.
2. To measure the relationship of ADT with MACE after adjustment for possible confounding factors
3. To identify factors (including clinical factors and PC treatments) that are independently associated with the development of cardiovascular disease in men with PC, and in particular in men treated with ADT.

3.2 Secondary Objectives

1. To determine in an unbiased, contemporary sample, the prevalence of, and the incidence of the following in men with PC and in particular men treated with ADT:
    a. frailty;

   b. decline in cognitive function;
   c. decline in physical function
   d. depressed mood
2. To characterize in an unbiased, contemporary sample of men with PC the relationship between ADT and
   a. frailty;
   b. decline in cognitive function;
   c. decline in physical function
   d. depressed mood
3. To identify factors in men with PC (including clinical factors and PC treatments) that are independently associated with the development of
   a. frailty;
   b. decline in cognitive function;
   c. decline in physical function
   d. depressed mood

4.0 OBJECTIVES – RADICAL PC2

4.1 Primary Objective

To determine whether the routine referral of men within one year of their first diagnosis of PC, or who are commencing ADT for the first time, to a cardiologist or internist, who will implement a systematic CV and lifestyle risk factor modification strategy reduces cardiovascular adverse outcomes and improves cardiovascular risk factor control.

4.2 Secondary Objective

1. To determine whether the routine referral of men within one year of their first diagnosis of PC, or who are commencing ADT for the first time, to a cardiologist or internist, who will implement a systematic CV and lifestyle risk factor modification strategy, as compared with usual care, improves
   a) cognitive function
   b) physical function
2. To estimate the incremental cost-effectiveness ratio of the routine referral of men within one year of their first diagnosis of PC, or who are commencing ADT for the first time, to a cardiologist or internist, who will implement a systematic CV and lifestyle risk factor modification strategy.

5.0 SPECIFIC HYPOTHESES – RADICAL PC1

5.1 Primary Hypothesis

The primary hypothesis is that ADT is independently associated with MACE.

5.2 Secondary Hypotheses

The secondary hypotheses are that in men with PC, ADT use is independently associated with:

1. decline in cognitive function, as measured by the DSS
2. decline in handgrip strength
3. decline in timed get-up-and-go score
4. decline in six-minute walk distance
5. depressed mood, as measured using the PHQ-9 questionnaire
6. decline in function, as measured by the SAGE questionnaire
7. increase in serum LDL concentration
8. increase in Hemoglobin A1c concentration

6.0 HYPOTHESES – RADICAL PC2

6.1 Primary Hypothesis

That the routine referral of men within one year of their first diagnosis of PC, or who are commencing ADT for the first time, to a cardiologist or internist, who will implement a systematic CV and lifestyle risk factor modification strategy reduces the risk of MACE and improves cardiovascular risk factor control.

6.2 Secondary Hypotheses

That the routine referral of men within one year of their first diagnosis of PC, or who are commencing ADT for the first time, to a cardiologist or internist, who will implement a systematic CV and lifestyle risk factor modification strategy, will lead to reduced:

1. risk of MACE
2. systolic blood pressure
3. serum cholesterol concentration

6.3. Tertiary Hypotheses

In men with a new diagnosis of PC or who are commencing ADT for the first time:

1. That a systematic CV and lifestyle risk factor modification strategy:
    a) reduces hemoglobin A1c concentration
    b) increases handgrip strength
    c) reduces get-up-and-go time
    d) reduces waist circumference
    e) increases six-minute walk distance
    f) slows cognitive decline as measured by the DSS test

2. That a systematic CV risk factor modification (including aspirin and statin) is associated with a reduction in PC progression

7.0 STUDY DESIGN – RADICAL PC 1

This prospective cohort study will include men who fulfill any of the following criteria: a) diagnosed with PC within the previous year, or b) men with PC who have commenced ADT for the first time within six months of their enrollment visit, or c) men with PC who will commence ADT for the first time within the month following their enrollment visit. Screening will be performed to ascertain patient eligibility. Patients who have not been diagnosed with PC within the previous year, are only eligible if commencing ADT for the first time. These patients should be recruited prior to the commencement of ADT, however patients will be eligible if they have commenced ADT for the first time within the past six months.

Patients who meet eligibility criteria and who provide informed consent will undergo a baseline visit, at which demographic characteristics, CVD and PC risk factors, past medical history, medications, PC characteristics, physical characteristics, results of routine clinical blood tests, and urinalysis results are recorded. Questionnaires to evaluate dietary habits (FFQ), physical activity levels (PAQ), general function (SAGE, ECOG), cognitive function (DSS), erectile function (IIEF5) and mood (PHQ-9) will be administered at this visit. Consent will be sought to record participants' healthcare card numbers for future linkage with administrative databases where permissible.

Follow-up visits will be conducted annually, and at study close-out. A study close-out date will be announced that will provide at least six months' notice. The study close-out visit will occur as close as practicable to the study close-out date. Data to be collected at each study visit are indicated in Table 3.

Participants will be permitted to post the completed IIEF5 questionnaire in private to avoid any embarrassment associated with providing responses face-to-face with study personnel.

8.0 STUDY DESIGN – RADICAL PC2

This pragmatic[85], prospective, open-label, randomized, controlled trial will include men fulfilling any of the following criteria: a) a new diagnosis of PC (within the previous year), or b) men with PC who have commenced ADT for the first time within six months of their enrolment visit, or c) men with PC who will commence ADT for the first time within the month following their enrolment visit. Screening will be performed to ascertain patient eligibility. Patients who have not been diagnosed with PC within the previous year, are only eligible if commencing ADT for the first time. Patients recruited because they are commencing ADT for the first time should be recruited prior to the commencement of ADT; however, patients will be eligible if they have commenced ADT for the first time within the past one month.

Patients who meet the eligibility criteria and who provide informed consent will be randomized to intervention or control arms via an Interactive Web Randomization System (IWRS). The IWRS is a 24-hour computerized randomization internet system maintained by the coordinating centre at the Population Health Research Institute (PHRI), which is part of Hamilton Health Sciences and McMaster University in Hamilton, Ontario, Canada. The randomization process will use block randomization stratified by centre and plan to prescribe ADT. Study centre personnel will not know the block size.

The intervention will consist of referral of the participant to a cardiologist or internist who will administer a systematic CV and lifestyle risk factor modification strategy, including dietary and exercise advice, advice to quit smoking, and the prescription of statins, ACE-I, and/or other antihypertensive medications where appropriate, and as indicated in the Study Procedures. Participants in the intervention arm will be encouraged to see the cardiologist/internist at least annually.

The control arm will receive usual clinical care. Usual care may include referral to a cardiologist or internist, or the use of treatments included in the intervention as clinically indicated if part of the treating physician's standard practice. The management of cardiovascular risk factors and disease in control arm participants is otherwise guided by their usual healthcare providers according to local standards and guidelines.

Patients who meet eligibility criteria and who provide informed consent will undergo a baseline visit, at which demographic characteristics, CVD and PC risk factors, past medical history, medications, PC characteristics, physical characteristics, results of routine clinical blood tests, and urinalysis results are recorded. Questionnaires to evaluate dietary habits (FFQ), physical activity levels (PAQ), general function (SAGE, ECOG), cognitive function (DSS), erectile function (IIEF5) and mood (PHQ-9) will be administered at this visit. Consent will be sought to record participants' healthcare card numbers for future linkage with administrative databases where permissible.

Follow-up visits will be conducted annually and at study close-out. A study close-out date will be announced that will provide at least six months' notice. The study close-out visit will occur as close as practicable to the study close-out date. At each face-to-face study visit, medication use, physical characteristics, the results of routine clinical blood tests, and clinical outcome events will be recorded. At the 24-month visit, the PAQ, SAGE, ECOG, DSS, IIEF5 and PHQ-9 questionnaires will be re-administered.

Participants will be permitted to post the completed IIEF5 questionnaire in private to avoid any embarrassment associated with providing responses face-to-face with study personnel.

9.0 ENDPOINTS FOR RADICAL PC1

9.1 Primary Endpoint

The primary endpoint is the time to the first occurrence of cardiovascular death, myocardial infarction, stroke, heart failure, or arterial revascularization.

9.2 Secondary Endpoints

The secondary endpoints are:

1. The time to the first occurrence of the composite of all-cause mortality, myocardial infarction, stroke, or heart failure
2. The time to first occurrence of cardiovascular death, myocardial infarction, or stroke
3. The time to first occurrence of cardiovascular death, myocardial infarction, stroke, heart failure, arterial revascularization, or unstable, new or worsening angina,
4. Time to cardiovascular death
5. Time to myocardial infarction
6. Time to stroke
7. Time to heart failure
8. Time to venous thromboembolism

9.3 Tertiary Endpoints

The tertiary endpoints are:

1. Time to new or worsening angina
2. Time to new atrial fibrillation
3. Cognitive function, as measured by the digit symbol substitution test
4. Handgrip strength
5. Timed get-up-and-go test
6. Six-minute walk distance
7. Physical frailty, as measured by a phenotype described by Fried *et al*.[29]
8. Waist circumference
9. HbA1c concentration
10. Lipid profile
11. Time to PC death
12. Time to Distant PC progression
13. The development of castrate-resistant PC
14. PSA progression
15. Biochemical failure

10.0 EFFECTIVENESS ENDPOINTS FOR RADICAL PC2

10.1 Powered First Primary Endpoint

The powered first primary effectiveness endpoint is the the hierarchical composite of cardiovascular death, myocardial infarction, stroke, heart failure, cholesterol control (defined as total cholesterol ≤4mmol/L) and systolic blood pressure control (defined as systolic blood pressure ≤130mmHg).

10.2 Second Primary Endpoint

The second primary effectiveness endpoint is the time to the first occurrence of the composite of cardiovascular death, myocardial infarction, stroke, heart failure.

10.3 Exploratory analyses

The endpoints are systolic blood pressure, total and low-density lipoprotein cholesterol, hemoglobin A1c concentration, handgrip strength, get-up-and-go time, waist circumference, six-minute walk distance and the DSS test

11.0 SAFETY OUTCOMES

The safety outcomes are:

<u>Major Bleeding</u>

1. Major bleeding as defined by modified International Society of Thrombosis and Hemostasis criteria as:
    a. Fatal bleeding and/or
    b. Symptomatic bleeding in a critical area or organ, such as intracranial, intraspinal, intraocular, retroperitoneal, intraarticular or pericardial, or intramuscular with compartment syndrome, or bleeding into the surgical site requiring re-operation and/or
    c. Bleeding leading to hospitalization.

<u>Myositis</u>

2. Myositis as defined by muscle symptoms in association with a serum creatine kinase concentration >10 times the upper limit of normal.[86]

<u>Liver Injury</u>

3. Liver injury, as defined as[87] elevation in alanine transaminase, together with one of:
    a. Hepatomegaly
    b. Jaundice
    c. Elevated direct bilirubin concentration
    d. Increased prothrombin time

Kidney Injury

4. Kidney injury, as defined by[88] a 2-fold or greater increase in serum creatinine concentration from the most recent serum creatinine concentration, or a decrease in estimated glomerular filtration rate of more than 50% from the most recent estimated glomerular filtration rate

Serious Adverse Event (SAE)

According to ICH E6 definitions, a serious adverse event (SAE) is considered any untoward medical occurrence that results in death, is life-threatening, requires in-patient hospitalization (defined as an overnight stay in hospital) or prolongation of existing hospitalization, results in persistent or significant disability/incapacity, is a congenital anomaly/birth defect or is another medically serious event representing a significant hazard comparable to the aforementioned criteria. In RADICAL PC, the efficacy and safety outcomes will be documented but will not require expedited reporting as SAEs. In addition, given that statins and ACE-I have been approved for use for years, with large amounts of data from both randomized trials and other studies on their adverse effect profiles, events that are considered frequent in this population will be exempted from expedited reporting (e.g. planned hospitalizations, pneumonia). All non-exempted SAEs must be reported to PHRI within 24 hours of the site becoming aware of their occurrence.

12.0 INTERVENTIONS IN RADICAL PC2

The intervention group will be referred to a cardiologist or internist who will administer the following:

1. Standardized advice on healthy diet and exercise (consult and written information)
2. Advice to quit smoking, and on available aids to quitting, if they are current smokers
3. Low- to moderate-dose statin (e.g. simvastatin 10-40mg daily, atorvastatin 10-40mg daily, rosuvastatin 5-20mg daily, pravastatin 10-40mg daily)
4. For baseline systolic blood pressure >130mmHg, blood pressure-lowering medication. An ACE-I is preferred if not presently taking an ACE-I or an angiotensin receptor blocker, and if there is no indication for a different blood pressure-lowering medication. For those participants with a systolic blood pressure >130mmHg who are already taking the maximum dose of an ACE-I or an angiotensin receptor blocker, other blood pressure-lowering medications should be prescribed. The choice of any additional blood pressure-lowering medications will be decided by the investigator, taking participant co-morbidities and circumstances into consideration.

The administration of the intervention will be undertaken by a cardiologist or specialist in internal medicine, or their suitably qualified delegate. In previous versions of the study protocol, low-dose antiplatelet was to be prescribed to all participants in the intervention arm of RADICAL PC2. The subsequent findings of the ASPREE and ASCEND trials suggest that low-dose aspirin for the primary

prevention of CVD may have a lower likelihood of net clinical benefit than had been previously anticipated. Therefore, in this version of the protocol, the prescription of low-dose antiplatelet will be left to the discretion of the treating cardiologist/internist.

Sections 10.1-10.3 and 15 of the protocol describe in greater detail the treatments that will be used in the intervention group.

12.1 Cardiovascular Medications

All medications will be open-label. The dose of statin and blood pressure-lowering drug will be titrated if clinically indicated by the investigator or other physician. The target blood pressure will be ≤130mmHg systolic. There is no specific target cholesterol concentration[83]; however, a target LDL concentration <2mmol/L will be encouraged in intervention participants. If the participant has a specific contra-indication to a study drug according to the product monograph, standard of care or the clinical judgment of the investigators, that study drug will not be prescribed. Participants with a contra-indication to a particular study drug may continue to take the other study drugs if there are no contra-indications to the other study drugs. Specific guidance on the initiation and titration of individual study medications is provided in section 15.

12.2 Diet

Participants in the intervention group will be encouraged to adhere to a Mediterranean diet supplemented with olive oil or nuts based on the intervention diet prescribed in the PREDIMED study[66]. Specifically, participants in the intervention arm will be encouraged to consume:

1. ≥4 tablespoons of extra virgin olive oil per day
2. ≥3 servings of tree nuts or peanuts per week (1 serving comprises 30g of a mixture of peanuts, walnuts, almonds, or hazelnuts)
3. 2 servings of fresh fruit per day
4. 3 servings of fresh or cooked vegetables per day (not fried vegetables)
5. 3 servings of fish (especially fatty fish) or seafood per week
6. ≥3 servings of legumes per week
7. white meat instead of red meat
8. for habitual drinkers, wine with meals (and no more than 3 standard drinks per day)
9. avoid soda drinks and soft drinks
10. ≤1 serving per week of commercial bakery goods, sweets, or pastries
11. avoid processed meat
12. <1 serving per week of butter or margarine

12.3 Exercise

Participants in the intervention group will be instructed to undertake a specific exercise program that comprises of at minimum four sessions per week, each lasting 40 minutes. Each session will include 30 minutes of moderate or vigorous aerobic exercise, and approximately 10 minutes of strength training. These prescriptions are consistent with the Physical Activity Guidelines from the Canadian Society for Exercise Physiology for adults (18-64 years old) and older adults (≥65 years old), which indicate accumulating "at least 150 minute of moderate- to vigorous-intensity aerobic physical activity per week, in bouts of 10 minutes or more" to achieve health benefits. These Guidelines also state that it is "beneficial to add muscle and bone strengthening activities using major muscle groups, at least 2 days per week".

Aerobic Exercise:
Participants will be able to choose any mode of exercise that they wish, which may include walking, biking, swimming, pool walking, skiing. We will instruct participants on using the Borg Scale, which was used during the six-minute walk test, to assess moderate- to vigorous- intensity exercise.

Strength Training Exercise:
Each strength training session will focus on major muscle groups and will alternate between: a) lower limbs and core (abdominal and back muscles), and b) upper limbs and core (using different exercises). Printed materials will be provided to: 1) list different exercises for each major muscle group, 2) explain and demonstrate with diagrams how to perform these exercises safely (i.e. form, number of repetitions, etc.), and 3) explain how and when to safely increase the level of difficulty.

13.0 ELIGIBILITY CRITERIA

The following are eligibility criteria for both RADICAL PC1 and RADICAL PC2.

13.1 Inclusion Criteria

A man with a diagnosis of PC that is either

a) new (i.e. the diagnosis was made within 1 year of the enrolment visit), or
b) treated with ADT for the first time within 6 months prior to the enrolment visit, or
c) to be treated with ADT for the first time within 1 month after the enrolment visit

13.2 Exclusion Criteria

Patients will be excluded if they fulfil any of the following:

a) are unwilling to provide consent, or

b) are <45 years of age, or
c) prostate cancer was found incidentally following cystectomy for bladder cancer

Patients will be eligible for RADICAL PC1, but will not be eligible for RADICAL PC2 if they:
a) see a cardiologist every year, or
b) both take a statin and have systolic blood pressure ≤130mmHg

14.0 PATIENT SCREENING AND RECRUITMENT

Patient screening will occur in (but may not be limited to) radiation oncology clinics, medical oncology clinics, urology clinics, and prostate biopsy lists. Each site's approach to screening should ensure that an unbiased and representative population of men with prostate cancer are screened. Ideally all prostate cancer patients at each site should be screened, however we acknowledge that this may not be feasible for reasons of resource limitation. Under such circumstances, random clinics might be selected, and all men with prostate cancer in each of these clinics screened for eligibility. Research personnel will approach potentially eligible patients to obtain written informed consent. RADICAL PC will enrol patients from at least fifteen sites. Eligible patients will be invited to participate in RADICAL PC1, or in RADICAL PC2.

14.1 Participant identification

Each participant will receive a unique identification number which will represent both the assigned study center number and subject number. The center number will be pre-assigned by PHRI. The subject identification number, consisting of center number and subject number must be entered in the PHRI randomization system to obtain the assignment of study treatment. Once assigned to a subject, the subject identification number will not be re-used.

15.0 STUDY PROCEDURES

For participants who provide informed consent to be enrolled in RADICAL PC2, the investigator or delegate will access the PHRI randomization system to confirm eligibility and to obtain the randomized treatment allocation. Randomization will be stratified by the intent to prescribe ADT. Table 3 displays study procedures that will take place at each visit for RADICAL PC1 participants and Table 4 displays study procedures that will take place at each visit for RADICAL PC2 participants. Follow up visits will be completed ± 1 month of the scheduled study visit. Details of the required blood test results are provided in Table 5.

Table 3. Study procedures for RADICAL PC1 participants

Study procedure	Baseline visit	1-year visit	2-year visit	Yearly visit*	Close-out visit
Past medical history	X				
Medications	X		X	X	X
Vital signs†	X	X	X	X*	X
Anthropometrics<br>Handgrip strength<br>Timed get-up-and-go test<br>Six-minute walk test	X		X	X*	X
PC characteristics	X	X	X	X	X
Results of routine blood tests	X	X	X	X	X
FFQ	X		X		
PAQ	X		X	X*	X
ECOG<br>DSS test<br>PHQ-9<br>SAGE<br>IIEF5	X		X	X*	X
Clinical outcome events		X	X	X	X

*Every alternate year's study visit, starting with the 3-year visit, can be conducted by telephone. At telephone visits, physical measurements (i.e. vital signs, anthropometrics, handgrip strength, timed get-up-and-go test and six-minute walk test) and medications, the PAQ, ECOG, DSS test, PHQ-9, SAGE and IIEF will not be collected. However, physical measurements (i.e. vital signs, anthropometrics, handgrip strength, timed get-up-and-go test and six-minute walk test) and medications and the PAQ, ECOG, DSS test, PHQ-9, SAGE and IIEF will be collected at in-person visits.

†When an in-person visit is not possible (e.g. owing to restrictions related to the COVID-19 pandemic), blood pressure and pulse rate measurements recorded by a home monitor or from a clinical visit to a physician or hospital are acceptable. Where multiple such measurements are available, the average value will be recorded for the corresponding visit.

In addition, important co-variates for analyses, including participant access to primary healthcare, vasomotor symptoms and gender identity will be recorded.

Table 4. Study procedures for RADICAL PC2 participants

Study procedure	Baseline visit	3-month phone	6-month phone	12-month visit	18-month phone	24-month visit	Yearly visit	Close-out visit
Past medical history	X							
Medications	X	X	X	X	X	X	X	X
Vital signs#	X			X		X	X	X
Anthropometrics<br>Handgrip strength<br>Timed get-up-and-go test<br>Six-minute walk test	X			X		X	X^	X
PC characteristics	X	X	X	X	X	X	X	X
Results of routine blood tests	X			X		X	X	X
EQ-5D	X			X		X		X
FFQ	X		(subset)†			X		X
PAQ	X		X	X		X	X	X
ECOG<br>DSS test<br>PHQ-9<br>SAGE<br>IIEF5	X					X	X^	X
Clinical outcome events		X	X	X	X	X	X	X
Drug adverse effects		X	X	X	X	X	X	X
Adherence*		X	X	X	X	X	X	X

^ anthropometrics, handgrip strength, timed get-up-and-go test and six-minute walk test and the ECOG, DSS test, PHQ-9, SAGE and IIEF will be collected every alternate year

*for participants in the intervention arm of RADICAL PC2 only

†The FFQ will be performed at the 6-month visit in a subset of participants to allow FFQ reproducibility to be evaluated

# When an in-person visit is not possible (e.g. owing to restrictions related to the COVID-19 pandemic), blood pressure and pulse rate measurements recorded by a home monitor or from a clinical visit to a physician or hospital are acceptable. Where multiple such measurements are available, the average value will be recorded for the corresponding visit.

In addition, important co-variates for analysis, including access to primary healthcare, vascomotor symptoms and gender identity will be recorded.

DSS = digital symbol substitution; ECOG = Eastern Cooperative Oncology Group performance status; FFQ = food frequency questionnaire; IIEF5 = simplified International Index of Erectile Function; PAQ = physical activity questionnaire; PHQ-9 = Patient Health Questionnaire-9; SAGE = Standard Assessment of Global Activities in the Elderly

Table 5. Laboratory Results

Results of the following blood tests ordered by any physician including but not limited to; principal investigator, co-investigator or delegate. Missing laboratory results will be collected from any source accepted including electronic medical record or family physician records.

Laboratory test	Baseline visit	Annual visit	Close-out visit
CBC	X	X †	X
Creatinine	X	X	X
Potassium	X	* †	*
Lipids	X	X	X
HbA1c	X	X	X
CK	X	*	*
Albumin	X	*	*
Testosterone	X	*	*
PSA	X	X	X
Liver function	X	X †	X
Other	#	#	#

*These tests will only be collected if measured for clinical purposes

#Select results of other laboratory tests performed for clinical purposes may be collected under the direction of the study Steering Committee

†These test results will only be collected every second year in RADICAL PC1 participants but will be collected annually in RADICAL PC2 participants

15.1 Baseline Visit

The participant's healthcare card number will be requested where permissible, to permit future linkage to administrative databases, to allow identification and verification of outcome events. The following participant characteristics will be recorded at the baseline visit:

Characteristic class	Parameter
Demographics	Date of birth<br>Healthcare card number (where permissible)<br>Ethnicity

	Education<br>Employment
Risk factors	Tobacco use<br>Past history of coronary artery disease<br>Past history of stroke<br>Diabetes mellitus<br>Hypertension<br>Physical activity<br><br>Dietary habits<br>Alcohol use
Past history	Co-morbid medical conditions
Current medication use	Antiplatelet<br>Anticoagulant<br>Statin<br>ACE-I/ARB<br>Beta-blocker<br>Diabetes medications<br>Medications used in the treatment of prostate cancer
Routine blood test results *(Note: values within 12 months prior to the baseline visit are acceptable provided there has been no major intercurrent illness. If there has been a major intercurrent illness, these blood tests should be performed after the intercurrent illness has resolved and within one month of the baseline visit)*	Complete blood examination<br>Serum biochemistry<br>• Potassium concentration<br>• Creatinine concentration<br>• Creatine kinase (CK)<br>Total cholesterol<br>Low-density lipoprotein<br>High-density lipoprotein<br>Triglyceride<br>Liver function tests<br>• Alanine transaminase (ALT)<br>• Gamma glutamyl transaminase (GGT)<br>• Alkaline phosphatase (ALP)<br>• Bilirubin<br>• Albumin<br>Hemoglobin A1c<br>Prostate specific antigen<br>Testosterone<br>Follicle stimulating hormone will be collected if requested by the participant's physician as part of routine clinical care but otherwise will not be collected
Prostate cancer characteristics	Prostate Cancer Treatment
Anthropometrics	Height<br>Weight<br>Waist circumference<br>Hip circumference
Physical	Resting heart rate<br>Resting blood pressure<br>Handgrip strength by Jamar dynamometer<br>Timed get-up-and-go test<br>Six-minute walk test
Diet	Food frequency questionnaire
Cognitive Function	DSS
General Function	ECOG performance status<br>SAGE

Erectile function	IIEF5
Psychological Function	PHQ-9
Physical activity	PAQ
Health-related quality-of-life	EQ-5D (RADICAL PC2 only)

15.2 Subsequent Visits

Refer to Tables 3 and 4.

Special Telephone Visits for RADICAL PC2 Participants

Telephone visits will occur at 3±1, 6±1, and 18±1 months for RADICAL PC2 (and not RADICAL PC1) participants. The following will be recorded at the telephone visits.

Current medication use	Antiplatelet<br>Anticoagulant<br>Statin<br>ACE-I/ARB<br>Beta-blocker<br>Other medications – to be individually listed<br>Androgen deprivation therapy
Clinical Events / Interventions	Refer to outcome event definitions
Prostate Cancer Characteristics	New treatments<br>Metastasis<br>Change in clinical stage
Drug adherence	Adherence questions (RADICAL PC2 only)
Physical activity	PAQ

15.3 Additional Visits

The study Principal Investigator or delegate will see the patient at each scheduled visit; will clinically assess the patient and will review the results of recent blood work. The study Principal Investigator or delegate will use his/her clinical judgement based on standard of care, to determine if more frequent testing or additional visits are needed. Additional visits with a cardiologist or specialist in internal medicine, or their suitably qualified delegate are encouraged if needed for the management of cardiovascular risk factors in participants randomized to the intervention arm in RADICAL PC2.

The study Principal Investigator or delegate will inform the patient's family physician, as well as the cardiologist/internal medicine specialist or other subspecialties about the patients' participation in the trial, as required, including the treatments the patient is being prescribed.

16.0 STUDY INTERVENTION ADHERENCE

The investigator should encourage participants to take any prescribed study drug as prescribed. The participant should contact the investigator or delegate if unable to take study drugs as prescribed for any reason. The investigator or delegate will also encourage the participant to adhere to prescribed diet and exercise regimens at every study visit, including telephone visits.

16.1 Discontinuation of study treatment

All participants will be encouraged to remain on treatment and under observation for the full duration of the study. However, at any time during the study, subjects may withdraw from the study at their own request or at the request of their legally acceptable representative. The subject will not suffer any disadvantage as a result. In all cases, the reason for discontinuation of study treatments (including “at the subject’s request”) must be recorded on the case report form (CRF) and in the participant’s medical records.

It is important to note that discontinuation from the study should only be undertaken with close consultation with a physician. Additionally, withdrawal of consent does not withdraw permission to collect vital status. Withdrawal of this consent must be made separately. In cases where subjects indicate they do not want to “continue”, investigators must determine whether this refers to discontinuation of study treatment (the most common expected scenario), unwillingness to attend follow-up visits, unwillingness to have telephone contact, unwillingness to have any contact with study personnel, or unwillingness to allow contact with a third party (e.g., family member or doctor). In all cases, every effort must be made to continue to follow the participant at regular study visits. Survival status and outcome information must be determined for all subjects.

16.2 Post-study Treatment for RADICAL PC2

At the study close-out visit for RADICAL PC2, for participants who were randomized to the intervention arm, the investigator will individualize the use of study treatments with each participant. The decision to continue with study treatments will be determined by clinical factors including (but not limited to) participant blood pressure, cholesterol concentrations, and the occurrence of MACE.

17.0 GUIDANCE FOR THE IMPLEMENTATION OF STUDY INTERVENTIONS

The following recommendations are intended only as guidance and should not replace clinical judgement. The highest priority is participant safety, which should be taken into consideration by the investigator when they prescribe an intervention.

17.1 Guidance for the initiation of study drug in the intervention arm of RADICAL PC2

The target systolic blood pressure in the intervention group in RADICAL PC2 is ≤130mmHg. Multiple blood pressure-lowering drugs may be needed to achieve this target. Titration of blood pressure-lowering medications should occur to aim to achieve the target systolic blood pressure of ≤130mmHg. Additional visits (i.e. in addition to those required by the study protocol) may be needed and are acceptable to accomplish appropriate titration of blood pressure-lowering drugs. The initial choice of blood pressure-lowering medication is at the discretion of the prescribing physician and should be individualized according to the participant's co-morbidities and medical history. Where there are no specific indications for choosing a particular blood pressure-lowering medication, ACE-I will be preferred. Participants should not have study ACE-I prescribed if they are already taking an ACE-I or an angiotensin receptor blocker. Participants randomized to the intervention arm in RADICAL PC2 should have study drugs initiated at a low dose to maximize drug tolerability, and to minimize the risk of drug discontinuation due to dose-related effects, such as hypotension. The following are examples of recommended drug initiation and escalation strategies:

<u>ACE-I</u>

- ramipril commence at 2.5mg once daily, increase to 5mg once daily at 3 month visit if the systolic blood pressure >130mmHg and there are no adverse effects, and increase to 10mg once daily at 6 month telephone visit if the systolic blood pressure >130mmHg and there are no adverse effects
- perindopril commence at 2mg once daily, increase to 4mg once daily at 3 month visit if the systolic blood pressure >130mmHg and there are no adverse effects, and increase to 8mg once daily at 6 month telephone visit if the systolic blood pressure >130mmHg and there are no adverse effects

<u>Statin</u>

- simvastatin commence at 20mg once daily, increase to 40mg once daily at 3 month visit if there are no adverse effects
- atorvastatin commence at 20mg once daily, increase to 40mg once daily at 3 month visit if there are no adverse effects
- rosuvastatin commence at 10mg once daily, increase to 20mg once daily at 3 month visit if there are no adverse effects

17.2 Guidance for participants who develop an indication for study medication in the intervention or control group

If a participant in either the intervention or control group develops an indication for a study medication (e.g. myocardial infarction), then they should be prescribed these medications per routine clinical practice. Care should be taken to ensure participants randomized to the intervention arm of RADICAL PC2 do

not receive double dosing of cardiovascular drugs such as cholesterol-lowering drugs, or ACE-I (i.e. they should only receive a single prescription for each medication).

17.3 Guidance for participants who require an invasive procedure

If a participant requires an invasive procedure, study antiplatelet and blood pressure-lowering medications can be withheld at the discretion of the investigator. For many elective invasive procedures, study antiplatelet should be withheld for at least 3 days beforehand, and resumed on day 8 post-procedure and when felt clinically safe to do so[89].

17.4 Guidance for the development of muscle symptoms on statins

At least two studies have reported on the successful use and tolerability of an alternative statin in patients who were not able to tolerate a first statin[90,91]. Therefore, in the absence of myositis (see Outcome Event Definitions below), the lowest possible dose of alternative statins should be considered for participants who develop muscle pain or weakness on a statin.

17.5 Guidance for the use of statins in the presence of abnormal liver function

Chronic liver disease and compensated cirrhosis are not considered contra-indications for statin therapy, whereas decompensated cirrhosis or acute liver failure are contra-indications to statin therapy[87]. For patients taking statins, if elevated alanine transaminase or aspartate transaminase concentrations develop, there is no evidence that statin therapy should be altered or discontinued in the asymptomatic patient unless there is further evidence of hepatic dysfunction, such as hepatomegaly, jaundice, elevated direct bilirubin concentration, or increased prothrombin time[87].

17.6 Guidance for the implementation of lifestyle interventions

Printed material as well as face-to-face counselling should be provided to inform intervention participants of and encourage them to adhere to a program of regular exercise and a healthy diet. The recommendations contained in the printed material can be tailored as appropriate for each participant. For example, vigorous exercise is not advised in the weeks following prostatectomy.

Smoking should be discouraged. Strategies to encourage smoking cessation should be employed as available in each clinical setting. These may include pharmacologic support to smoking cessation and referral to smoking cessation clinics or programs as appropriate.

18.0 STUDY TIMELINE

Date	Activity
April to September 2015	Case report form preparation<br>Database preparation<br>Research ethics committee application
October 2015	Enrol first participant
December 2024 or when target sample size reached	Enrol last participant
December 2025 or 12 months after enrolment of last participant	Last clinical activity<br>Data analysis<br>Knowledge dissemination

19.0 COMMITTEES

The Operations Committee will be responsible for ensuring that study execution and management are of the highest quality. This committee will determine its own guidelines and approve the criteria and guidelines of the other committees. The Operations Committee will convene monthly to discuss and report on the ongoing supervision of the study. The Operations Committee will consist of the study principal investigators, senior study coordinator and program manager.

The Steering Committee will comprise study principal investigators, and additional scientists with clinical and methodological expertise. The Steering Committee will be responsible for producing and conducting a scientifically sound study design and ensuring accurate reporting of the study. The steering committee will meet periodically to address and resolve scientific and practical issues encountered during the study.

The Events Committee, consisting of members with clinical and methodological expertise relevant to the study, will oversee the process of event verification.

An independent DSMB will be formed, comprising a chair, co-chair, and members who have recognized expertise in clinical trials, CVD, PC and biostatistics, and who are not members of the Steering Committee or involved in the conduct of the RADICAL PC studies. The DSMB will be responsible for:

- Monitoring efficacy and safety of the studied interventions in RADICAL PC2 based on periodic updates throughout the study
- Give recommendations to the Steering Committee as to whether to continue, modify or stop the RADICAL PC2 study. The DSMB plays a key role in monitoring all aspects of the study. This committee will review aggregate data by treatment group and are tasked with assessing risk and benefit for the study subjects. At the first meeting of the DSMB, the DSMB charter and the frequency of meetings will be agreed upon. The DSMB will be supported at meetings by the designated PHRI biostatistician. The

principal investigators will attend the open portion of the meeting to provide reports and respond to questions concerning the study.

20.0 STUDY COORDINATION AND EXECUTION

The study will be coordinated by the Population Health Research Institute (PHRI) of McMaster University, Canada. The role of PHRI will be to facilitate and oversee the execution of the study. PHRI will keep the Steering Committee informed of the progress and the conduct of the study, and will provide methodological and administrative support to the DSMB, and will prepare appropriate study documentation and reports for these committees.

The Site Investigators will be responsible for the conduct of the study in their respective centres. Each site will have at least one Site Principal Investigator.

21.0 DETERMINATION OF SAMPLE SIZE

21.1 RADICAL PC1

For the observational analyses, a sample size of 5000 will have 86% power to detect a hazard ratio of 1.35 for the primary composite endpoint of time-to-first of cardiovascular death, myocardial infarction, stroke, heart failure or arterial revascularization among ADT recipients as compared with individuals not exposed to ADT.

In addition to 4000 participants recruited in RADICAL PC overall, we will include 1000 participants to be recruited in a separate study funded by the United States Department of Defense. Thus, observational analyses will be based on a total of 5000 participants.

21.2 RADICAL PC2

By running 1000 simulations incorporating the blinded data from the trial as of November 2024, a sample size of 2500 randomised participants in RADICAL PC2 is estimated to have 86% power to identify a win ratio of 1.22 in the intervention arm with 34% of control-intervention pairs tied.

DATA RECORDING

All data will be managed centrally at PHRI, McMaster University and Hamilton Health Sciences, Hamilton, ON, Canada. Case report forms will be completed electronically using iDataFax, or will be collected on paper CRF and faxed to PHRI for integration into iDataFax. All data will be kept secure and the confidentiality of all study participants will be carefully protected.

MONITORING

In accordance with applicable regulations, GCP, and PHRI's procedures, Site Management Coordinators and PHRI project office staff will contact the site prior to the start of the study to review with the site staff the protocol, study requirements, and their responsibilities to satisfy ethical requirements. When reviewing data collection procedures, the discussion will also include identification and documentation of source data items.

PHRI is responsible for the monitoring of the study. The purpose of monitoring is to verify that the data and study procedures have been conducted as described in this protocol and to ensure the validity of the study results. Study monitors will follow the Site Management Coordination Plan to verify that:

1. Data are authentic, accurate and complete
2. Safety and rights of subjects are being protected
3. Study is conducted in accordance with the currently approved protocol (including study treatment being used in accordance with the protocol)
4. Any other study agreements are met. Monitoring will encompass a variety of methods at the central, and site levels; incorporate central data verification, event verification, and local site monitoring. Signed informed consent will be verified to ensure that consent has been obtained. The investigator and the head of the medical institution (where applicable) agree to allow the monitor, auditors, and inspectors direct access to all relevant documents.

ETHICAL CONSIDERATIONS

The procedures set out in this protocol, pertaining to the conduct, evaluation, and documentation of this study, are designed to ensure that the investigator abides by the guiding principles detailed in the Declaration of Helsinki. The study will also be carried out in keeping with applicable local law(s) and regulation(s). Documented approval from appropriate research ethics committee (REC) or institutional review board (IRB) will be obtained for all participating centers before start of the study, according to local laws and regulations. When necessary, an extension, amendment or renewal of the REC/IRB approval must be obtained.

Strict adherence to all specifications laid down in this protocol is required for all aspects of study conduct; the investigator may not modify or alter the procedures described in this protocol.

PARTICIPANT INFORMATION AND CONSENT

All relevant information on the study will be summarized in a participant information sheet and informed consent form provided by the study centre or delegate. A sample subject information and informed consent form is provided as a document separate to this protocol.

Based on this participant information sheet, the investigator or designee will explain all relevant aspects of the study to each subject / legal representative or proxy consenter (if the participant is under legal protection), prior to his entry into the study (i.e. before any examinations, study procedures, or any study-specific data are recorded on study-specific forms).

Each participant / legal representative or proxy consenter will have ample time and opportunity to ask questions and will be informed about the right to withdraw from the study at any time without any disadvantage and without having to provide reasons for this decision.

Only if the participant / legal representative or proxy consenter voluntarily agrees to sign the informed consent form and has done so, may he enter the study. Additionally, the investigator and other information provider (if any) will personally sign and date the form. The participant / legal representative or proxy consenter will receive a copy of the signed and dated form.

The signed informed consent statement is to remain in the investigator site file or, if locally required, in the participant's note/file of the medical institution. Any other handling and storage of the signed informed consent statement will be detailed in the informed consent form.

PUBLICATION POLICY

The key design elements of this protocol will be posted in a publicly accessible database such as clinicaltrials.gov. The study results will be reported irrespective of the outcome of the study. The Operations Committee which will also serve as a Publication Committee and will decide on the authorship of all papers. The main study results will be written by a writing group led by members of the Operations Committee, and may include additional individuals who have made substantial and sustained contributions on behalf of the whole study group.

22.0 STATISTICAL ANALYSIS

General considerations

General description of the statistical methods is outlined below. A more detailed statistical analysis plan (SAP) will be provided in a separate document.

The core SAP document will provide a more technical and detailed elaboration of the principal features of the planned analyses, e.g. censoring schemes for time-to-event variables. The core SAP will be finalized ideally prior to study enrollment, at the latest before any substantial information in the trial has accumulated.

Amendments and/or appendices to the core SAP will provide more details on the coding guidelines, data-handling, and output tables and figures. These SAP

associated documents will be finalized ideally 6 months before planned study end to take into account emerging data external to the study becoming available during conduct of the study that could influence study interpretation. All SAP associated documents will be finalized without knowledge of any emerging results from the study.

While cause of death in RADICAL PC will be attributed to CV causes in instances when the cause of death is unknown (see Outcome Event Definitions), some of these cases may in fact be due to PC. For this reason, sensitivity analyses will be performed using a similar approach to the TROG 96.01 trial[92]. According to this approach, a prostate cancer clinical progression score (PCPS) will be awarded in cases where a participant dies, withdraws consent, or is lost to follow-up (Table). All analyses in RADICAL PC that feature cause-specific mortality will be primarily conducted with the cause of death assigned as adjudicated. The analyses will also be repeated with adjustment for PCPS, and sensitivity analyses will be performed in which participants dying of CV cases who have a PCPS of 9 or 10 will be considered to have died from PC.

<u>Table</u>

PCPS	Description
10	Distant progression and either secondary therapeutic intervention or referral to palliative care
9	Distant progression and no secondary therapeutic intervention
8	Secondary therapeutic intervention and no distant progression
7	No secondary therapeutic intervention and no distant progression
6	PSA progression and secondary therapeutic intervention; no clinical progression
5	High-risk PSA progression without clinical progression or secondary therapeutic intervention
4	Low-risk PSA progression without clinical progression or secondary therapeutic intervention
3	Death on primary treatment
2	In remission (no PSA or clinical progression, and no secondary therapeutic intervention)
1	Alive, but withdrawal of further information, or lost to follow-up

RADICAL PC1

Analysis sets

The analysis set will include all participants who provide informed consent to RADICAL PC1.

Data scope

The data scope will include all outcome events observed from the date of informed consent until the date of the Close-out Visit for each subject. For subjects who are unable to attend the Close-out Visit within the acceptable closeout time-window (range of dates depending on date of announcement of study termination), a calendar date will be chosen after which events will not be counted for primary analysis.

Analysis of the primary hypothesis

The hypothesis that ADT is independently associated with MACE will first be evaluated by competing risks regression[93] with adjustment for the following potential confounders: age, tobacco use, baseline hypertension, baseline diabetes, baseline dyslipidemia, past history of CVD, physical activity level, ECOG performance status. Further adjustment will be made for PC stage (non-metastatic versus metastatic).

Subgroup analyses

Pre-specified subgroup analyses will be performed according to the following subgroups:

Subgroup	Rationale
Age (<65, 65-75, >75 years)	Corresponds with age quartiles of the cohort
PC stage (metastatic, non-metastatic)	Those with non-metastatic disease might stand to benefit less from ADT than those with metastatic disease, so adverse effects of ADT might be less acceptable in this subgroup
Past history of CVD (yes, no)	Prior research suggests that the mortality benefit of ADT might be limited to those without co-morbidities

	such as CVD[94]
Past history of diabetes (yes, no)<br><br>Self-reported hypertension (yes, no)<br><br>Baseline systolic blood pressure ≥140mmHg or diastolic blood pressure ≥90mmHg<br><br>Baseline systolic blood pressure ≥130mmHg or diastolic blood pressure ≥80mmHg<br><br>Baseline use of lipid-lowering medication (yes, no)<br><br>Baseline cholesterol ≤4mmol/L (yes, no)	Prior research suggests that the mortality benefit of ADT might be limited to those without co-morbidities[94]. Blood pressure thresholds and cholesterol thresholds have been chosen based on levels that have historically and are currently used clinically to identify individuals at higher CVD risk.
Grip strength (categorized by tertile)	Grip strength is a powerful prognostic factor[95] and is reduced by ADT. It is plausible that those with lower muscle strength reserves might be more affected by adverse drug effects

Effect modification will be inferred for an exposure-subgroup interaction term p-value <0.05.

RADICAL PC2 Analysis sets and data scopes

<u>Analysis sets</u>

Intention-to-treat analysis set

The intention-to-treat analysis set, also termed full analysis set in the International Conference on Harmonisation (ICH) E9 guideline, will include all randomized participants.

<u>Data scope according to intention-to-treat principle</u>

Analyses according to the intention-to-treat principle will be based on the intention-to-treat analysis set and will include all outcome events observed from randomization until the date of the Close-out Visit for each subject. For subjects

who are unable to attend the Close-out Visit within the acceptable closeout time-window (range of dates depending on date of announcement of study termination), a calendar date will be chosen after which events will not be counted for primary analysis. Subjects will be kept in the study group to which they were randomized and the follow-up period for each subject will be as long and complete as possible.

Additional data scopes for secondary safety analyses

Additional analyses of safety outcomes will be based on the safety analysis set. Subjects will be kept in the study group to which they were randomized. The outcome events will include all outcome events observed from randomization until the date of the Close-out Visit or a chosen calendar date described above for each subject.

Subgroup variables

The homogeneity of treatment effect will be examined for the following subgroups:

Subgroup	Rationale
ADT (yes, no)	If ADT increases cardiovascular risk factors, those treated with ADT might stand to benefit more from the intervention
ADT type	There is evidence of heterogeneity of cardiovascular effects of different types of ADT[96]
Age (<65, 65-75, >75 years)	Corresponds with age quartiles of the cohort
PC stage (metastatic, non-metastatic)	Those with metastatic disease might stand to benefit less from prevention of competing risks than those with non-metastatic disease, owing to higher PC-specific mortality
PC treatment strategy (active surveillance, prostatectomy, radiotherapy, ADT only)	These subgroups represent clinically relevant populations that have implementation implications in the event of heterogeneity of the intervention effect

Past history of CVD (yes, no)	Individuals at high cardiovascular risk may stand to benefit more from cardiovascular risk reduction than those at lower cardiovascular risk
Past history of diabetes (yes, no)<br><br>Self-reported hypertension (yes, no)<br><br>Baseline systolic blood pressure ≥140mmHg or diastolic blood pressure ≥90mmHg<br><br>Baseline systolic blood pressure ≥130mmHg or diastolic blood pressure ≥80mmHg<br><br>Baseline use of lipid-lowering medication (yes, no)<br><br>Baseline cholesterol ≤4mmol/L (yes, no)	Individuals at high cardiovascular risk may stand to benefit more from cardiovascular risk reduction than those at lower cardiovascular risk. Blood pressure thresholds and cholesterol thresholds have been chosen based on levels that have historically and are currently used clinically to identify individuals at higher CVD risk.

Effect modification will be inferred for an intervention-subgroup interaction term p-value <0.05.

Analysis of the (powered) first primary effectiveness endpoint – RADICAL PC2

Analysis of the primary effectiveness endpoint will be based on the intention-to-treat principle and will be conducted according to the win ratio approach. The win ratio is calculated as the number of intervention-control pairs where the intervention is the "winner" as a ratio of the number of intervention-control pairs where the intervention is the "loser". The contribution of individual components of the hierarchical composite endpoint will be evaluated.

Analysis of the (non-powered) second primary effectiveness endpoint – RADICAL PC2

Analysis of the second primary effectiveness endpoint will be based on the intention-to-treat principle. Estimates of cumulative incidence and the cumulative hazard functions will be provided to evaluate the timing of event occurrence and the consistency of any treatment effects at all-time points (i.e. that the two survival curves do not cross). The sub distribution hazard ratio for the

intervention, along with 95% confidence intervals, will be estimated based on competing risks regression[93]. The occurrence of non-cardiovascular death in the absence of the primary effectiveness endpoint will be considered a competing risk.

The first primary effectiveness endpoint will be tested with a type 1 error rate of 5%. For the second primary effectiveness endpoint, to ensure that the overall type-I error rate at the final analysis is maintained, a gatekeeping hierarchical hypothesis testing strategy will be used. The second primary effectiveness endpoint will be tested (at 5% error rate) only if the test of the first primary effectiveness endpoint meets the 5% threshold (p-value<0.05)

Analysis of the exploratory effectiveness endpoints – RADICAL PC2

For analyses of continuous endpoints, linear mixed models will be developed with an interaction term between treatment allocation group and study visit, and participant identity as a random effect. A significant interaction term at $p<0.05$ will indicate a significant difference in the trajectory of these variables over time.

Per protocol analyses of the primary effectiveness endpoints

Exploratory analyses will be undertaken on a per protocol basis. Participants in the intervention arm will be considered "on treatment" until the last time they acknowledge a visit to a cardiologist or internist, at which time they will be censored. Participants in the control arm will be considered "on treatment" until the first time they acknowledge a visit to a cardiologist or internist, at which time they will be censored.

The above section describes the statistical methods for the clinical investigation. Additional details on statistical analyses, including justification of clinical investigation design, sensitivity analyses, poolability analyses, subgroup analyses and analyses of descriptive endpoints, will be described in a separate Statistical Analysis Plan

23.0 CLINICAL EVENT DEFINITIONS

23.1 Myocardial infarction

The MI definition is based on the Third Definition of MI[97] and includes the following:

23.2 Non-procedural MI

Either Cardiac ischemic symptoms (e.g. pain such as pressure, tightness or dyspnea) at rest or ischemic symptoms on minimal exertion, either of which lasts ≥ 20 minutes that the investigator determines is secondary to ischemia,

Or ECG or imaging changes consistent with MI in the absence of conduction abnormalities

New ECG changes indicative of trans-mural infarction

- Q wave in leads V2 and V3 ≥ 0.02 sec or
- QS complex in leads V2 and V3 or
- Q wave ≥0.03 sec and ≥ 0.1 mV deep in leads I, II, aVL, aVF or V4-V6 in any two leads of a contiguous lead grouping [lateral - I and aVL; precordial - V1-V6; infero-posterior - II, III, aVF] or
- QS complex in leads I, II, aVL, aVF or V4-V6 in any two leads of a contiguous lead grouping [lateral - I and aVL; precordial - V1-V6; infero-posterior - II, III, aVF] or
    - R wave ≥ 0.04 sec in V1 and V2 and R/S ≥ 1 with a concordant positive T wave
- New significant ST-segment- T-wave changes in two or more contiguous leads:
    - ST elevation at the J point ≥ 0.1 mV in all leads other than leads V2 and V3 where the following cut points apply: ≥ 0.2 mV in men ≥ 40 years; 0.25 mV in men < 40 years, or
    - ST depression horizontal or down sloping ≥ 0.05 mV, or
    - T wave inversion ≥ 0.1mV with prominent R wave or R/S ratio ≥ 1.
- Development of new left bundle branch block (LBBB)
- Imaging evidence of new loss of viable myocardium or new regional wall motion abnormality
- Identification of an intracoronary thrombus by angiography

And

Elevation of cardiac biomarker values (Troponin preferably, CKMB, AST, LDH or myoglobin) with at least one value above the 99th percentile upper reference limit (URL) of the local laboratory.

NOTE: In the absence of a clinical MI, cardiac troponin may be elevated in several conditions such as heart failure, tachy/brady arrhythmia, aortic dissection, pulmonary embolism, severe respiratory failure, sepsis, renal failure, stroke, subarachnoid hemorrhage, severe anemia, contusion, myocarditis, strenuous exercise, etc.

23.3 Peri-procedural MI

23.3.1 PCI-related MI

Either Cardiac ischemic symptoms as above

Or ECG or imaging changes consistent with MI as above or angiographic findings consistent with a procedural complication,

And troponin concentrations within 48 hours after the procedure that are a) greater than 5 X 99th percentile URL in patients with normal baseline values (less than 99th percentile URL), or b) >20% above baseline concentrations if baseline values are elevated and are stable or falling.

23.3.2 CABG-related MI

ECG, or imaging changes consistent with MI, or angiographically documented new graft or new native coronary artery occlusion, as described in 1.1 above,

And increased cardiac biomarker values (greater than 10X 99th percentile URL) during the first 48 hours following CABG in patients with normal baseline troponin values.

23.2 Cardiovascular Death
Defined as a death for which a definite non-cardiovascular cause (e.g. cancer) has not been identified. Uncertain causes of deaths are presumed to be cardiovascular unless proven otherwise. Cardiovascular deaths include any of the following:

23.2.1 Sudden death
Sudden death includes:

- Witnessed cardiac arrest or cardiovascular collapse, or
- Resuscitated sudden cardiac arrest, from which the participant later dies from the sequelae of the event (later may be hours, days or weeks) or patients who die during an attempted resuscitation, or
- Unwitnessed death occurring unexpectedly (e.g. patient found dead in his/her bed)

If a patient meets criteria for one of the other causes of cardiovascular death defined below, they are not said to have suffered sudden death.

23.2.2 Death due to pump failure/mechanical cardiac causes
Death due to pump failure, including cardiogenic pulmonary edema, cardiogenic shock, and terminal arrhythmias associated with heart failure, cardiac tamponade, valvular heart disease, and ventricular septal rupture.

23.2.3 Death due to other cardiovascular cause

Includes deaths 1) due to hemorrhagic or non-hemorrhagic stroke, or 2) within 30 days of myocardial infarction, or 3) due to pulmonary embolism, or 4) due to documented ventricular arrhythmia, or 5) after invasive cardiovascular intervention or procedure, or 6) other cardiovascular causes not mentioned above.

24.3 Non-cardiovascular death

Non-cardiovascular death is defined as any death for which definite evidence of a primary non-cardiovascular cause exists. Categories of non-cardiovascular death include:

24.3.1 Malignancy death

- Prostate cancer: death may only be attributed to PC in the presence of metastatic disease or unrelieved urinary obstruction, however death in the presence of metastatic PC or PC with urinary obstruction is not necessarily due to the PC (i.e. participants with metastatic PC or PC with urinary obstruction can have the cause of death attributed to a non-PC cause)
- Gastrointestinal malignancy (including pancreatic and liver cancer)
- Lung malignancy
- Breast malignancy
- Brain malignancy
- Skin malignancy
- Hematologic malignancy (e.g. leukemia or lymphoma, multiple myeloma)
- Non-prostate urologic malignancy
- Bone/connective tissue (e.g. sarcoma)
- Unknown primary site
- Other malignancy (specify)

24.3.2 Other non-cardiovascular death not due to malignancy

- Respiratory failure
- Gastrointestinal (non-hemorrhagic – see fatal bleeding – i.e. diverticulitis, perforation, ischemic infarction)
- Liver failure
- Infection / sepsis
- Renal failure
- Accident, trauma
- Suicide
- Bleeding other than hemorrhagic stroke
- Other non-vascular

24.4 Revascularization

- Percutaneous coronary intervention (PCI): angioplasty only or other procedure with or without stent insertion
- Coronary artery bypass graft (CABG) surgery

- Carotid angioplasty with or without stent
- Carotid endarterectomy or carotid bypass
- Other arterial angioplasty with or without stent
- Other arterial surgery: This includes aortic aneurysm repair including stent for aortic aneurysm, aortobifemoral bypass, femoropopliteal bypass, femoral-tibial bypass, cerebral aneurysm surgery, and thoracic aneurysm surgery

24.5 Stroke

Stroke is defined as acute focal or global neurological dysfunction caused by brain or retinal vascular injury due to primary hemorrhage or infarction. Symptoms or signs must persist ≥24 hours or when acute stroke is identified on brain imaging (i.e. if there is an acute/subacute stroke documented by CT or MRI) or at autopsy, the duration of symptoms/signs may be less than 24 hours. If death occurs within 24 hours, the neurological deficit must persist up to the time of death. Stroke can be a clinical diagnosis; while no neuroimaging is required for a diagnosis of stroke, neuroimaging makes the diagnosis more reliable. Stroke does not include diffuse brain ischemia due to anoxic-ischemic encephalopathy, or subdural hematoma.

Key data elements for the diagnosis of stroke (type-unspecified):
1. Was there abrupt onset of focal or global neurological deficit?
2. Did symptoms/sign persist for ≥24 hours?
   a. If not, was there neuro-imaging evidence of acute brain infarction or hemorrhage?
3. Absence of an identified nonvascular cause?

Based on the clinical symptoms or signs, and computerized tomography (CT) or MRI imaging, strokes will be classified as:

- Ischemic stroke
- Hemorrhagic stroke
  - Primary intracerebral / intraparenchymal brain hemorrhage
  - Subarachnoid hemorrhage
- Uncertain or unknown stroke

24.5.1 Ischemic stroke

Stroke with CT or MRI performed within 3 weeks that is either normal or shows infarct in the clinically expected area, or evidence of infarct in autopsy. Secondary hemorrhagic transformation of an ischemic stroke, even if severe, is categorized as ischemic stroke.

24.5.2 Hemorrhagic stroke

Intraparenchymal brain hemorrhage

Stroke with CT/MRI evidence of primary hemorrhage into the brain parenchyma including the brainstem and cerebellum (does not include hemorrhage secondary to ischemic stroke ‘hemorrhagic transformation’, post-traumatic bleeding into an

area of contusion, hemorrhage into a tumour). Primary intraventricular hemorrhage should be included as intracerebral hemorrhage.

Subarachnoid hemorrhage

CT or cerebrospinal fluid evidence of bleeding primarily in the subarachnoid space. Subarachnoid hemorrhage related to traumatic cerebral contusion or skull fracture not be counted as a stroke outcome (i.e. traumatic subarachnoid hemorrhages are not counted as strokes). Large or superficial intracerebral hemorrhages often are associated with minor amounts of subarachnoid hemorrhage, but these should be classified as intraparenchymal hemorrhages.

24.5.3 Uncertain or unknown stroke

Definite stroke that does not meet the above criteria for ischemic or hemorrhagic stroke (e.g. CT scan or MRI not done). The neurological deficit must have been present for 24 hours or more.

24.5.4 Transient ischemic attack (TIA)

Transient ischemic attack is defined as the presence of acute transient focal neurological deficits caused by brain or retinal ischemia without acute infarction, with the deficits lasting less than 24 hours. If brain imaging (CT or MRI shows acute changes such as MRI DWI positive) the event should be considered a stroke regardless of the duration of signs and symptoms.

24.6 Heart failure

The definition of heart failure has been adapted from clinical guidelines[98,99]. Heart failure is defined as a clinical syndrome with at least one consistent sign (from tachycardia, tachypnea, pulmonary crepitation's, pleural effusion, raised jugular venous pressure, peripheral edema, or hepatomegaly) or symptom (from dyspnea, fatigue, dependent edema, symptoms of splanchnic congestion) of heart failure, plus either one positive diagnostic test (such as BNP > 400 or NT-pro BNP > ULN or chest X-ray showing pulmonary congestion, edema, pleural effusion, or cardiomegaly, or echocardiographic abnormality).

24.7 Angina

24.7.1 Unstable angina

Defined as ischemic symptoms (pain, dyspnea, pressure) at rest or with minimal exertion that the investigator determines is secondary to ischemia and requires hospitalization.

And

New ECG changes

- New ST depression horizontal or down sloping ≥ 0.05 mV; or
- New T wave inversion ≥ 0.1mV

If a participant simultaneously meets criteria for both MI and unstable angina, they will be considered to have had an MI.

24.7.2 Worsening angina

Defined as known angina increasing in frequency, duration, and/or severity, and requiring increased anti-anginal medications or dosage of these medications or coronary revascularization.
If a participant simultaneously meets criteria for both MI and worsening angina, they will be considered to have had an MI.

24.7.3 New angina

Defined as new onset of typical angina with documented ischemia by stress testing or stress imaging in a patient previously not known to have angina at baseline.
New angina also includes the following two types of cases:

- Angina occurring 6 months or more after CABG/PCI
- Angina occurring any time after CABG/PCI if the subject did not have angina before the procedure
- If a participant simultaneously meets criteria for both MI and new angina, they will be considered to have had an MI.

24.8 Venous thromboembolism

Venous thromboembolism is defined as symptomatic lower limb deep vein thrombosis or pulmonary embolism confirmed by objective testing.

Criteria for the objective confirmation of deep vein thrombosis include:

- A constant filling defect in two or more views on contrast venography in one or more proximal venous segments (iliac, common femoral, superficial femoral, popliteal)
- New or previously undocumented non-compressibility of one or more venous segments on compression ultrasound
- A clearly defined intraluminal filling defect on contrast enhanced computed tomography

Criteria for the objective diagnosis of pulmonary embolism include:

- An intraluminal filling defect on pulmonary angiography
- Sudden contrast cut-off of one or more on a pulmonary angiogram
- A high probability ventilation-perfusion scan (one or more segmental perfusion defects with corresponding normal ventilation)
- An abnormal non-high ventilation-perfusion scan plus criteria for the diagnosis of deep vein thrombosis
- An unequivocal, intra-arterial, filling defect in the central pulmonary vasculature (pulmonary trunk, main pulmonary arteries, anterior trunk, right and left interlobar and lobar arteries) on computed tomography pulmonary angiography

24.9 Castrate-Resistant PC

Castrate-resistant PC will be defined as either

a) three consecutive increases in PSA concentration at least one month apart, or

b) evidence of new clinical disease with serum testosterone concentration <1.7mmol/L (50ng/dL)[100].

24.10 Distant PC Progression

- In participants with no evidence of metastases outside the prostatic region at the baseline visit, distant PC progression will be defined as the development of metastases outside the prostatic region during follow-up. New metastases may include bone metastases, or pelvic or retroperitoneal lymphadenopathy
- In participants with metastases outside the prostatic region at the baseline visit, distant PC progression will be defined as either
  a. The development of new metastases outside the prostatic region, where new metastases may include bone metastases, or pelvic or retroperitoneal lymphadenopathy and/or
  b. Enlargement of existing metastases

24.11 PSA Progression

PSA progression will be defined by the presence of both an increase in serum PSA concentration of ≥2μg/L and ≥25% from its nadir concentration after PC diagnosis[100].

24.12 Biochemical Failure

Biochemical failure will be defined as either:

a) in men who have undergone radical prostatectomy, the presence of a detectable serum PSA concentration, or
b) in men who have undergone radiotherapy to the prostate, the occurrence of PSA progression[101]

24.13 Major bleeding

Major bleeding will be defined according to the modified International Society of Thrombosis and Hemostasis criteria as:

a. Fatal bleeding and/or
b. Symptomatic bleeding in a critical area or organ, such as intracranial, intraspinal, intraocular, retroperitoneal, intraarticular or pericardial, or intramuscular with compartment syndrome, or bleeding into the surgical site requiring re-operation and/or
c. Bleeding leading to hospitalization.

24.14 Myositis

Myositis will be defined as the presence of skeletal muscle symptoms (pain or weakness) in association with a serum creatine kinase concentration >10x the upper limit of normal.[86]

24.15 Liver injury

Liver injury will be defined as[87] elevation in alanine transaminase or aspartate transaminase, together with one of:

a. Hepatomegaly
b. Jaundice
c. Elevated direct bilirubin concentration
d. Increased prothrombin time.

24.16 Kidney injury

Kidney injury will be defined by[88] a 2-fold or greater increase in serum creatinine concentration from the most recent serum creatinine concentration, or a decrease in estimated glomeraular filtration rate of more than 50% from the most recent estimated glomerular filtration rate.

24.17 Hospitalization

Any overnight stay in a hospital ward, including the Emergency Department, will be considered a hospitalization.

References

1. Van Hemelrijck M, Folkvaljon Y, Adolfsson J, Akre O, Holmberg L, Garmo H, Stattin P. Causes of death in men with localized prostate cancer: a nationwide, population-based study. *BJU Int*. 2015. doi: 10.1111/bju.13059
2. Keating NL, O'Malley AJ, Freedland SJ, Smith MR. Diabetes and cardiovascular disease during androgen deprivation therapy: observational study of veterans with prostate cancer. *J Natl Cancer Inst*. 2010;102:39–46. doi: 10.1093/jnci/djp404
3. O'Farrell S, Garmo H, Holmberg L, Adolfsson J, Stattin P, Van Hemelrijck M. Risk and timing of cardiovascular disease after androgen-deprivation therapy in men with prostate cancer. *J Clin Oncol*. 2015;33:1243–1251. doi: 10.1200/JCO.2014.59.1792
4. Haque R, UlcickasYood M, Xu X, Cassidy-Bushrow AE, Tsai HT, Keating NL, Van Den Eeden SK, Potosky AL. Cardiovascular disease risk and androgen deprivation therapy in patients with localised prostate cancer: a prospective cohort study. *Br J Cancer*. 2017;117:1233–1240. doi: 10.1038/bjc.2017.280
5. Greenland P, Alpert JS, Beller GA, Benjamin EJ, Budoff MJ, Fayad ZA, Foster E, Hlatky MA, Hodgson JM, Kushner FG, et al. 2010 ACCF/AHA guideline for assessment of cardiovascular risk in asymptomatic adults: a report of the American College of Cardiology Foundation/American Heart Association Task Force on Practice Guidelines. *J Am Coll Cardiol*. 2010;56:e50–103. doi: 10.1016/j.jacc.2010.09.001
6. Huggins C, Stevens RE, Jr, Hodges CV. Studies on prostatic cancer: Ii. the effects of castration on advanced carcinoma of the prostate gland. *Archives of Surgery*. 1941;43:209–223. doi: 10.1001/archsurg.1941.01210140043004
7. Nguyen PL, Je Y, Schutz FA, Hoffman KE, Hu JC, Parekh A, Beckman JA, Choueiri TK. Association of androgen deprivation therapy with cardiovascular death in patients with prostate cancer: a meta-analysis of randomized trials. *Jama*. 2011;306:2359–2366. doi: 10.1001/jama.2011.1745
8. Shahinian VB, Kuo YF, Gilbert SM. Reimbursement policy and androgen-deprivation therapy for prostate cancer. *N Engl J Med*. 2010;363:1822–1832. doi: 10.1056/NEJMsa0910784
9. Zhao J, Zhu S, Sun L, Meng F, Zhao L, Zhao Y, Tian H, Li P, Niu Y. Androgen deprivation therapy for prostate cancer is associated with cardiovascular morbidity and mortality: a meta-analysis of population-based observational studies. *PLoS One*. 2014;9:e107516. doi: 10.1371/journal.pone.0107516
10. Bosco C, Bosnyak Z, Malmberg A, Adolfsson J, Keating NL, Van Hemelrijck M. Quantifying Observational Evidence for Risk of Fatal and Nonfatal Cardiovascular Disease Following Androgen Deprivation Therapy

for Prostate Cancer: A Meta-analysis. *European urology*. 2014. doi: 10.1016/j.eururo.2014.11.039

11. Kim J, Vaid M, Tyldesley S, Woods R, Pickles T. Population-based study of cardiovascular mortality among patients with prostate cancer treated with radical external beam radiation therapy with and without adjuvant androgen deprivation therapy at the British Columbia Cancer Agency. *Int J Radiat Oncol Biol Phys*. 2011;80:742–750. doi: 10.1016/j.ijrobp.2010.03.018
12. Levine GN, D'Amico AV, Berger P, Clark PE, Eckel RH, Keating NL, Milani RV, Sagalowsky AI, Smith MR, Zakai N. Androgen-deprivation therapy in prostate cancer and cardiovascular risk: a science advisory from the American Heart Association, American Cancer Society, and American Urological Association: endorsed by the American Society for Radiation Oncology. *Circulation*. 2010;121:833–840. doi: 10.1161/CIRCULATIONAHA.109.192695
13. McQueen MJ, Hawken S, Wang X, Ounpuu S, Sniderman A, Probstfield J, Steyn K, Sanderson JE, Hasani M, Volkova E, et al. Lipids, lipoproteins, and apolipoproteins as risk markers of myocardial infarction in 52 countries (the INTERHEART study): a case-control study. *Lancet*. 2008;372:224–233. doi: 10.1016/S0140-6736(08)61076-4
14. Smith JC, Bennett S, Evans LM, Kynaston HG, Parmar M, Mason MD, Cockcroft JR, Scanlon MF, Davies JS. The effects of induced hypogonadism on arterial stiffness, body composition, and metabolic parameters in males with prostate cancer. *J Clin Endocrinol Metab*. 2001;86:4261–4267. doi: 10.1210/jcem.86.9.7851
15. Smith MR, Finkelstein JS, McGovern FJ, Zietman AL, Fallon MA, Schoenfeld DA, Kantoff PW. Changes in body composition during androgen deprivation therapy for prostate cancer. *J Clin Endocrinol Metab*. 2002;87:599–603. doi: 10.1210/jcem.87.2.8299
16. Leong DP, Teo KK, Rangarajan S, Lopez-Jaramillo P, Avezum A, Jr., Orlandini A, Seron P, Ahmed SH, Rosengren A, Kelishadi R, et al. Prognostic value of grip strength: findings from the Prospective Urban Rural Epidemiology (PURE) study. *Lancet*. 2015. doi: 10.1016/S0140-6736(14)62000-6
17. Yusuf S, Hawken S, Ounpuu S, Bautista L, Franzosi MG, Commerford P, Lang CC, Rumboldt Z, Onen CL, Lisheng L, et al. Obesity and the risk of myocardial infarction in 27,000 participants from 52 countries: a case-control study. *Lancet*. 2005;366:1640–1649. doi: 10.1016/S0140-6736(05)67663-5
18. Taylor AE, Ebrahim S, Ben-Shlomo Y, Martin RM, Whincup PH, Yarnell JW, Wannamethee SG, Lawlor DA. Comparison of the associations of body mass index and measures of central adiposity and fat mass with coronary heart disease, diabetes, and all-cause mortality: a study using data from 4 UK cohorts. *Am J Clin Nutr*. 2010;91:547–556. doi: 10.3945/ajcn.2009.28757

19. Romo ML, McCrillis AM, Brite J, Reales D, Dowd JB, Schooling CM. Pharmacologic androgen deprivation and cardiovascular disease risk factors: a systematic review. *Eur J Clin Invest*. 2015;45:475–484. doi: 10.1111/eci.12431
20. Yu IC, Lin HY, Sparks JD, Yeh S, Chang C. Androgen Receptor Roles in Insulin Resistance and Obesity in Males: The Linkage of Androgen-Deprivation Therapy to Metabolic Syndrome. *Diabetes*. 2014;63:3180–3188. doi: 10.2337/db13-1505
21. Hopmans SN, Duivenvoorden WC, Werstuck GH, Klotz L, Pinthus JH. GnRH antagonist associates with less adiposity and reduced characteristics of metabolic syndrome and atherosclerosis compared with orchiectomy and GnRH agonist in a preclinical mouse model. *Urol Oncol*. 2014. doi: 10.1016/j.urolonc.2014.06.018
22. Albertsen PC, Klotz L, Tombal B, Grady J, Olesen TK, Nilsson J. Cardiovascular morbidity associated with gonadotropin releasing hormone agonists and an antagonist. *Eur Urol*. 2014;65:565–573. doi: 10.1016/j.eururo.2013.10.032
23. Prasad SM, Eggener SE, Lipsitz SR, Irwin MR, Ganz PA, Hu JC. Effect of depression on diagnosis, treatment, and mortality of men with clinically localized prostate cancer. *J Clin Oncol*. 2014;32:2471–2478. doi: 10.1200/JCO.2013.51.1048
24. Joly F, Alibhai SM, Galica J, Park A, Yi QL, Wagner L, Tannock IF. Impact of androgen deprivation therapy on physical and cognitive function, as well as quality of life of patients with nonmetastatic prostate cancer. *J Urol*. 2006;176:2443–2447. doi: 10.1016/j.juro.2006.07.151
25. Yang J, Zhong F, Qiu J, Wang K. Cognitive function in Chinese prostate cancer patients on androgen-deprivation therapy: A cross-sectional study. *Asia Pac J Clin Oncol*. 2015. doi: 10.1111/ajco.12347
26. Alibhai SM, Breunis H, Timilshina N, Marzouk S, Stewart D, Tannock I, Naglie G, Tomlinson G, Fleshner N, Krahn M, et al. Impact of androgen-deprivation therapy on cognitive function in men with nonmetastatic prostate cancer. *J Clin Oncol*. 2010;28:5030–5037. doi: 10.1200/JCO.2010.30.8742
27. Moore TH, King AJ, Evans M, Sharp D, Persad R, Huntley AL. Supportive care for men with prostate cancer: why are the trials not working? A systematic review and recommendations for future trials. *Cancer Med*. 2015. doi: 10.1002/cam4.446
28. Sergi G, Veronese N, Fontana L, De Rui M, Bolzetta F, Zambon S, Corti MC, Baggio G, Toffanello ED, Crepaldi G, et al. Pre-frailty and risk of cardiovascular disease in elderly men and women: the pro.v.a. Study. *J Am Coll Cardiol*. 2015;65:976–983. doi: 10.1016/j.jacc.2014.12.040
29. Fried LP, Tangen CM, Walston J, Newman AB, Hirsch C, Gottdiener J, Seeman T, Tracy R, Kop WJ, Burke G, et al. Frailty in older adults: evidence for a phenotype. *J Gerontol A Biol Sci Med Sci*. 2001;56:M146–156.

30. Yusuf S, Hawken S, Ounpuu S, Dans T, Avezum A, Lanas F, McQueen M, Budaj A, Pais P, Varigos J, et al. Effect of potentially modifiable risk factors associated with myocardial infarction in 52 countries (the INTERHEART study): case-control study. *Lancet*. 2004;364:937–952.
31. Ford ES, Ajani UA, Croft JB, Critchley JA, Labarthe DR, Kottke TE, Giles WH, Capewell S. Explaining the decrease in U.S. deaths from coronary disease, 1980-2000. *N Engl J Med*. 2007;356:2388–2398. doi: 10.1056/NEJMsa053935
32. Stamler J, Wentworth D, Neaton JD. Is relationship between serum cholesterol and risk of premature death from coronary heart disease continuous and graded? Findings in 356,222 primary screenees of the Multiple Risk Factor Intervention Trial (MRFIT). *JAMA*. 1986;256:2823–2828.
33. Baigent C, Keech A, Kearney PM, Blackwell L, Buck G, Pollicino C, Kirby A, Sourjina T, Peto R, Collins R, et al. Efficacy and safety of cholesterol-lowering treatment: prospective meta-analysis of data from 90,056 participants in 14 randomised trials of statins. *Lancet*. 2005;366:1267–1278. doi: 10.1016/S0140-6736(05)67394-1
34. Anderson TJ, Gregoire J, Hegele RA, Couture P, Mancini GB, McPherson R, Francis GA, Poirier P, Lau DC, Grover S, et al. 2012 update of the Canadian Cardiovascular Society guidelines for the diagnosis and treatment of dyslipidemia for the prevention of cardiovascular disease in the adult. *Can J Cardiol*. 2013;29:151–167. doi: 10.1016/j.cjca.2012.11.032
35. Desai CS, Martin SS, Blumenthal RS. Non-cardiovascular effects associated with statins. *BMJ*. 2014;349:g3743. doi: 10.1136/bmj.g3743
36. Parker BA, Capizzi JA, Grimaldi AS, Clarkson PM, Cole SM, Keadle J, Chipkin S, Pescatello LS, Simpson K, White CM, et al. Effect of statins on skeletal muscle function. *Circulation*. 2013;127:96–103. doi: 10.1161/CIRCULATIONAHA.112.136101
37. Park HS, Schoenfeld JD, Mailhot RB, Shive M, Hartman RI, Ogembo R, Mucci LA. Statins and prostate cancer recurrence following radical prostatectomy or radiotherapy: a systematic review and meta-analysis. *Ann Oncol*. 2013;24:1427–1434. doi: 10.1093/annonc/mdt077
38. Klimis H, Pinthus JH, Aghel N, Duceppe E, Fradet V, Brown I, Siemens DR, Shayegan B, Klotz L, Luke PP, et al. The Burden of Uncontrolled Cardiovascular Risk Factors in Men With Prostate Cancer: A RADICAL-PC Analysis. *JACC CardioOncol*. 2023;5:70–81. doi: 10.1016/j.jaccao.2022.09.008
39. Lewington S, Clarke R, Qizilbash N, Peto R, Collins R, Prospective Studies C. Age-specific relevance of usual blood pressure to vascular mortality: a meta-analysis of individual data for one million adults in 61 prospective studies. *Lancet*. 2002;360:1903–1913.
40. Domanski M, Mitchell G, Pfeffer M, Neaton JD, Norman J, Svendsen K, Grimm R, Cohen J, Stamler J, Group MR. Pulse pressure and

cardiovascular disease-related mortality: follow-up study of the Multiple Risk Factor Intervention Trial (MRFIT). *JAMA*. 2002;287:2677–2683.

41. Schocken DD, Benjamin EJ, Fonarow GC, Krumholz HM, Levy D, Mensah GA, Narula J, Shor ES, Young JB, Hong Y. Prevention of heart failure: A scientific statement from the American Heart Association Councils on epidemiology and prevention, clinical cardiology, cardiovascular nursing, and high blood pressure research; Quality of Care and Outcomes Research Interdisciplinary Working Group; and Functional Genomics and Translational Biology Interdisciplinary Working Group. *Circulation*. 2008;117:2544–2565. doi: http://dx.doi.org/10.1161/CIRCULATIONAHA.107.188965
42. Turnbull F, Blood Pressure Lowering Treatment Trialists C. Effects of different blood-pressure-lowering regimens on major cardiovascular events: results of prospectively-designed overviews of randomised trials. *Lancet*. 2003;362:1527–1535.
43. Farnett L, Mulrow CD, Linn WD, Lucey CR, Tuley MR. The J-curve phenomenon and the treatment of hypertension. Is there a point beyond which pressure reduction is dangerous? *JAMA*. 1991;265:489–495.
44. Group JS. Principal results of the Japanese trial to assess optimal systolic blood pressure in elderly hypertensive patients (JATOS). *Hypertens Res*. 2008;31:2115–2127. doi: 10.1291/hypres.31.2115
45. Verdecchia P, Staessen JA, Angeli F, de Simone G, Achilli A, Ganau A, Mureddu G, Pede S, Maggioni AP, Lucci D, et al. Usual versus tight control of systolic blood pressure in non-diabetic patients with hypertension (Cardio-Sis): an open-label randomised trial. *Lancet*. 2009;374:525–533. doi: 10.1016/S0140-6736(09)61340-4
46. Group SR, Wright JT, Jr., Williamson JD, Whelton PK, Snyder JK, Sink KM, Rocco MV, Reboussin DM, Rahman M, Oparil S, et al. A Randomized Trial of Intensive versus Standard Blood-Pressure Control. *N Engl J Med*. 2015;373:2103–2116. doi: 10.1056/NEJMoa1511939
47. Lonn EM, Bosch J, Lopez-Jaramillo P, Zhu J, Liu L, Pais P, Diaz R, Xavier D, Sliwa K, Dans A, et al. Blood-Pressure Lowering in Intermediate-Risk Persons without Cardiovascular Disease. *N Engl J Med*. 2016;374:2009–2020. doi: 10.1056/NEJMoa1600175
48. Leung AA, Nerenberg K, Daskalopoulou SS, McBrien K, Zarnke KB, Dasgupta K, Cloutier L, Gelfer M, Lamarre-Cliche M, Milot A, et al. Hypertension Canada's 2016 Canadian Hypertension Education Program Guidelines for Blood Pressure Measurement, Diagnosis, Assessment of Risk, Prevention, and Treatment of Hypertension. *Can J Cardiol*. 2016;32:569–588. doi: 10.1016/j.cjca.2016.02.066
49. James PA, Oparil S, Carter BL, Cushman WC, Dennison-Himmelfarb C, Handler J, Lackland DT, LeFevre ML, MacKenzie TD, Ogedegbe O, et al. 2014 evidence-based guideline for the management of high blood pressure in adults: report from the panel members appointed to the Eighth Joint National Committee (JNC 8). *JAMA*. 2014;311:507–520. doi: 10.1001/jama.2013.284427

50. Yusuf S, Sleight P, Pogue J, Bosch J, Davies R, Dagenais G. Effects of an angiotensin-converting-enzyme inhibitor, ramipril, on cardiovascular events in high-risk patients. The Heart Outcomes Prevention Evaluation Study Investigators. *N Engl J Med*. 2000;342:145–153. doi: 10.1056/NEJM200001203420301
51. Lonn E, Shaikholeslami R, Yi Q, Bosch J, Sullivan B, Tanser P, Magi A, Yusuf S. Effects of ramipril on left ventricular mass and function in cardiovascular patients with controlled blood pressure and with preserved left ventricular ejection fraction: a substudy of the Heart Outcomes Prevention Evaluation (HOPE) Trial. *J Am Coll Cardiol*. 2004;43:2200–2206. doi: 10.1016/j.jacc.2003.10.073
52. Fox KM, Investigators EUtOrocewPiscAd. Efficacy of perindopril in reduction of cardiovascular events among patients with stable coronary artery disease: randomised, double-blind, placebo-controlled, multicentre trial (the EUROPA study). *Lancet*. 2003;362:782–788.
53. Braunwald E, Domanski MJ, Fowler SE, Geller NL, Gersh BJ, Hsia J, Pfeffer MA, Rice MM, Rosenberg YD, Rouleau JL, et al. Angiotensin-converting-enzyme inhibition in stable coronary artery disease. *N Engl J Med*. 2004;351:2058–2068. doi: 10.1056/NEJMoa042739
54. Effect of enalapril on survival in patients with reduced left ventricular ejection fractions and congestive heart failure. The SOLVD Investigators. *N Engl J Med*. 1991;325:293–302.
55. Reardon LC, Macpherson DS. Hyperkalemia in outpatients using angiotensin-converting enzyme inhibitors. How much should we worry? *Arch Intern Med*. 1998;158:26–32.
56. National Kidney F. KDOQI Clinical Practice Guideline for Diabetes and CKD: 2012 Update. *Am J Kidney Dis*. 2012;60:850–886. doi: 10.1053/j.ajkd.2012.07.005
57. Teo KK, Ounpuu S, Hawken S, Pandey MR, Valentin V, Hunt D, Diaz R, Rashed W, Freeman R, Jiang L, et al. Tobacco use and risk of myocardial infarction in 52 countries in the INTERHEART study: a case-control study. *Lancet*. 2006;368:647–658. doi: 10.1016/S0140-6736(06)69249-0
58. Ockene IS, Miller NH. Cigarette smoking, cardiovascular disease, and stroke: a statement for healthcare professionals from the American Heart Association. American Heart Association Task Force on Risk Reduction. *Circulation*. 1997;96:3243–3247.
59. Mons U, Muezzinler A, Gellert C, Schottker B, Abnet CC, Bobak M, de Groot L, Freedman ND, Jansen E, Kee F, et al. Impact of smoking and smoking cessation on cardiovascular events and mortality among older adults: meta-analysis of individual participant data from prospective cohort studies of the CHANCES consortium. *BMJ*. 2015;350:h1551. doi: 10.1136/bmj.h1551
60. Stead LF, Buitrago D, Preciado N, Sanchez G, Hartmann-Boyce J, Lancaster T. Physician advice for smoking cessation. *Cochrane Database Syst Rev*. 2013;5:CD000165. doi: 10.1002/14651858.CD000165.pub4

61. Cohen SJ, Stookey GK, Katz BP, Drook CA, Smith DM. Encouraging primary care physicians to help smokers quit. A randomized, controlled trial. *Ann Intern Med*. 1989;110:648–652.
62. Emerging Risk Factors C, Wormser D, Kaptoge S, Di Angelantonio E, Wood AM, Pennells L, Thompson A, Sarwar N, Kizer JR, Lawlor DA, et al. Separate and combined associations of body-mass index and abdominal adiposity with cardiovascular disease: collaborative analysis of 58 prospective studies. *Lancet*. 2011;377:1085–1095. doi: 10.1016/S0140-6736(11)60105-0
63. Schauer PR, Bhatt DL, Kirwan JP, Wolski K, Brethauer SA, Navaneethan SD, Aminian A, Pothier CE, Kim ES, Nissen SE, et al. Bariatric surgery versus intensive medical therapy for diabetes--3-year outcomes. *N Engl J Med*. 2014;370:2002–2013. doi: 10.1056/NEJMoa1401329
64. Leong DP, Fradet V, Niazi T, Selvanayagam JB, Sabbagh R, Higano CS, Agapay S, Rangarajan S, Mian R, Nakashima CAK, et al. Adiposity and Muscle Strength in Men With Prostate Cancer and Cardiovascular Outcomes. *JACC CardioOncol*. 2024;6:761–771. doi: 10.1016/j.jaccao.2024.07.011
65. Dehghan M, Mente A, Teo KK, Gao P, Sleight P, Dagenais G, Avezum A, Probstfield JL, Dans T, Yusuf S. Relationship between healthy diet and risk of cardiovascular disease among patients on drug therapies for secondary prevention: a prospective cohort study of 31 546 high-risk individuals from 40 countries. *Circulation*. 2012;126:2705–2712. doi: 10.1161/CIRCULATIONAHA.112.103234
66. Estruch R, Ros E, Salas-Salvado J, Covas MI, Corella D, Aros F, Gomez-Gracia E, Ruiz-Gutierrez V, Fiol M, Lapetra J, et al. Primary prevention of cardiovascular disease with a Mediterranean diet. *N Engl J Med*. 2013;368:1279–1290. doi: 10.1056/NEJMoa1200303
67. de Lorgeril M, Renaud S, Mamelle N, Salen P, Martin JL, Monjaud I, Guidollet J, Touboul P, Delaye J. Mediterranean alpha-linolenic acid-rich diet in secondary prevention of coronary heart disease. *Lancet*. 1994;343:1454–1459.
68. Lim SS, Vos T, Flaxman AD, Danaei G, Shibuya K, Adair-Rohani H, Amann M, Anderson HR, Andrews KG, Aryee M, et al. A comparative risk assessment of burden of disease and injury attributable to 67 risk factors and risk factor clusters in 21 regions, 1990-2010: a systematic analysis for the Global Burden of Disease Study 2010. *Lancet*. 2012;380:2224–2260. doi: 10.1016/S0140-6736(12)61766-8
69. Wen CP, Wai JP, Tsai MK, Yang YC, Cheng TY, Lee MC, Chan HT, Tsao CK, Tsai SP, Wu X. Minimum amount of physical activity for reduced mortality and extended life expectancy: a prospective cohort study. *Lancet*. 2011;378:1244–1253. doi: 10.1016/S0140-6736(11)60749-6
70. Naci H, Ioannidis JP. Comparative effectiveness of exercise and drug interventions on mortality outcomes: metaepidemiological study. *BMJ*. 2013;347:f5577. doi: 10.1136/bmj.f5577

71. Galvao DA, Spry N, Denham J, Taaffe DR, Cormie P, Joseph D, Lamb DS, Chambers SK, Newton RU. A multicentre year-long randomised controlled trial of exercise training targeting physical functioning in men with prostate cancer previously treated with androgen suppression and radiation from TROG 03.04 RADAR. *Eur Urol*. 2014;65:856–864. doi: 10.1016/j.eururo.2013.09.041
72. O'Reilly GA, Spruijt-Metz D. Current mHealth technologies for physical activity assessment and promotion. *Am J Prev Med*. 2013;45:501–507. doi: 10.1016/j.amepre.2013.05.012
73. Leong DP, Smyth A, Teo KK, McKee M, Rangarajan S, Pais P, Liu L, Anand SS, Yusuf S, Investigators I. Patterns of alcohol consumption and myocardial infarction risk: observations from 52 countries in the INTERHEART case-control study. *Circulation*. 2014;130:390–398. doi: 10.1161/CIRCULATIONAHA.113.007627
74. Smyth A TK, Rangarajan S, O'Donnell M, Zhang M, Rana P, Leong D, Dagenais G, Seron P, Rosengren A, Schutte IE, Lopez-Jaramillo P, Oguz A, Chifamba J, Diaz R, Lear S, Avezum A, Kumar R, Mohan V, Szuba A, Wei L, Yang W, Jian B, McKee M, Yusuf S. Alcohol consumption and cardiovascular disease, cancer, injury, hospitalization and mortality. *Lancet*.Accepted for publication.
75. Thorat MA, Cuzick J. Prophylactic use of aspirin: systematic review of harms and approaches to mitigation in the general population. *Eur J Epidemiol*. 2015;30:5–18. doi: 10.1007/s10654-014-9971-7
76. Antithrombotic Trialists C, Baigent C, Blackwell L, Collins R, Emberson J, Godwin J, Peto R, Buring J, Hennekens C, Kearney P, et al. Aspirin in the primary and secondary prevention of vascular disease: collaborative meta-analysis of individual participant data from randomised trials. *Lancet*. 2009;373:1849–1860. doi: 10.1016/S0140-6736(09)60503-1
77. Halvorsen S, Andreotti F, ten Berg JM, Cattaneo M, Coccheri S, Marchioli R, Morais J, Verheugt FW, De Caterina R. Aspirin therapy in primary cardiovascular disease prevention: a position paper of the European Society of Cardiology working group on thrombosis. *J Am Coll Cardiol*. 2014;64:319–327. doi: 10.1016/j.jacc.2014.03.049
78. Perk J, De Backer G, Gohlke H, Graham I, Reiner Z, Verschuren M, Albus C, Benlian P, Boysen G, Cifkova R, et al. European Guidelines on cardiovascular disease prevention in clinical practice (version 2012). The Fifth Joint Task Force of the European Society of Cardiology and Other Societies on Cardiovascular Disease Prevention in Clinical Practice (constituted by representatives of nine societies and by invited experts). *Eur Heart J*. 2012;33:1635–1701. doi: 10.1093/eurheartj/ehs092
79. Vandvik PO, Lincoff AM, Gore JM, Gutterman DD, Sonnenberg FA, Alonso-Coello P, Akl EA, Lansberg MG, Guyatt GH, Spencer FA, et al. Primary and secondary prevention of cardiovascular disease: Antithrombotic Therapy and Prevention of Thrombosis, 9th ed: American College of Chest Physicians Evidence-Based Clinical Practice Guidelines. *Chest*. 2012;141:e637S–668S. doi: 10.1378/chest.11-2306

80. McNeil JJ, Wolfe R, Woods RL, Tonkin AM, Donnan GA, Nelson MR, Reid CM, Lockery JE, Kirpach B, Storey E, et al. Effect of Aspirin on Cardiovascular Events and Bleeding in the Healthy Elderly. *N Engl J Med*. 2018;379:1509–1518. doi: 10.1056/NEJMoa1805819
81. McNeil JJ, Nelson MR, Woods RL, Lockery JE, Wolfe R, Reid CM, Kirpach B, Shah RC, Ives DG, Storey E, et al. Effect of Aspirin on All-Cause Mortality in the Healthy Elderly. *N Engl J Med*. 2018;379:1519–1528. doi: 10.1056/NEJMoa1803955
82. Zheng SL, Roddick AJ. Association of Aspirin Use for Primary Prevention With Cardiovascular Events and Bleeding Events: A Systematic Review and Meta-analysis. *JAMA*. 2019;321:277–287. doi: 10.1001/jama.2018.20578
83. Stone NJ, Robinson JG, Lichtenstein AH, Bairey Merz CN, Blum CB, Eckel RH, Goldberg AC, Gordon D, Levy D, Lloyd-Jones DM, et al. 2013 ACC/AHA guideline on the treatment of blood cholesterol to reduce atherosclerotic cardiovascular risk in adults: a report of the American College of Cardiology/American Heart Association Task Force on Practice Guidelines. *Circulation*. 2014;129:S1–45. doi: 10.1161/01.cir.0000437738.63853.7a
84. Eckel RH, Jakicic JM, Ard JD, de Jesus JM, Houston Miller N, Hubbard VS, Lee IM, Lichtenstein AH, Loria CM, Millen BE, et al. 2013 AHA/ACC guideline on lifestyle management to reduce cardiovascular risk: a report of the American College of Cardiology/American Heart Association Task Force on Practice Guidelines. *Circulation*. 2014;129:S76–99. doi: 10.1161/01.cir.0000437740.48606.d1
85. Ford I, Norrie J. Pragmatic Trials. *N Engl J Med*. 2016;375:454–463. doi: 10.1056/NEJMra1510059
86. Stroes ES, Thompson PD, Corsini A, Vladutiu GD, Raal FJ, Ray KK, Roden M, Stein E, Tokgozoglu L, Nordestgaard BG, et al. Statin-associated muscle symptoms: impact on statin therapy-European Atherosclerosis Society Consensus Panel Statement on Assessment, Aetiology and Management. *Eur Heart J*. 2015;36:1012–1022. doi: 10.1093/eurheartj/ehv043
87. Cohen DE, Anania FA, Chalasani N, National Lipid Association Statin Safety Task Force Liver Expert P. An assessment of statin safety by hepatologists. *Am J Cardiol*. 2006;97:77C–81C. doi: 10.1016/j.amjcard.2005.12.014
88. Bellomo R, Ronco C, Kellum JA, Mehta RL, Palevsky P, Acute Dialysis Quality Initiative w. Acute renal failure - definition, outcome measures, animal models, fluid therapy and information technology needs: the Second International Consensus Conference of the Acute Dialysis Quality Initiative (ADQI) Group. *Crit Care*. 2004;8:R204–212. doi: 10.1186/cc2872
89. Devereaux PJ, Mrkobrada M, Sessler DI, Leslie K, Alonso-Coello P, Kurz A, Villar JC, Sigamani A, Biccard BM, Meyhoff CS, et al. Aspirin in patients undergoing noncardiac surgery. *N Engl J Med*. 2014;370:1494–1503. doi: 10.1056/NEJMoa1401105

90. Zhang H, Plutzky J, Skentzos S, Morrison F, Mar P, Shubina M, Turchin A. Discontinuation of statins in routine care settings: a cohort study. *Ann Intern Med*. 2013;158:526–534. doi: 10.7326/0003-4819-158-7-201304020-00004
91. Mampuya WM, Frid D, Rocco M, Huang J, Brennan DM, Hazen SL, Cho L. Treatment strategies in patients with statin intolerance: the Cleveland Clinic experience. *Am Heart J*. 2013;166:597–603. doi: 10.1016/j.ahj.2013.06.004
92. Denham JW, Steigler A, Lamb DS, Joseph D, Turner S, Matthews J, Atkinson C, North J, Christie D, Spry NA, et al. Short-term neoadjuvant androgen deprivation and radiotherapy for locally advanced prostate cancer: 10-year data from the TROG 96.01 randomised trial. *Lancet Oncol*. 2011;12:451–459. doi: 10.1016/S1470-2045(11)70063-8
93. Fine JP, Gray RJ. A proportional hazards model for the subdistribution of a competing risk. *Journal of the American Statistical Association*. 1999;94:496–509.
94. D'Amico AV, Chen MH, Renshaw AA, Loffredo M, Kantoff PW. Androgen suppression and radiation vs radiation alone for prostate cancer: a randomized trial. *Jama*. 2008;299:289–295. doi: 10.1001/jama.299.3.289
95. Leong DP, Teo KK, Rangarajan S, Lopez-Jaramillo P, Avezum A, Jr., Orlandini A, Seron P, Ahmed SH, Rosengren A, Kelishadi R, et al. Prognostic value of grip strength: findings from the Prospective Urban Rural Epidemiology (PURE) study. *Lancet*. 2015;386:266–273. doi: 10.1016/S0140-6736(14)62000-6
96. Cirne F, Aghel N, Petropoulos JA, Klotz L, Lenihan DJ, Saad F, Pinthus J, Leong DP. The cardiovascular effects of gonadotropin-releasing hormone antagonists in men with prostate cancer. *Eur Heart J Cardiovasc Pharmacother*. 2022;8:253–262. doi: 10.1093/ehjcvp/pvab005
97. Thygesen K, Alpert JS, Jaffe AS, Simoons ML, Chaitman BR, White HD, Joint ESCAAHAWHFTFftUDoMI, Katus HA, Lindahl B, Morrow DA, et al. Third universal definition of myocardial infarction. *Circulation*. 2012;126:2020–2035. doi: 10.1161/CIR.0b013e31826e1058
98. Dickstein K, Cohen-Solal A, Filippatos G, McMurray JJ, Ponikowski P, Poole-Wilson PA, Stromberg A, van Veldhuisen DJ, Atar D, Hoes AW, et al. ESC Guidelines for the diagnosis and treatment of acute and chronic heart failure 2008: the Task Force for the Diagnosis and Treatment of Acute and Chronic Heart Failure 2008 of the European Society of Cardiology. Developed in collaboration with the Heart Failure Association of the ESC (HFA) and endorsed by the European Society of Intensive Care Medicine (ESICM). *Eur Heart J*. 2008;29:2388–2442. doi: ehn309 [pii] 10.1093/eurheartj/ehn309
99. Writing Committee M, Yancy CW, Jessup M, Bozkurt B, Butler J, Casey DE, Jr., Drazner MH, Fonarow GC, Geraci SA, Horwich T, et al. 2013 ACCF/AHA guideline for the management of heart failure: a report of the American College of Cardiology Foundation/American Heart Association

Task Force on practice guidelines. *Circulation*. 2013;128:e240–327. doi: 10.1161/CIR.0b013e31829e8776

100. Scher HI, Halabi S, Tannock I, Morris M, Sternberg CN, Carducci MA, Eisenberger MA, Higano C, Bubley GJ, Dreicer R, et al. Design and end points of clinical trials for patients with progressive prostate cancer and castrate levels of testosterone: recommendations of the Prostate Cancer Clinical Trials Working Group. *J Clin Oncol*. 2008;26:1148–1159. doi: 10.1200/JCO.2007.12.4487
101. Roach M, 3rd, Hanks G, Thames H, Jr., Schellhammer P, Shipley WU, Sokol GH, Sandler H. Defining biochemical failure following radiotherapy with or without hormonal therapy in men with clinically localized prostate cancer: recommendations of the RTOG-ASTRO Phoenix Consensus Conference. *Int J Radiat Oncol Biol Phys*. 2006;65:965–974. doi: 10.1016/j.ijrobp.2006.04.029
