# Supplementary material for: Specialist Referral for Cardiovascular Risk in Patients With Prostate Cancer: A Randomized Clinical Trial
Source: JAMA Intern Med. 2026 Aug 30:e264773. Online ahead of print. doi: 10.1001/jamainternmed.2026.4773 (PMC13527639; doi:10.1001/jamainternmed.2026.4773)
Supplement: Supplementary file 2 [file jamainternmed-e264773-s002.pdf]

RAndomizeD Intervention for CArdiovascular and Lifestyle Risk Factors in Prostate Cancer Patients
RADICAL-PC 2 (RCT)

STATISTICAL ANALYSIS PLAN

Version 1.0
20APR2026

Author(s):
Darryl Leong, RADICAL PC, Principal Investigator
Rajibul Mian, RADICAL PC, Study Statistician

DOCUMENT HISTORY

Version Number	Date	Description of Revision
1.0	20APRIL2026	Final Version 1.0

APPROVAL

Version #	**1.0**
Version Date	20APRIL2026

By signing the below, I designate my approval of the above-named version of the Radical-PC 2 Statistical Analysis Plan on behalf of all named authors.

**Name**	Dr. Darryl Leong
**Role**	**Principal Investigator**
**Signature**	Darryl Leong<br>Electronically signed by: Darryl Leong<br>Reason: I approve this document<br>Date: Apr 21, 2026 09:32:08 EDT
**Date** (yyyy/mm/dd)	20APRIL2026

**Name**	Dr. Jehonathan H Pinthus
**Role**	**Co- Principal Investigator**
**Signature**	Jehonathan Pinthus<br>Electronically signed by: Jehonathan Pinthus<br>Reason: I approve this document<br>Date: Apr 21, 2026 09:52:18 EDT
**Date** (yyyy/mm/dd)	20APRIL2026

.

**Name**	Dr. Shrikant I. Bangdiwala
**Role**	**Statistics Director**
**Signature**	Electronically signed by: Shrikant Bangdiwala<br>Reason: I approve this document<br>Date: Apr 20, 2026 17:08:37 EDT
**Date** (yyyy/mm/dd)	20APRIL2026

Table of Contents

APPROVAL 1
1. Introduction 5
2. Study description 5
   Background 5
   Study design 5
   Study intervention (treatment allocation) 6
   Sample size calculation 6
3. Study objectives 6
   Efficacy 6
   Primary Objective 6
   Secondary Objectives 6
4. Specific hypothesis 7
   Primary Hypothesis 7
   Secondary Hypotheses 7
   Exploratory Hypotheses 7
5. Outcomes 8
   First Primary Efficacy Outcome 8
   Second Primary Efficacy Outcome 8
   Exploratory Outcomes 8
   Safety Outcomes 8
6. Study periods 9
7. Analysis populations 9
   Intention-to-treat population 9
   On-treatment population 9
8. General Statistical Considerations 9
   General Principles 9
   Handling of Non-Compliance to Study Treatment or Follow-up 9
   Handling of Missing Data 10
   Imputation of missing date 10
   Interim Analysis and Data Monitoring 10
   Important Dates for analysis 11
   ITT data scope 11
   On-treatment (OT) data scope 11
   Censoring scheme 11

Determination of baseline values 12
Analysis of Demographics, study progress and patient characteristics 12
9. Statistical methods for analyzing study outcomes 12
Analysis of Primary Efficacy outcome 12
Analysis of the (powered) first primary effectiveness outcome 12
Analysis of the (non-powered) second primary effectiveness outcome 13
Analysis of the exploratory outcomes 13
On-treatment analyses 13
10. Subgroup Analysis 15
11. Sensitivity analysis 15
12. Adherence to the protocol 16
13. References 16
14. Tables 17

ABBREVIATIONS

ACE-I = angiotensin-converting enzyme inhibitor

ADT = androgen deprivation therapy

CRF = case report form

CV = cardiovascular

CVD = cardiovascular disease (coronary artery disease, cerebrovascular disease, heart failure, or cardiovascular death)

DSMB = Data Safety Monitoring Board

DSS = digital symbol substitution

ECOG = Eastern Cooperative Oncology Group performance status

FFQ = food frequency questionnaire

FSH= follicle stimulating hormone

GnRH = gonadotropin-releasing hormone

LDL = low-density lipoprotein cholesterol

MACE = major adverse cardiovascular events (any of myocardial infarction, stroke, heart failure, cardiovascular death, need for arterial revascularization, or hospitalization for unstable angina)

MI = myocardial infarction

PC = prostate cancer

PHQ-9 = Patient Health Questionnaire-9

PSA = prostate-specific antigen

SAGE = Standard Assessment of Global Activities in the Elderly

1. Introduction

The statistical analysis plan (SAP) specifies the details of the statistical analysis of RAndomizeD Intervention for CArdiovascular and Lifestyle Risk Factors in Prostate Cancer Patients (RADICAL PC2) described in the Clinical Study Protocol (version 6.0, dated 23DEC2025). The SAP is a working document that will be amended as new information becomes available. Approval is provided for the contents of the appendices at the time of approval. Appendices may be updated as required during the course of the study without obtaining approval for the changes; however, the author will inform those approving this document of updates to the appendices. The final version of the SAP will be signed off prior to database lock.

2. Study description

Background

Data from cancer registries in North America and Europe indicate that men with prostate cancer (PC) are at high risk for developing cardiovascular (CV) disease. Among the general population (the large majority of whom do not have prostate cancer), there is evidence to support the prevention of cardiovascular disease using both non-pharmacologic and pharmacologic approaches. The effective non-pharmacologic approaches include healthy diet and exercise, and the effective pharmacologic approaches in high-risk individuals include cholesterol-lowering using statins, blood pressure control, and in select individuals, low-dose aspirin. There is uncertainty as to whether these approaches will be beneficial in patients with prostate cancer because any cardiovascular benefit conferred by preventive strategies may be attenuated by: 1) premature mortality from the cancer, and 2) cancer therapies, such as androgen deprivation therapy (ADT, which is associated with an increased risk of cardiovascular events). In addition, management of the PC may be patients' overwhelming priority, and their motivation to address their cardiovascular risk or adhere to preventive strategies, may be low.

Study design

This pragmatic, prospective, open-label, randomized, controlled trial will include patients fulfilling any of the following criteria: a) a new diagnosis of PC (within the previous year), or b) men with PC who have commenced ADT for the first time within six months of their enrolment visit, or c) men with PC who will commence ADT for the first time within the month following their enrolment visit.

Patients who meet the eligibility criteria and who provide informed consent will be randomized to intervention or control arms via an Interactive Web Randomization System (IWRS) maintained by the coordinating centre at the Population Health Research Institute (PHRI). The randomization process uses block randomization ***stratified by centre and plan to prescribe ADT***. Study centre personnel will not know the block size.

A total of 2500 patients will be enrolled from several clinical centres globally, with an anticipated median follow-up of at least 36 months. Following randomization,

scheduled study visits will occur at 3 months after enrollment and then at 6 months after enrollment and every 6 months thereafter till 24 months and then yearly visits until study completion. All participants, irrespective of allocation group or adherence to the intervention will be followed for outcome events until the study end date. In all cases, every effort must be made to continue to follow the participant at regular study visits. Survival status and outcome information must be determined for all subjects.

Study intervention (treatment allocation)

Each subject who meets the inclusion criteria and does not meet any of the exclusion criteria will be randomly assigned to one of two intervention groups:

Intervention/Treatment arm: consultation to a cardiologist or internist,

The intervention will consist of referral of the participant to a cardiologist or internist who will administer a systematic CV and lifestyle risk factor modification strategy, including dietary and exercise advice, advice to quit smoking, and the prescription of statins, ACE-I, and/or other antihypertensive medications where appropriate, as indicated in the Study Procedures. Participants in the intervention arm will be encouraged to see the cardiologist/internist at least annually.

Control Arm: usual clinical care

The control arm will receive usual clinical care. Usual care may include referral to a cardiologist or internist, or the use of treatments included in the intervention as clinically indicated if part of the treating physician's standard practice. The management of cardiovascular risk factors and disease in control arm participants is otherwise guided by their usual healthcare providers according to local standards and guidelines.

Sample size calculation

On November 2024, using available blinded data from RADICAL PC2 and based on 1000 simulations, a sample size of 2500 randomized participants in RADICAL PC2 was estimated to have 86% power to identify a win ratio of 1.22 in the intervention arm with 34% of control-intervention pairs tied.

3. Study objectives

Efficacy

Primary Objective

To determine whether the routine referral of men within one year of their first diagnosis of PC, or who are commencing ADT for the first time, to a cardiologist or internist, who will implement a systematic CV and lifestyle risk factor modification strategy reduces adverse cardiovascular outcomes and improves cardiovascular risk factor control.

Secondary Objectives

1. To determine whether the routine referral of men within one year of their first diagnosis of PC, or who are commencing ADT for the first time, to a cardiologist

or internist, who will implement a systematic CV and lifestyle risk factor modification strategy, as compared with usual care, improves
   a. cognitive function
   b. physical function
2. To estimate the incremental cost-effectiveness ratio of the routine referral of men within one year of their first diagnosis of PC, or who are commencing ADT for the first time, to a cardiologist or internist, who will implement a systematic CV and lifestyle risk factor modification strategy.

4. Specific hypothesis

Primary Hypothesis

That the routine referral of men within one year of their first diagnosis of PC, or who are commencing ADT for the first time, to a cardiologist or internist, who will implement a systematic CV and lifestyle risk factor modification strategy reduces the risk of MACE and improves cardiovascular risk factor control.

Secondary Hypotheses

That the routine referral of men within one year of their first diagnosis of PC, or who are commencing ADT for the first time, to a cardiologist or internist, who will implement a systematic CV and lifestyle risk factor modification strategy reduces:
- the risk of MACE
- systolic blood pressure
- serum cholesterol concentration

Exploratory Hypotheses

In men with a new diagnosis of PC or who are commencing ADT for the first time:

1. That a systematic CV and lifestyle risk factor modification strategy:
   a) reduces hemoglobin A1c concentration
   b) increases handgrip strength
   c) reduces get-up-and-go time
   d) reduces waist circumference
   e) increases six-minute walk distance
   f) slows cognitive decline as measured by the DSS test

2. That a systematic CV risk factor modification (including aspirin and statin) is associated with a reduction in PC progression as measured by:
   a. prostate specific antigen concentration;
   b. in those with no evidence of metastatic disease at baseline, the development of any of: biochemical recurrence; local PC recurrence; distant metastases; or in those managed by active surveillance, the administration of curative treatment (prostatectomy or radiotherapy).

c. in those with evidence of metastatic PC at baseline, the development of castration-resistant disease; the development of biochemical, radiographic or clinical progression.

5. Outcomes

First Primary Efficacy Outcome

The powered first primary efficacy outcome will be the hierarchical composite of cardiovascular death, myocardial infarction, stroke, heart failure, cholesterol control (defined as total cholesterol ≤4mmol/L) and systolic blood pressure control (defined as systolic blood pressure ≤130mmHg).

Second Primary Efficacy Outcome

The non-powered second primary effectiveness outcome is the time to the first occurrence of the composite of cardiovascular death, myocardial infarction, stroke, heart failure.

Exploratory Outcomes

1. Total Cholesterol
2. SBP
3. Hemoglobin A1c concentration
4. Handgrip strength
5. Get-up-and-go time
6. Waist circumference
7. Six-minute walk distance
8. The DSS test
9. Prostate specific antigen concentration
10. Biochemical recurrence, defined in patients who have undergone prostatectomy as two consecutive prostate specific antigen values ≥0.2ng/mL. Biochemical recurrence is defined in patients who have undergone radiotherapy as prostate specific antigen ≥2ng/mL over the nadir concentration
11. The development of new distant metastases
12. In those managed by active surveillance, the administration of curative treatment (prostatectomy or radiotherapy)

Safety Outcomes

1. Major Bleeding
2. Fatal bleeding
3. Symptomatic bleeding
4. Bleeding leading to hospitalization.
5. Myositis
6. Liver Injury
7. Kidney Injury

6. Study periods

The study is driven by the sample size and was expected to continue until 2500 participants would be recruited and last participant would be followed for 1 year – which will ensure approximate average participant follow-up is approximately 36 months.

7. Analysis populations

<u>Intention-to-treat population</u>

The primary assessment of efficacy and other outcomes will be analyzed according to the Intention-to-treat (ITT) principle i.e., subjects (regardless of whether or not they receive intervention) are considered in the group to which they are assigned by the IWRS/randomization system.

<u>On-treatment population</u>

Intervention participants are considered "on treatment" for a period of 2 years following their most recent visit to a cardiologist or internist. Non-compliance for intervention participants would be considered the absence of a cardiologist/internist visit within 2 years of the close-out visit. If non-compliant, the date of permanent discontinuation will be defined as 2 years after the LAST visit to a cardiologist or internist.

Patients in the control group do not have a routinely scheduled visit to a cardiologist or internist. So, for patients in the control group, a visit to a cardiologist or internist would consider as switching over to intervention group – so, time to FIRST visit would be considered as permanent discontinuation from control group.

The on-treatment population will be used for the primary assessment of safety outcomes, and for sensitivity analyses of efficacy outcomes.

8. General Statistical Considerations

<u>General Principles</u>

All analyses will be performed in SAS® using version 9.4 or higher. Unless otherwise stated, all hypothesis tests will be performed using two-sided tests at the 5% significance level. Continuous variables will be summarized using descriptive statistics including means, standard deviations, medians and other necessary quartiles. Qualitative or discrete variables will be summarized using counts, percentages and necessary absolute, relative frequencies.

<u>Handling of Non-Compliance to Study Treatment or Follow-up</u>

A subject who signed an informed consent form, and, for any reason (e.g., failure to satisfy the inclusion and exclusion criteria) terminates the study without being randomized is regarded as a "screening failure". Since not randomized, they are not part of the ITT population.

In this study, outcome events and clinical data will be collected until the end of the study (EOS), even if patients are not adherent to the study intervention. Every effort will be made to continue to follow the subject and survival status information must be determined for all subjects at the end of the study.

A subject will be declared to have incomplete follow-up or to be lost to follow-up (i.e., to be completely non-compliant to follow-up) if, despite of all possible efforts, all investigators, dedicated site staff, and/or PHRI Project Office (as applicable and as local regulations allow) are not able to contact the subject or a third party (e.g., family member, doctor). Every possible effort will be made to contact the subject or a third party and to determine the outcome and survival status and reason for discontinuation as local law permits. In case of no contact, the participant will be censored on their last day of available contact during the study.

If it is documented in the database that the subject is alive at the end of the study, the subject will not be classified as lost to follow-up, but as alive.

Handling of Missing Data

Unless otherwise indicated in this section, missing values within follow-up will be treated as 'missing'. No attempt will be made to use a computerized algorithm to impute missing post-randomization values and only observed values will be used for analysis.

Imputation of missing date

When an event date is not known, the site investigator will be asked to provide a best estimate as to when the event occurred. Even though the exact date of an event is unknown, the investigator often does know some information that would indicate the approximate date, such as the first week of a month, in the fall of a year, the middle of a particular year or at least the date when the patient was last seen or contacted. This information can be meaningfully incorporated into the estimated date recorded, as this is likely to be closer to the true date than any produced by an uninformed computer program. This estimated date should be the middle date within the period that the event is known to have occurred. If the event is known to have occurred in the first week of a month, then the date in the middle of that week should be recorded as the estimate. If it occurred in the fall of a year, then the middle date in the fall is the appropriate estimate. If no information is known, then the date in the middle of the plausible time period should be given, based on the last contact with the patient prior to the event and the date of contact when information about the event was known. This method for date estimation has been used in many studies and is recommended by Dubois and Hebert.[(4)]

Interim Analysis and Data Monitoring

An independent Data Monitoring Committee (DMC) will review compliance, all Serious Adverse Event (SAE), Adverse Drug Effect (ADE), primary outcome data on a periodic basis.

No formal interim analyses of efficacy will be performed in RADICAL PC2, although DMC members will be provided data stratified by treatment allocation.

Recommendations to terminate RADICAL PC2 early will be based on the totality of effects that include efficacy and safety.

Important Dates for analysis

For each subject, the following individual data are of relevance for analysis:

- Randomization date: The date of randomization to intervention
- End of Study (EOS) Visit date: This is the date of the EOS Visit for the individual subject. At the end of their study period (site closure or study closure), a final visit is completed for all living subjects.
- Date of the last trial contact: The date of the last documented contact with the subject or a third party (including data on subject survival status). For subjects who die after randomization, the date of the last contact is set to the death date.

ITT data scope

The ITT data scope includes all outcome events observed from randomization until the EOS (or last trial contact dates). This will be the primary data scope for the analysis and will include the censoring scheme detailed in later sections.

On-treatment (OT) data scope

For the treatment emergent or on treatment (OT) data scope, all events from first intake of study intervention - until acknowledgement of Final visit to a cardiologist or internist - will be counted. This on-treatment data scope will be applied to sensitivity analysis of the primary and secondary efficacy outcomes and the main analysis of safety outcomes as specified in later Sections.

Censoring scheme

For the analysis of time to event outcomes, the censoring scheme under ITT population will be applied as follows.

- Patients will be censored at the earliest of the death date, withdrawal-of-consent, last contact date, the efficacy cut-off date, and the date that that study intervention is permanently discontinued due to a clinically meaningful reason. The date of permanent discontinuation will be defined as 2 years after the LAST visit to a cardiologist or internist in intervention patients considered non-compliant with the intervention.

Censoring scheme for the On-Treatment population would be defined based on following texts.

- Intervention participants are considered "on treatment" for a period of 2 years following their most recent visit to a cardiologist or internist. Non-compliance for

intervention participants would be considered in the absence of a cardiologist/internist visit within 2 years of the close-out visit. If non-compliant, the date of permanent discontinuation will be defined as 2 years after the LAST visit to a cardiologist or internist.

- Patients in the control group do not have a visit to a cardiologist or internist. So for patients in the control group, a visit to a cardiologist or internist would be considered as switching over to the intervention group – so, time to FIRST visit would be considered as permanent discontinuation from the control group.

Determination of baseline values

Baseline is defined as the last non-missing value until and including the day of randomization visit.

Analysis of Demographics, study progress and patient characteristics

Patient characteristics based on following will be summarized in tables.

- Demographics and Other Baseline Characteristics
- Medical history
- Prior and concomitant medications
- Compliance
- Visit adherence
- Adherence to Study Intervention

9. Statistical methods for analyzing study outcomes

Analysis of Primary Efficacy outcome

Analysis will be performed on the intention-to-treat population in the ITT data scope, using the previously described censoring scheme for ITT population.

Analysis of the (powered) first primary effectiveness outcome

Analysis of the primary effectiveness outcome will be based on the intention-to-treat principle and will be conducted according to the win ratio approach. The win ratio is calculated as the number of intervention-control pairs where the intervention is the "winner" as a ratio of the number of intervention-control pairs where the intervention is the "loser".

The individual components or outcomes for the Win Ratio approach are, in hierarchical order: (a) time to CV death, (b) time to first MI, (c) time to first Stroke (d) time to first Heart failure (HF) (e) Total Cholesterol and (f) SBP. Total cholesterol and SBP are binary and CV Death, MI, stroke, HF are time-to-event. For Total Cholesterol the cutoff value will be 4mmol/L (>4mmol/L vs <=4mmol/L). For SBP, the cutoff value will be 130mmHg (>130mmHg vs <=130mmHg). Total cholesterol and SBP measurements will be taken from the last available follow-up visit.

The contribution of individual components of the hierarchical composite outcome will be evaluated.

<u>Analysis of the (non-powered) second primary effectiveness outcome</u>
Analysis of the second primary effectiveness outcome will be based on the intention-to-treat principle. Estimates of cumulative incidence and the cumulative hazard functions will be provided to evaluate the timing of event occurrence and the consistency of any treatment effects at all-time points (i.e. that the two survival curves do not cross). The sub-distribution hazard ratio for intervention, along with 95% confidence intervals, will be estimated based on competing risks regression. The occurrence of non-cardiovascular death in the absence of the primary effectiveness outcome will be considered a competing risk.

The first primary effectiveness outcome will be tested with a type 1 error rate of 5%. For the second primary effectiveness outcome, to ensure that the overall type-I error rate at the final analysis is maintained, a gatekeeping hierarchical hypothesis testing strategy will be used. The second primary effectiveness outcome will be tested (at 5% error rate) only if the test of the first primary effectiveness outcome meets the 5% threshold (p-value<0.05).

<u>Analysis of the exploratory outcomes</u>

For analyses of continuous outcomes, suitable statistical model will be used. For analysis of repeated measurements over time, an interaction term between allocation groups (intervention vs control) and visit (baseline, visit1, visit2 ,...) will be added to the model. A significant interaction term will indicate a difference in the trajectory of these variables over time.

For binary and categorical outcomes – suitable statistical models (not limited to - Logistic(binary/multinomial/ordinal) regression, modified Poisson) will be used. For survival outcomes, competing risk analysis will be performed considering non-cardiovascular death as the competing event.

<u>On-treatment analyses</u>
For all outcomes (efficacy, safety, exploratory), exploratory analyses will be undertaken on an on-treatment basis. For these analyses – population will be defined as On-treatment population and censoring scheme used will be as defined in the previous section for on-treatment population. Otherwise, the statistical methods used in the on-treatment analyses will be the same as the ITT analyses.

Following Table gives the name of analysis technique for each outcome.

Table: Stats Methods in Summary for analysis

Outcomes	Type	Statistical methods
**Powered 1st Primary Outcomes**		
The individual hierarchy components or outcomes for the Win Ratio approach are (a) time to CV death, (b) time to first MI, (c) time to first Stroke (d) time to first Heart failure (HF) (e) Total Cholesterol and (f) SBP.	Hierarchical combination of time to event and binary outcomes	Win Ratio
**Non-powered 2nd Primary outcomes**		
Time to the first occurrence of the composite of cardiovascular death, myocardial infarction, stroke, heart failure.	Time to event	Sub distribution hazard model
**Exploratory Outcomes**		
Total cholesterol	Continuous	Mean difference - LMM
SBP	Continuous	Mean difference - LMM
Hemoglobin A1c	Continuous	Mean difference - LMM
Handgrip strength	Continuous	Mean difference - LMM
Get-up-and-go time	Continuous	Mean difference - LMM
Waist circumference	Continuous	Mean difference - LMM
Six-minute walk distance	Continuous	Mean difference - LMM
DSS test	Score	Mean difference - LMM
Prostate specific antigen concentration	Continuous	Mean difference - LMM
Biochemical recurrence, defined in patients who have undergone prostatectomy as two consecutive prostate specific antigen rises of ≥0.2ng/mL.	Binary	Logistic regression, or modified Poisson
Biochemical recurrence is defined in patients who have undergone radiotherapy as prostate specific antigen ≥2ng/mL over the nadir concentration	Binary	Logistic regression, or modified Poisson
The development of new distant metastases<br>In those managed by active surveillance, the administration of curative treatment (prostatectomy or radiotherapy)	Binary	Logistic regression, or modified Poisson
**Safety Outcomes**		
Major Bleeding	Survival	Sub distribution hazard model
Fatal bleeding	Survival	Sub distribution hazard model
Symptomatic bleeding	Survival	Sub distribution hazard model
Bleeding leading to hospitalization	Survival	Sub distribution hazard model
Myositis	Survival	Sub distribution hazard model
Liver Injury	Binary/Survival	Sub distribution hazard model
Kidney Injury	Binary/Survival	Sub distribution hazard model

10. Subgroup Analysis

The homogeneity of intervention effect will be examined for the following subgroups. Effect modification will be inferred using an intervention-subgroup interaction term p-value.

All subgroup analysis will be performed based on the ITT population in the ITT data scope, with regular censoring scheme described in the previous section.

Homogeneity of intervention effect in subgroups, both in magnitude and direction will be assessed by adding a covariate for the subgroup variable and the corresponding intervention-subgroup interaction term (intervention*subgroup) to the respective Win Ratio or competing risk analysis. Significant interactions in the analysis of the primary outcome will be interpreted as "flags" to prompt further investigation for future hypothesis generation.

Following the test of interaction, intervention effect will be estimated separately within each level of a subgroup variable using the similar Win Ratio or competing risk analysis as the one used in the primary analysis of the study outcome.

Subgroups based on the following variables will be considered for the subgroup analysis.

- ADT (yes, no)
- ADT type
- Age (<65, 65-75, >75 years)
- PC stage (metastatic, non-metastatic)
- PC treatment strategy (active surveillance, prostatectomy, radiotherapy, ADT only)
- Past history of CVD (yes, no)
- Past history of diabetes (yes, no)
- Self-reported hypertension (yes, no)
- Baseline systolic blood pressure ≥140mmHg or diastolic blood pressure ≥90mmHg
- Baseline systolic blood pressure ≥130mmHg or diastolic blood pressure ≥80mmHg
- Baseline use of lipid-lowering medication (yes, no)
- Baseline cholesterol ≤4mmol/L (yes, no)
- Baseline use of Aspirin and Statin (yes, no)

11. Sensitivity analysis

To support the primary study results and to assess the robustness of the primary analysis, on-treatment analysis will be considered as the sensitivity analysis.

12. Adherence to the protocol

Summary statistics (counts and % of patients) for major protocol deviations by allocation category will be presented.

13. References

- Pocock, S. J., Ariti, C. A., Collier, T. J., & Wang, D. (2012). The win ratio: a new approach to the analysis of composite endpoints in clinical trials based on clinical priorities. *European heart journal*, *33*(2), 176–182. https://doi.org/10.1093/eurheartj/ehr352

- Pepe, M. S., & Mori, M. (1993). Kaplan-Meier, marginal or conditional probability curves in summarizing competing risks failure time data?. *Statistics in medicine*, *12*(8), 737–751. https://doi.org/10.1002/sim.4780120803

14. Tables

Table format for the analysis will be aligned based on target journal and will be updated as necessary. Some sample tables are as follows.

Table 1. Demographics at baseline

	**RADICAL PC2 (N=2472)**		
	**Total**	**N**	**%**
Baseline Visit CRFs Received			
Age (yr)			
Ethnicity			
White / Caucasian			
Black / African descent			
Hispanic / Latino			
South Asian / Indian			
Chinese			
Southeast Asian / Malaysian			
Persian			
Arab			
Aboriginal / Native Persons / North or South American Indian			
Other			
Education			
Less than high school			
High school			
College diploma			
University degree			
Trade school			
Employment			
Employed with income			
Employed without income			
Disabled			
Retired			
Unemployed			
Homemaker			
Student			
Tobacco use			
Never			
Current			
Former			
Alcohol use			
Never			
Current			
Former			

Table 2. Medical history at baseline

	RADICAL PC2 (N=2472)		
	**N available**	**N**	**%**
Baseline Visit CRFs Received			
Myocardial infarction			
Angina			
Coronary stent / PTCA			
CABG			
Stroke			
Diabetes mellitus			
Heart failure			
Hypertension			
Peripheral arterial disease			
Atrial fibrillation			
Chronic obstructive pulmonary disease			
Liver disease			
Cancer (non-prostate)			
Venous thromboembolism			
Pneumonia			
Arthritis			
Asthma			
Dementia			
Diagnosis of depression / anxiety			
Cerebrovascular disease			
Peptic ulcer disease			
Hemiplegia or paraplegia			
Renal disease			
Dialysis			
HIV / AIDS			
COVID-19 Infection			

Table 3. Prostate cancer characteristics at baseline

	RADICAL PC2 (N=2472)		
	**N available**	**N**	**%**
Baseline Visit CRFs Received			
Radical prostatectomy			
Prostate irradiation			
Other therapeutic prostate cancer procedures			
Observation (active surveillance or watchful waiting)			
Orchiectomy			
ADT use			
Chemotherapy			
Prostate metastasis			
