# Supplementary material for: Specialist Referral for Cardiovascular Risk in Patients With Prostate Cancer: A Randomized Clinical Trial
Source: JAMA Intern Med. 2026 Aug 30:e264773. Online ahead of print. doi: 10.1001/jamainternmed.2026.4773 (PMC13527639; doi:10.1001/jamainternmed.2026.4773)
Supplement: Supplementary file 3 [file jamainternmed-e264773-s003.pdf]

Supplemental Online Content

Leong DP, Higano C, Garcia CC, et al; for the RADICAL PC Investigators. Specialist referral for cardiovascular risk in patients with prostate cancer: a randomized clinical trial. *JAMA Intern Med.* Published online August 30, 2026.
doi:10.1001/jamainternmed.2026.4773

eTable 1. Lipid and Blood Pressure Values

eFigure 1. Clinical Event Cumulative Incidence Curves for Cardiovascular Death (CVD)

eFigure 2. Clinical Event Cumulative Incidence Curves for Myocardial Infarction (MI)

eFigure 3. Clinical Event Cumulative Incidence Curves for Stroke

eFigure 4. Clinical Event Cumulative Incidence Curves for Heart Failure (HF)

eTable 2. Safety Outcomes

eAppendix

This supplemental material has been provided by the authors to give readers additional information about their work.

© 2026 Leong DP et al. *JAMA Internal Medicine*.

eTable 1. Lipid and Blood Pressure Values

P-values are derived from linear mixed effects models with an unstructured covariance matrix.

Measurement	Visit	Intervention	Control	p-value for the between-group difference by visit	Group-visit interaction p-value
**Total cholesterol, mg/dL**	Baseline	185.6±42.5	189.5±42.5		<0.0001
	12-months	170.5±43.7	186.4±44.1	<0.0001	
	24-months	165.1±41.8	182.5±42.2	<0.0001	
	48-months	158.9±36.7	175.2±35.2	<0.0001	
	60-months	153.1±39.8	168.2±41.0	<0.0001	
	Close-out	158.2±40.6	174.4±42.2	<0.0001	
**Low-density lipoprotein cholesterol, mg/dL**	Baseline	104.5±38.7	108.3±38.7		<0.0001
	12-months	90.5±37.1	105.2±36.3	<0.0001	
	24-months	86.6±36.7	102.9±37.1	<0.0001	
	48-months	82.0±30.9	94.0±32.5	<0.0001	
	60-months	79.3±33.3	92.8±34.4	<0.0001	

© 2026 Leong DP et al. *JAMA Internal Medicine*.

	Close-out	83.9±34.0	98.2±35.6	<0.0001	
**High-density lipoprotein cholesterol, mg/dL**	Baseline	50.3±15.5	54.1±15.5		0.8
	12-months	51.0±13.9	51.8±14.7	0.18	
	24-months	51.0±14.3	51.4±15.5	0.67	
	48-months	53.0±15.5	52.2±13.1	0.54	
	60-months	51.0±13.5	51.0±14.3	0.92	
	Close-out	50.3±14.7	50.7±15.1	0.35	
**Triglycerides, mg/dL**	Baseline	150.6±88.6	150.6±88.6		0.03
	12-months	148.8±89.5	155.9±107.2	0.081	
	24-months	146.1±81.5	147.0±77.1	0.38	
	48-months	130.2±68.2	148.8±77.9	0.034	
	60-months	130.2±69.1	138.2±83.3	0.002	
	Close-out	134.6±74.4	144.4±105.4	0.20	
**Systolic blood pressure, mmHg**	Baseline	138.5±18.7	139.2±18.0		0.24

© 2026 Leong DP et al. *JAMA Internal Medicine*.

	12-months	133.9±16.8	136.5±18.1	0.001	
	24-months	133.3±15.9	136.4±18.5	0.0005	
	48-months	129.8±15.4	130.6±15.0	0.56	
	60-months	129.7±13.0	132.2±17.0	0.03	
	Close-out	131.1±16.9	132.9±18.3	0.03	
**Diastolic blood pressure, mmHg**	Baseline	82.9±11.1	83.3±11.1		0.02
	12-months	80.1±10.1	81.8±10.7	0.0004	
	24-months	78.3±10.8	81.1±15.7	<0.0001	
	48-months	76.6±9.1	77.5±9.9	0.35	
	60-months	77.3±9.5	77.9±11.1	0.40	
	Close-out	76.2±10.4	77.6±11.2	0.009	

© 2026 Leong DP et al. *JAMA Internal Medicine*.

eFigure 1. Clinical Event Cumulative Incidence Curves for Cardiovascular Death (CVD)

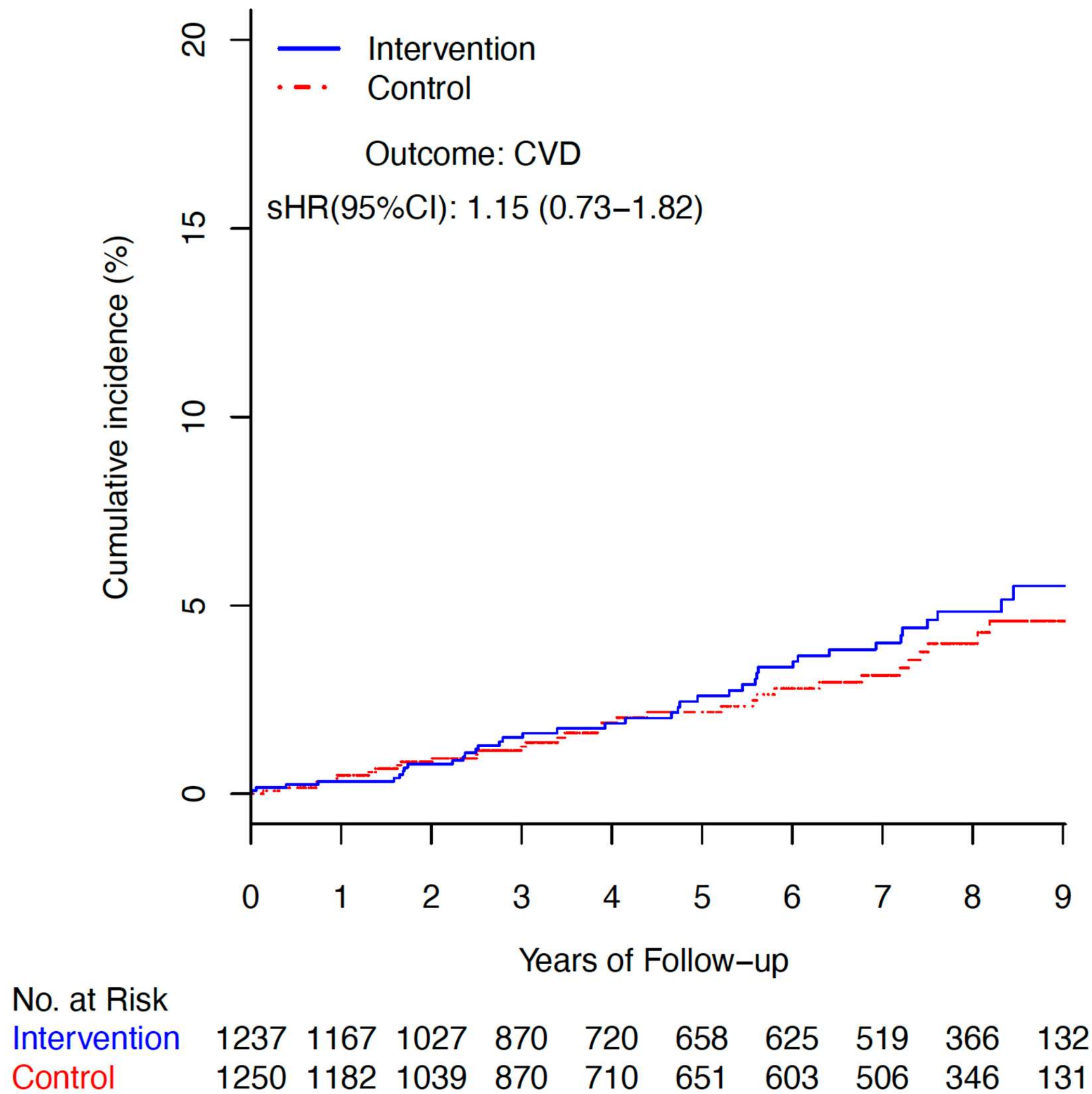

sHR = subdistribution hazard ratio

© 2026 Leong DP et al. *JAMA Internal Medicine*.

eFigure 2. Clinical Event Cumulative Incidence Curves for Myocardial Infarction (MI)

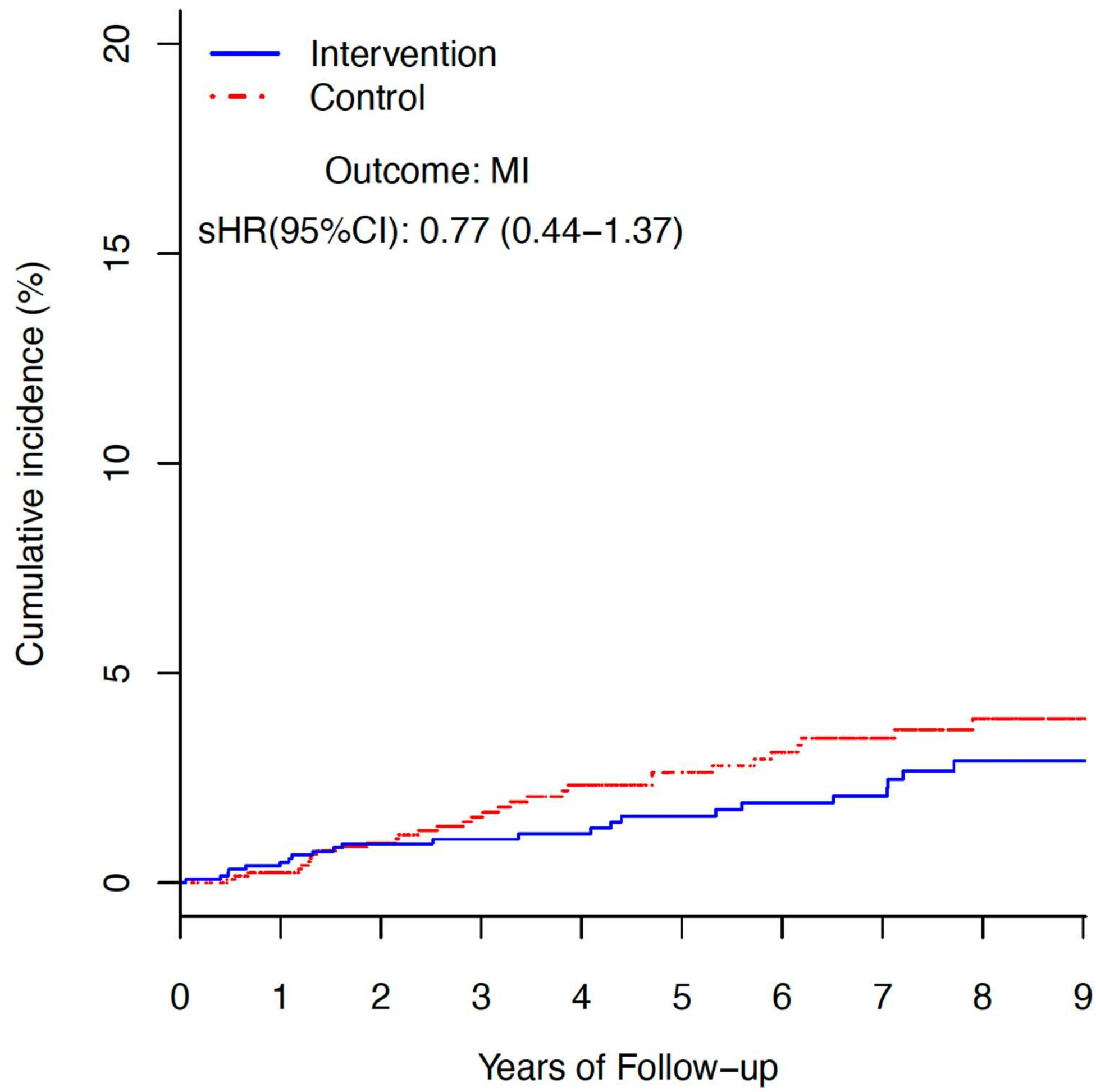

No. at Risk										
Intervention	1237	1162	1017	862	713	649	616	510	359	130
Control	1250	1179	1030	857	697	638	589	496	340	129

sHR = subdistribution hazard ratio

© 2026 Leong DP et al. *JAMA Internal Medicine*.

eFigure 3. Clinical Event Cumulative Incidence Curves for Stroke

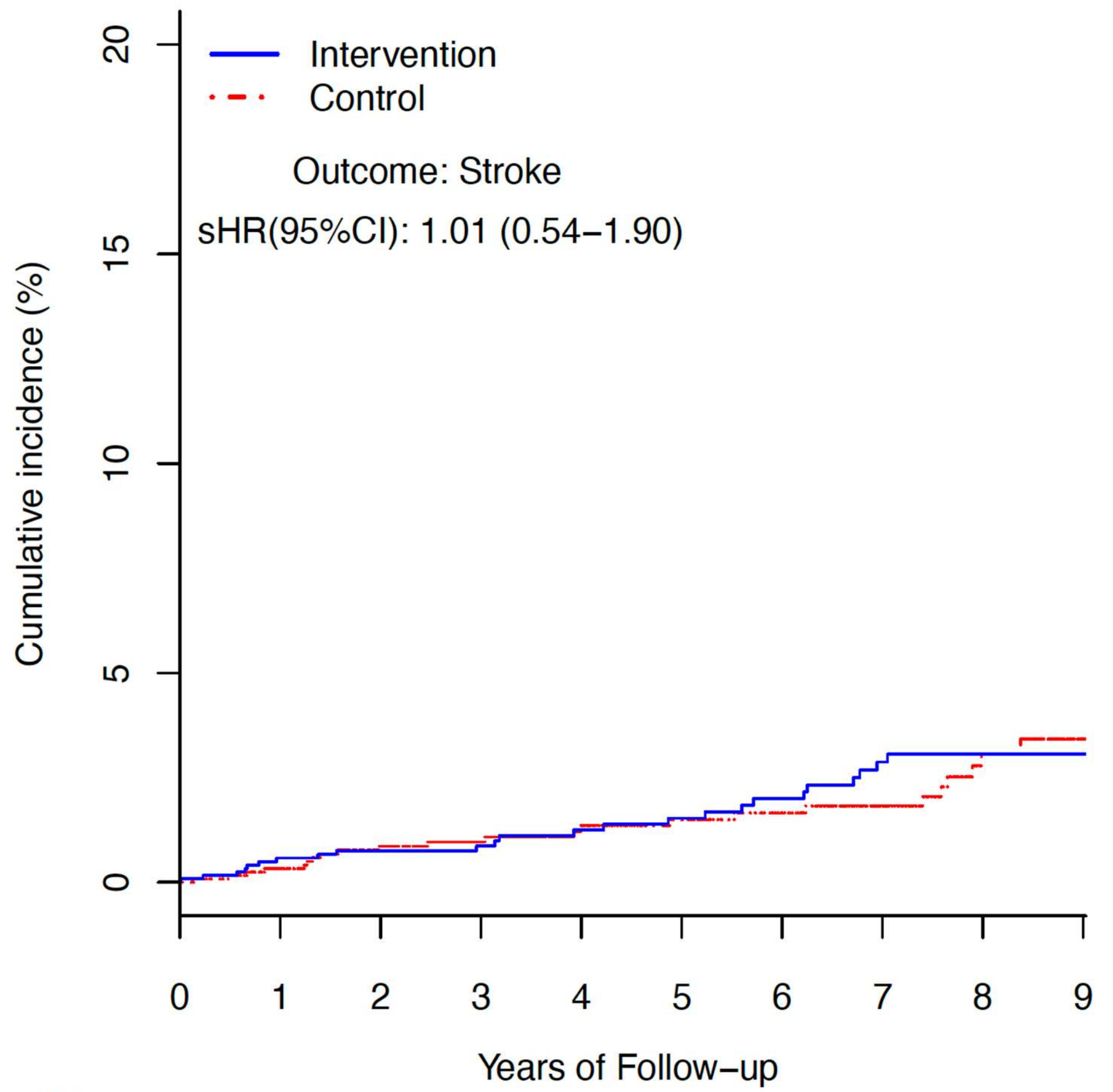

No. at Risk

Intervention	1237	1161	1023	865	713	651	617	507	358	130
Control	1250	1180	1034	868	706	646	598	500	340	130

sHR = subdistribution hazard ratio

© 2026 Leong DP et al. *JAMA Internal Medicine*.

eFigure 4. Clinical Event Cumulative Incidence Curves for Heart Failure (HF)

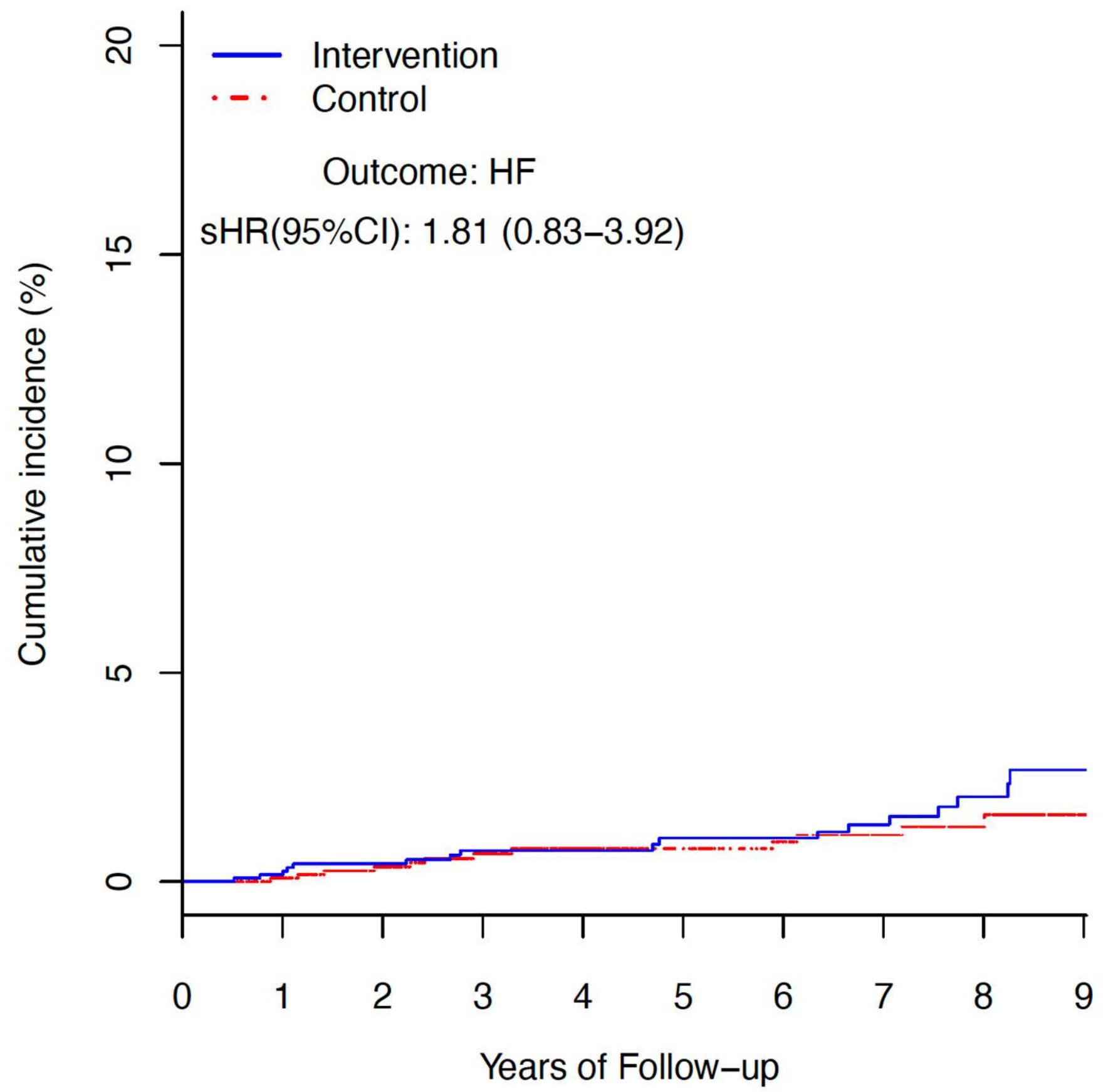

No. at Risk

Intervention	1237	1165	1024	867	719	656	623	516	361	129
Control	1250	1181	1036	864	707	649	600	502	344	131

sHR = subdistribution hazard ratio

© 2026 Leong DP et al. *JAMA Internal Medicine*.

eTable 2. Safety Outcomes

	**Overall N (%); rate per 100 person-years**	**Intervention N (%); rate per 100 person-years**	**Control N (%); rate per 100 person-years**	**Subdistribution hazard ratio (95% confidence interval), p-value**
Major Bleeding	79 (3.2); 0.6	44 (3.6); 0.7	35 (2.8); 0.5	1.48 (0.86-2.54), 0.16
Kidney Injury	74 (3.0); 0.6	40 (3.2); 0.6	34 (2.7); 0.5	1.11 (0.59-2.10), 0.74
Liver Injury	14 (0.6); 0.1	7 (0.6); 0.1	7 (0.6); 0.1	0.60 (0.14-2.56), 0.50
Myositis	0 (0.0); 0.0	0 (0.0); 0.0	0 (0.0); 0.0	Not applicable

© 2026 Leong DP et al. *JAMA Internal Medicine*.

eAppendix.

Funding/Support

RADICAL PC is an investigator-initiated study that is funded by Prostate Cancer Canada (Grant #CT2015-01), the Movember Foundation, Hamilton Health Sciences (RFA Research Strategic Initiative Program), Canadian Cancer Society and Canadian Institutes of Health Research (Grant # 706677), Population Health Research Institute, Hamilton Health Sciences Research Institute (HHSRI), Tolmar Pharmaceutiques Canada Inc.

RADICAL PC2 Committees, Investigators and Study Personnel

Steering Committee: Darryl P. Leong, Jehonathan H. Pinthus, P.J. Devereaux, Vincent Fradet, Laurence Klotz, Himu Lukka, Marina Mourtzakis, Joseph Selvanayagam, Avirup Guha, Celestia Higano, Alvaro Avezum, Ludhmila Hajjar

Adjudication Committee: Kelvin K.H. Ng (Chair), Pablo Lamellas, David Margel, Francois Caron, Malik Farooqi, Som Mukherjee, Germano Dallegrave Cavalli, Rocio Consuelo Baro Vila, Cristina Cano Garcia, Alexander Benz, Filipe Cirne

Data Safety Monitoring Board (DSMB): George Wells (Chair), Jean Rouleau, Michael L. Cher

Statisticians Reporting to DSMB: Kumar Balasubramanian

© 2026 Leong DP et al. *JAMA Internal Medicine*.

Study Statisticians & Programmers: Rajibul Mian, Olga Shestakovska, Bill Liu, Cissy Tang, Mark Molec, Nishitha Jeyachandradhas

ICT: Matthew Smart, Les Farago, Gianluca Rigitano, Miroslaw Malysz, Shrouk Saed, Nidhi Jethoo

Project Office Study Staff: Sumathy Rangarajan (Program Director), Sarah Karampatos, Steven Agapay, Peter Accardo, Amanda DeJesus, Maxwell Crispo, Rhonda Masney, Sabrina Woolgar, Ruchi Patel, Edith Wagan, Kayla Pohl, Steven Chen (Student Volunteer)

Cardio-Oncology Fellowship: Nazanin Aghel, Frances Carandang, Germano Dallegrave Cavalli, Filipe Cirne, Cristina Cano Garcia, Enrique Gonzalez, Harry Klimis, Jose Patricio Lopez-Lopez, Sophia Laird, Rocio Consuelo Baro Vila

Site Principal and Co-Investigators and Site Coordinators and staff:

Australia: Joseph B. Selvanayagam, Michael Chong, Matt Hong, Alex Jay, Antony So, Tonia Bromley, Briella Egberts, Amanda Weragoda; Tania Moujaber, Amy Hayden, Sandra Turner, Niluja Thiru, Sarah Zaman, Sindhu Mohanty, Hibah Harakeh, Anshu Kumar, Natasha Ouna, Niki Tashvighi Tehrani, Harieshan Chrishanthan, Bronte Coorey, Vanessa Eshou, April Purdon; Braden Higgs, Ross Roberts-Thomson, Hien Le, Phoebe Allison, Ryan J Appleton, Pooja Panchal, Peter Gorayski, Rachael Chang Lee, Ashwathy Mathew, Thomas Goode, Theodore Velkos, Emma Quinn; **Brazil:** Alexandra Vieira*, Felipe Martins*, Felipe Grabarz*, Adriadne J.

© 2026 Leong DP et al. *JAMA Internal Medicine*.

Bertolin*, Marcelly S. Rosal*, Marina Dannias*, Jose Leon*, Amanda Pedroso*, Nathalia L. T Santos*, Álvaro A. Avezum*, Dayane V. Nova*, Beatriz M. Sabino*, Rafael A. B. Nunes*, Precil D. M. M. Neves*, Daniela Maia*, Barbara Santana*, Giovana Nascimento*; Ludhmila Hajjar, Silvia Moulin, Edielle Melo, Beatriz Nascimento, Sabrina Carneiro; Ariel G. Kann, Emili G.M. Ayoub, Precil D. M. M. Neves, Rafael A. B. Nunes, Carlo C. Passerotti, José Pontes, Eliza D. Ricardo, Juliete J. Vidotti, Antonia L. L. Rodrigues, Nathalia L. T. Santos; Robert R. Odebrecht, Tércia S. Soares, Camila Camargo, Denise Sousa, Rogério D. Agatha, Maria A. Grapea, Juliana N. Paula, Antônio José Mata; Camila G. Ferrari, Fabio L. Couto Fernandez, Luiza A. B. Leite Ferreira, João A. Soler, Bruno C. M. Uchoa Junior, João Daniel C. Guedes, Kathia C. Abdalla, Ana Paula D. Martins, Katia Nishyama, Osvaldo L. da Silva Junior, Claudio Luis Tedokon, Denise Alexandra Santos, Isabelly C. M. Neves, Fabiola Martos, Fernanda Lima Paglione, Camila Costa Silva, Gabriela Taparo Castro, Bruna C. Marques, Beatriz P. Scarpim Luciana R. Cubas Volpe, Thamyres S. A. Cruz, Thiago B. Souza, Juliane M. Matos, Jaquline R. Mastro, Thamires W. L. Santana, Gabriela P. Bessa, Maria Fernanda Santos, Elaine B. Gallo, Lilian M. S. Pinho, Joelma I. Silva, João Pedro H. Netto; Mayra C. J. Frankenfeld, Ana L. Coradazzi, Claudia V. Cassaro; Felipe H. Valle, Estefania V. A. Andrade, Raquel O. Luiz, Fernanda R. Cunegatto, Taina dos S. Soares, Vera L. Oliveira-Freitas, Lais P. Lopes, Rafaela S. Da Silva, Laís P. Lopes, André B. Amon, Milene F. Sehn, Henrique I. Garbin, Andrei D. P. Rickrot, Marina N. Crispim; Lívia F.A. Oliveira, Giovani L. Santi, Lucinda C. Guimarães, Paulo H. M. Vilela, Paula M. Camasmie, Raíssa B. Luiz, Aline M. Carlos; Katsuki A. Tiscoski, Bruna B. Motke, Alessandra O. S. Santos, Daniela S. Santana, Luis E. S. M. Moz, Anita M. Maciel, Antonella Kaul, Gustavo M. Escott, Leticia M. S. Souza, Giovanna M. Barros, Guilherme L. Santos, Lorenzo S. Barcelos, Carolina P. Pereira, Patrícia E. C. Silva, Leonardo M. Santos,

© 2026 Leong DP et al. *JAMA Internal Medicine*.

Tatiane M. R. Reis, Lavinia P. Carvalho, Lauren M. Xavier, Camila G. Soares, Celina F Silva,, Vera L. O. Freitas; Lin I. Ter, Roni C. Fernandes, Luana C. F. F. Gelschyn, Hamanda Oliveira, Laise M. M. de Sousa, Alessandra O. dos Santos, Daniela de Santana, Luis Eduardo S Móz,; Eduardo Schlabendorff, Vanessa S. dos Santos, Ana L. Abrahao, Cristine Erdmann Nunes, Carollainy Nascimento Silva, Aline Gonçalves Borba, Amanda Fernandes Barbosa, Gabriela Petry Crestani, Cristiano Pederneiras Jaeger, Karen Brasil Ruschel; Murillo O. Antunes, Simone Felitti, Mariana R. Gonçalves, Julia V. Matos, Karollyny A. Salgueiro, Joice C. Couto, Laura D. Cassab; Rafaela K. Piccoli, Leticia Y. Castro, Edilson Walter, Bianca A. B. Kuhn, Gabriela Strucker, Gabrielly M. Ribeiro, Juliana S. Pereira, Maria E M Siqueira, Fernanda B. Uggeri, Adriana N M Basílio, Giovanna Uehara, Alayne M T D Yamada, Graziele O Santos, Amanda Panisson, Francini O. Rodrigues, Roberta Cattaneo; Cristiano F. Souza, Manoella Gomes, Gabriel R. Figueiredo, Amanda Noveline, Michelle P. Silva, Lenilda A. M. Santos, Rosangela S. Costa, Marilia F. Nascimento, Marina Leonardo, Giulia O. Toffolo, Nayane Marangoni; Patricia M. M. Beato, Thales G. May, Brigida Ranzani, Natalia L. Rodrigues, Felipe A. A. Carboni, Adriana B. Coutinho, Luana D. Vieira, Gustavo S. Santos, Debora C. Campos, Guilherme R. Agostinho, Franciele A. C. Silva, Hellen C. G. Costa, Mariana G. Cardoso, Maria G. E. Chiachio, Elaine P. O. Rosa, Erika V. M. Silva; Livia A. Pereira, Renata P. Costa, Thais P. Ribeiro, Maria J. A. Tostes, Luanna L. F. Gonçalves; Adriano Caixeta, Paula Teixeira, Joyce Yamamoto; Carlos A. K. Nakashima, Dalton B. Precoma, Lilian Belinaso, Camila Richter, Cristina P. Correa, Mayara A. Barbosa, Eliane Sehnem, Maynara L. S. Martins, Meire E. Yokota, Karina S. Krasinski, Méllanie A. S. Ferreira, Ketlyn C. P. Silva, Ariella P. B. Bongiorno,; Gustavo L. Marques, Rafael M. Miyazima; Jurema T. Lima, Andrea L. Souza, Henrique Mota, Mateus C. T. Feitosa, Carla R. A. S. Azevedo, Eduarda L. S. Silva, Nathalia S.

© 2026 Leong DP et al. *JAMA Internal Medicine*.

B. Silva, Laíse M. X. Silva, Maria I. Canejo, Danielle M. Vasconcelos, Júlia M. S. S. Santiago; João Borges, Luciana de Avila, Beatriz Oliveira, Maria Gomes, Maria Freire, Luana Alves, Maria Costa; AF. Issa, M.R. Faria, TA. Marinho, Fabiola L. Aragao, TC. Guimaraes, LC. Fialho, MR Faria, FL Aragão, PA Leite; Marcio Kawano, Maristela Miranda, Fernando Alfieri, Manfredo K. Naritomi, Walquiria C. R. Ritter; Cesar Dusilek, Brenno Vidotti, Diego Maia, Gabrielly Lech; Paulo Tierno, Bianca Bocalon, Stefany Robis, Ana Paula Araujo, Oliveira Milena; Fabio R. Oliveira, Karen B. Ribeiro; **Canada:** Jehonathan Pinthus, Darryl P. Leong, Nazanin Aghel, Aly-Khan Lalani, Himanshu Lukka, Filipe Cirne, Shauna Szendrey, Nadra Yasmin, Meghan Cagnin, Maheen Farooqi, Aaron Donst, Ahmed Osman, Thomas Corbett, Ian Dayes, Pierre Major, Kimmen Quan, Mira Goldberg, Susan Gudelis, Theo Tsakiridis, Sebastian Hotte, Som Mukherjee, Harry Klimis, Muhammad Mustafa Alhussein, Enrique Gonzalez Nava, Alanna Hall, Amanda Thiessen; Bobby Shayegan, Vikas Tandon, Edward D. Matsumoto, Anil Kapoor#, Douglas S. Wright, David Conen, Vaibhav Chudasama, Mona Mirshahi, Aishwarya Sudakaran, Sarah Karampatos, Dalia Elhadidy, Laurence Klotz, Idan Roifman, Marlene Kebabdjian, Jennifer Toopitsin, Shania Nigli, Betsy Klotz, Lara Lotz, Robert Siemens, Raveen Pal, Arnon Lavi, Ashley Griffin, Spencer Craig, Jennifer Lalonde, Stephen Mardell, Angela Black, Patrick Luke, Marko Mrkobrada, Alp Sener, Andrew Appleton, Cadence Baker, Kate Robertson-Hogg, Kate Robertson-Hogg, Taylor Toth, Mounirah May, Mohammad Tamim Niazi, Annabel Chen-Tournoux, Kelli Dimayuga, Pressila Njeim; Paola Diego, Melanie Criqui, Rita Su, Wasim Niazi, Thérèse-Marie Poinsignon, Vincent Fradet, Yves Fradet, Valerie Gaudreault, Nancy Filion, William Foster, Audrey Larouche, Josée Allard, Denise St-Onge, Carole Plante, Joanie Deroy-Lambert, Alexandra Pelletier, Marie-Andrée Lajoie, Sophie Pouliot, Gentiane Rajaobelison, Michele Lodde, Louis Lacombe, Paul Toren, Éric Vigneault, Marie-Anne

© 2026 Leong DP et al. *JAMA Internal Medicine*.

Froment, André-Guy Martin, Yves Caumartin, Thierry Dujardin, Frédéric Pouliot, Étienne Lavallée, Arnaud Marien, Catherine Gerard, Emmanuelle Duceppe, Maroie Barkati, Marie-Claude Beauchemin, Guila Delouya, Madeleine Durand, Carole Lambert, Cynthia Ménard, Fred Saad, Daniel Taussky, Jean-Baptiste Lattouf, Melodie F. Boko, Imène Chergui, Elliott Deligne, Roberta Daila Carling, Dominique Matthys, Catherine Lapointe, Diane Baptiste, Eva Amzallag, Manel Rezgui, Megan Faye Parker, Myriam Hamtiaux; Ève Comeau, Amélie Martin, Luke T. Lavallée, Rodney H. Breau, Ilias Cagiannos, Christopher Johnson, Christopher Morash, Marika Mihok, David Yachnin, Jeffrey Warren; Negareh Mousavi, Amal Bessissow, Bersabeh A. Hailemariam; Robert Hamilton, Husam Abdel-Qadir, Antonio Finelli, Nathan Perlis, Neil Fleshner, Peter Chung, Alejandro Berlin, Aaron Hansen, Najia Khurram, Zacharia Burrafato, Rajeswari Aravindan; Patrick Anderson, Ian Brown, Abhirami Hallock, Adnan Hameed, Marian Kotrec, Alexandra Bojcevski, Shraddha Sawant, Peter Accardo, David Cuthbert, Adrian Ishkanian, Theorodos Tsakiridis,; Melissa J. Huynh, Joseph Chin, George Dresser, Nidhi Shah, Corrine Hickling; Darindra Gopaul, Daniel Glick, Joda Kuk, Anthony Wassef, Jason Ding, Stephanie Thompson, Carol Ballantyne,Elyse Wellhauser, Stephanie O'Brien, Stephanie Nemirov; Philippe D. Violette, Rachel E. Gutschon, Dana L. Martin; Margot Davis, Celestia S. Higano, Michael Luong, Lynn Straatman#, Sean Virani, Brian Clarke, Scott Tyldesley, Jaclyn Chow, Himantha De Silva, Rosalie SF. Ho, Maria Daniella Sare, Abigail C. O'Connor, Monita R. Sundar, Abigail C. O'Connor,; Nawar Hanna, Audrey Lamoureux, Anabel Grullon, Robert Sabbagh, Claudio Jeldres, Carmel Michel, Richard Patrick, Samuel Lagabrielle, Warner Mampuya, Patricia Pomerleau, Nathalie Goudreau, Isabelle Tessier, Sandra Groulx, Shawn Byrns-Corbeil, Andrée-Anne Turcotte; Jessica Bou Abdallah, Diana Kaddour, Marie-Pier Lessard; **Colombia:** Marisol López*, Jose Patricio López*; Nicolas Villareal, Boris E. Vesga,

© 2026 Leong DP et al. *JAMA Internal Medicine*.

Maria Alejandra Solano Garcia, Luz J. Carvajal, Jesus D. Polo, Sandra M. Rodriguez, Valentina Ortegón, Daniela Castillo, Nathalia K. Amado, Laura Celis, Mayra L. Arciniegas, Francy Gutierrez, Laura F. Neira, Karina B. Mejia, yudy A. Gamboa, Claudia L. Angarita; José E. Cita, Paola A. Pinilla, Jorge O. Reyes, Sergio A. Prado, Angel A. Garcia, Viviana M. Mendez, Katherine Castrillon; Juan E. Gomez, Sara N. Burbano, Carlos E. Vesga, Diana C. Carrillo, Brayan D. Cordoba, Pablo Sierra, Lady T. Delgado, Diana M. Rangel, Diana L. Mora, Gloria I. Jimenez, Laura A. Trujillo, Harrison A. Vargas, Ana L. Cuaran; **India:** Denis Xavier*, Deepa Srinivasan*, Freeda Xavier*, Preeti Girish*, Anthea M. Rayan*, Aakanksha Singh*; Lalit Mohan Sharma, Ajay Yadav, Rounak Vaishnav, Aaisha Kazi; Srivatsa Narasimha, C B Patil, Ranjitha Karanth, John Chungath, Shobha Chandrashekaraiah, Manila Manjunath, Somanagouda Biradar; Hari Menon, Ganesha Vashishta, Annie Kanchan Baa, Avinash HU, Mohan A, Akshay L, Clera Lewis, Francis Baby; Surya P. Vaddi, Likhiteswer Pallagani, Ravikanth Athuluri, Shilpa Palla, Surya P. Vaddi; **Israel:** A.E. Eisen, N.O. Oginets; David Sarid, Daniel Keizman, Marina Liberzon, Limor Ben-Zvi, Sapir Amitay, Aya Mazarib, Marganit Shahar, Elinor Harel; **Singapore:** Chieh Yang Christopher Koo, Li Ling Tan, Edmund Chiong, Arshvin Kesavan, Zhijiang Zang, Woon Chau Tsang, Seng Cheong Alvin Wong, Jolene Leong, Shu Yi Tan, Li Xiang Felicia Ong; **United States:** Charles Porter, Elizabeth Wulff, Brian Weiford, Saqib Abbasi, Rahul Parikh, Jennifer Heins, Hamed Dehkordi, Terry Christsenson, Amy L. Gorsuch, Kartik Munshi, Ankita Tiwari.

*National Coordinator

[#] Deceased

© 2026 Leong DP et al. *JAMA Internal Medicine*.

RADICAL PC2 Institution Names

Country	Institution
Australia	Flinders Medical Centre<br>Westmead Hospital<br>Royal Adelaide Hospital
Brazil	Instituto do Coração (INCOR)<br>Hospital Alemao Oswaldo Cruz (HAOC)<br>Hospital Santa Marcelina<br>Fundação Faculdade de Medicina de São José do Rio Preto<br>Faculdade de Medicina da Universidade Estadual Paulista – UNESP<br>Hospital de Clínicas de Porto Alegre<br>Universidade Federal do Triângulo Mineiro<br>Irmandade Da Santa Casa De Misericórdia De Porto Alegre<br>Santa Casa de São Paulo (IPITEC)<br>Hospital Mae de Deus<br>Hospital Universitário São Francisco na Providência de Deus<br>Hospital de Caridade de Ijuí<br>Núcleo de Pesquisa e Ensino da Rede São Camilo<br>Fundação Doutor Amaral Carvalho<br>Hospital Felício Rocho<br>Universidade Federal de São Paulo (UNIFESP)

© 2026 Leong DP et al. *JAMA Internal Medicine*.

	Sociedade Hospitalar Angelina Caron<br>Centro Médico São Francisco<br>Instituto de Medicina Integral Professor Fernando Figueira (IMIP)<br>Centro de Pesquisa Clínica do Brasil<br>Instituto Nacional De Cardiologia<br>Hospital Nipo-Brasileiro<br>Hospital do Rocio<br>Hospital Municipal de Barueri
Canada	Juravinski Cancer Centre<br>St. Joseph's Healthcare<br>Sunnybrook Health Sciences Centre<br>Queen's University<br>London Health Sciences Centre<br>Jewish General Hospital<br>CHU de Québec - Université Laval<br>Centre Hospitalier de l'Université de Montréal (CHUM)<br>Ottawa Hospital Research Institute and University of Ottawa<br>McGill University Health Centre (MUHC)<br>University Health Network / Princess Margaret Cancer Centre<br>Niagara Health System / Walker Family Cancer Center<br>London Health Sciences Centre (Victoria Hospital)<br>Waterloo Regional Health Network

© 2026 Leong DP et al. *JAMA Internal Medicine*.

	Woodstock Hospital<br>University of British Columbia - Center for Cardiovascular Innovatoin / Prostate Supportive Care Program<br>Hopital Maisonneuve-Rosemont / CIUSSS de l'Est de l'ile de Montreal<br>Centre Hospitalier Universaitaire de Sherbrooke
Colombia	Fundación Oftalmologica de santander - FOSCAL<br>Hospital Universitario San Ignacio<br>Fundación Valle del Lili
India	Mahatma Gandhi University of Medical Sciences & Technology<br>Sri Shankara Cancer Hospital and Research Centre<br>St. John's Medical College and Hospital<br>Kamineni Academy of Medical Sciences and Research Centre
Israel	Rabin Medical Center<br>Tel-Aviv Sourasky Medical Center
Singapore	National University Hospital Singapore
United States	The University of Kansas Medical Center

© 2026 Leong DP et al. *JAMA Internal Medicine*.
