# Supplementary material for: Specialist Referral for Cardiovascular Risk in Patients With Prostate Cancer: A Randomized Clinical Trial
Source: JAMA Intern Med. 2026 Aug 30:e264773. Online ahead of print. doi: 10.1001/jamainternmed.2026.4773 (PMC13527639; doi:10.1001/jamainternmed.2026.4773)
Supplement: Supplementary file 4 [file jamainternmed-e264773-s004.pdf]

Supplemental Online Content: Nonauthor Collaborators

*First name, last name, and suffix (if applicable) are required and will appear in PubMed.

***Group Name(s): RADICAL PC Project Office Staff, National Coordinators, Investigators, and Key Staff**

*First Name and Middle Initial(s)	*Last Name	*Suffix (eg, Jr, III)	Academic Degrees	Institution	Location (city, state/province, country)	Role or Contribution (eg, chair, principal investigator, co-investigator, study coordinator)	Group (if more than 1 Group listed in the byline) and/or Subgroup (eg, Steering Committee)
Alanna	Hall		RRT	Hamilton Health Sciences	Hamilton, Canada	study coordinator	
Meghan	Cagnin		BSc	Hamilton Health Sciences	Hamilton, Canada	study coordinator	
Maheen	Farooqi		BSC	Hamilton Health Sciences	Hamilton, Canada	study coordinator	
Aaron	Donst		BSc, MSc, PCP	Hamilton Health Sciences	Hamilton, Canada	study coordinator	
Amanda	Thiessen		PCP	Hamilton Health Sciences	Hamilton, Canada	study coordinator	
Shauna	Szendrey		BSc	Hamilton Health Sciences	Hamilton, Canada	study coordinator	
Nadra	Yasmin		MBBS	Hamilton Health Sciences	Hamilton, Canada	study coordinator	
Aly-Khan	Lalani		MD, FRCPC	Hamilton Health Sciences / McMaster University	Hamilton, Canada	Co-Investigator	
Nazanin	Aghel		MD	Hamilton Health Sciences / McMaster University	Hamilton, Canada	Co-Investigator	
Thomas	Corbett		MD	Hamilton Health Sciences / McMaster University	Hamilton, Canada	Collaborator	
Ian	Dayes		MD, MSc	Hamilton Health Sciences / McMaster University	Hamilton, Canada	Collaborator	
Pierre	Major		MD	Hamilton Health Sciences / McMaster University	Hamilton, Canada	Collaborator	
Kimmen	Quan		MD	Hamilton Health Sciences / McMaster University	Hamilton, Canada	Collaborator	
Mira	Goldberg		MD	Hamilton Health Sciences / McMaster University	Hamilton, Canada	Collaborator	
Susan	Gudelis		MD	Hamilton Health Sciences / McMaster University	Hamilton, Canada	Collaborator	
Theo	Tsakiridis		MD, PhD	Hamilton Health Sciences / McMaster University	Hamilton, Canada	Collaborator	
Sebastian	Hotte		MD, MSc	Hamilton Health Sciences / McMaster University	Hamilton, Canada	Collaborator	
Som	Mukherjee		MD, MSc, FRCP	Hamilton Health Sciences / McMaster University	Hamilton, Canada	Collaborator	
Muhammad	Mustafa Alhussein		MD	Hamilton Health Sciences / McMaster University	Hamilton, Canada	Collaborator	
Peter	Accardo		MSc	Hamilton Health Sciences / St. Joseph's Healthcare Ham	Hamilton, Ontario, Canada	study coordinator	
Ahmed	Osman		MScPH	McMaster University	Hamilton, Ontario, Canada	study coordinator	
Shraddha	Sawant		MBBS, MPH	Hamilton Health Sciences/St. Joseph's Healthcare Hamil	Hamilton, Ontario, Canada	study coordinator	
Vaibhav	Chudasama		MSc	Hamilton Health Sciences/St. Joseph's Healthcare Hamil	Hamilton, Ontario, Canada	study coordinator	
Mona	Mirshahi		MSc	Hamilton Health Sciences/St. Joseph's Healthcare Hamil	Hamilton, Ontario, Canada	study coordinator	
Aishwarya	Sudakaran		B.Tech	Hamilton Health Sciences/St. Joseph's Healthcare Hamil	Hamilton, Ontario, Canada	study coordinator	
David	Conen		MD	St. Joseph's Healthcare Hamilton/Hamilton Health Scien	Hamilton, Ontario, Canada	Co-Investigator	
Douglas S.	Wright		MD	St. Joseph's Healthcare Hamilton/Hamilton Health Scien	Hamilton, Ontario, Canada	Co-Investigator	
Edward D.	Matsumoto		MD	St. Joseph's Healthcare Hamilton/McMaster University	Hamilton, Ontario, Canada	Co-Investigator	
Vikas	Tandon		MD	St. Joseph's Healthcare Hamilton/McMaster University	Hamilton, Ontario, Canada	Co-Investigator	
Idan	Roifman		MD MSc FRCPC	Sunnybrook Health Sciences Centre	Toronto, Ontario, Canada	Co-Investigator	
Shania	Nigli			Sunnybrook Health Sciences Centre	Toronto, Ontario, Canada	study coordinator	
Craig	Spencer			Queen's University	Kingston ON	study coordinator	Centre for Applied Urological Research
Jennifer	Lalonde		BScN	Queen's University	Kingston, Ontario	study coordinator	
Ashley	Griffin			Queen's University	Kingston, Ontario	study coordinator	
Cadence	Baker		BSc, MSc	London Health Sciences Centre	London, Ontario, Canada	Research Manager	
Kate	Robertson-Hogg		BScN	London Health Sciences Centre	London, Ontario, Canada	Study Assistant	
Kelli	Dimayuga		Bsc	Concordia University	Montreal, Canada	study coordinator	
Annabel	Chen-Tournoux		MD	Jewish General Hospital	Montreal, Quebec, Canada	Sub-I	
Paola	Diego		BScN	Jewish General Hospital	Montreal, Quebec, Canada	Study Nurse	
Melanie	Criqui		PhD	Jewish General Hospital	Montreal, Quebec, Canada	Study Coordinator (ethics)	
Rita	Su		MSc	Jewish General Hospital	Montreal, Quebec, Canada	Data Entry	
Wasim	Niazi		DEC	Jewish General Hospital	Montreal, Quebec, Canada	study coordinator	
Pressila	Njeim		MSc	Jewish General Hospital	Montreal, Quebec, Canada	study coordinator	
Thérèse-Marie	Poinsignon		MSc	Jewish General Hospital	Montreal, Quebec, Canada	study coordinator	
Valerie	Gaudreault		MD, cardiologist	CHU de Québec - Université Laval	Québec, Qc, Canada	Co-Investigator	
Filion	Nancy		MD, FRCSC	CHU de Québec - Université Laval	Québec, Qc, Canada	Co-Investigator	
Michele	Lodde		MD,urologist-oncologis	CHU de Québec - Université Laval	Québec, Qc, Canada	Co-Investigator	
William	Foster		MD, rad-oncologist	CHU de Québec - Université Laval	Québec, Qc, Canada	Co-Investigator	
Yves	Fradet		MD,urologist-oncologis	CHU de Québec - Université Laval	Québec, Qc, Canada	Co-Investigator	
Louis	Lacombe		MD,urologist-oncologis	CHU de Québec - Université Laval	Québec, Qc, Canada	Co-Investigator	
Paul	Toren		MD, PhD, urologist-onc	CHU de Québec - Université Laval	Québec, Qc, Canada	Co-Investigator	
Éric	Vigneault		MD, rad-oncologist	CHU de Québec - Université Laval	Québec, Qc, Canada	Co-Investigator	
Marie-Anne	Froment		MD, rad-oncologist	CHU de Québec - Université Laval	Québec, Qc, Canada	Co-Investigator	
André-Guy	Martin		MD, rad-oncologist	CHU de Québec - Université Laval	Québec, Qc, Canada	Co-Investigator	
Audrey	Larouche		RN	CHU de Québec - Université Laval	Québec, Qc, Canada	study coordinator	
Josée	Allard		RN	CHU de Québec - Université Laval	Québec, Qc, Canada	study coordinator	
Denise	St-Onge		Msc, research assistant	CHU de Québec - Université Laval	Québec, Qc, Canada	study coordinator	
Carole	Plante		RN	CHU de Québec - Université Laval	Québec, Qc, Canada	study coordinator	
Yves	Caumartin		MD,urologist	CHU de Québec - Université Laval	Québec, Qc, Canada	co-investigator	
Thierry	Dujardin		MD,urologist	CHU de Québec - Université Laval	Québec, Qc, Canada	co-investigator	
Frédéric	Pouliot		MD,urologist-oncologis	CHU de Québec - Université Laval	Québec, Qc, Canada	co-investigator	
Étienne	Lavallée		MD,urologist-oncologis	CHU de Québec - Université Laval	Québec, Qc, Canada	co-investigator	
Arnaud	Marien		MD, urologist-oncologi	CHU de Québec - Université Laval	Québec, Qc, Canada	co-investigator	
Joanie	Deroy-Lambert		RN	CHU de Québec - Université Laval	Québec, Qc, Canada	study coordinator	
Alexandra	Pelletier		RN	CHU de Québec - Université Laval	Québec, Qc, Canada	study coordinator	
Gentiane	Rajaobelison		RN	CHU de Québec - Université Laval	Québec, Qc, Canada	reg coordinator	
Sophie	Pouliot		RN	CHU de Québec - Université Laval	Québec, Qc, Canada	study coordinator	
Carole	Lambert		MD MSc	Centre Hospitalier de l'Université de Montréal (CHUM)	Montreal, Quebec, Canada	Co-investigator	
Cynthia	Ménard		MD	Centre Hospitalier de l'Université de Montréal (CHUM)	Montreal, Quebec, Canada	Co-investigator	
Daniel	Taussky		MD	Centre Hospitalier de l'Université de Montréal (CHUM)	Montreal, Quebec, Canada	Co-investigator	
Maroie	Barkati		MD	Centre Hospitalier de l'Université de Montréal (CHUM)	Montreal, Quebec, Canada	Co-investigator	
Fred	Saad		MD	Centre Hospitalier de l'Université de Montréal (CHUM)	Montreal, Quebec, Canada	Co-investigator	
Guila	Delouya		MD MSc	Centre Hospitalier de l'Université de Montréal (CHUM)	Montreal, Quebec, Canada	Co-investigator	
Madeleine	Durand		MD MSc	Centre Hospitalier de l'Université de Montréal (CHUM)	Montreal, Quebec, Canada	Co-investigator	
Marie-Claude	Beauchemin		MD	Centre Hospitalier de l'Université de Montréal (CHUM)	Montreal, Quebec, Canada	Co-investigator	
Jean-Baptiste	Lattouf		MD	Centre Hospitalier de l'Université de Montréal (CHUM)	Montreal, Quebec, Canada	Co-investigator	

Melodie F	Boko	MSc	Centre Hospitalier de l'Université de Montréal (CHUM	Montreal, Quebec, Canada	Study Coordinator	
Elliott	Deligne	BSc	Centre Hospitalier de l'Université de Montréal (CHUM	Montreal, Quebec, Canada	Study Coordinator	
Imène	Chergui	MD	Centre Hospitalier de l'Université de Montréal (CHUM	Montreal, Quebec, Canada	Study Coordinator	
Roberta	Daila Carling	MSc	Centre Hospitalier de l'Université de Montréal (CHUM	Montreal, Quebec, Canada	Study Coordinator	
Dominique	Matthys	MSc	Centre Hospitalier de l'Université de Montréal (CHUM	Montreal, Quebec, Canada	Study Coordinator	
Catherine	Lapointe	MSc	Centre Hospitalier de l'Université de Montréal (CHUM	Montreal, Quebec, Canada	Study Coordinator	
Diane	Baptiste	MSc	Centre Hospitalier de l'Université de Montréal (CHUM	Montreal, Quebec, Canada	Study Coordinator	
Eva	Amzallag	MSc	Centre Hospitalier de l'Université de Montréal (CHUM	Montreal, Quebec, Canada	Study Coordinator	
Manel	Rezgui	MSc	Centre Hospitalier de l'Université de Montréal (CHUM	Montreal, Quebec, Canada	Study Coordinator	
Ève	Comeau	MSc	Centre Hospitalier de l'Université de Montréal (CHUM	Montreal, Quebec, Canada	Study Coordinator	
Megan	Faye Parker	BSc	Centre Hospitalier de l'Université de Montréal (CHUM	Montreal, Quebec, Canada	Study Coordinator	
Myriam	Hamtiaux	BSc	Centre Hospitalier de l'Université de Montréal (CHUM	Montreal, Quebec, Canada	Study Coordinator	
Amélie	Martin	M.P.O	Centre Hospitalier de l'Université de Montréal (CHUM	Montreal, Quebec, Canada	Study Coordinator	
Christopher	Johnson	MD, FRCP	Ottawa Hospital Research Institute and University of Ottawa	Ottawa, Ontario, Canada	Co-Investigator	
David	Yachnin	BSc, MSc	Ottawa Hospital Research Institute and University of Ottawa	Ottawa, Ontario, Canada	Coordinator	
Marika	Mihok	MD, FRCP	Ottawa Hospital Research Institute and University of Ottawa	Ottawa, Ontario, Canada	Co-Investigator	
Rodney H.	Breau	MD, MSc, BSc, FRCSC	Ottawa Hospital Research Institute and University of Ottawa	Ottawa, Ontario, Canada	Co-Investigator	
Ilias	Cagiannos	MD, BSc, FRCSC	Ottawa Hospital Research Institute and University of Ottawa	Ottawa, Ontario, Canada	Co-Investigator	
Jeffrey	Warren	MD, BSc, FRCSC	Ottawa Hospital Research Institute and University of Ottawa	Ottawa, Ontario, Canada	Co-Investigator	
Christopher	Morash	MD, BSc, FRCSC	Ottawa Hospital Research Institute and University of Ottawa	Ottawa, Ontario, Canada	Co-Investigator	
Negareh	Mousavi	M.D.	McGill University Health Centre (MUHC)	Montreal, Quebec, Canada	Principal Investigator	
Amal	Bessissow	M.D., M.Sc., FRCPC	McGill University Health Centre (MUHC)	Montreal, Quebec, Canada	Co-investigator	
Bersabeh A.	Hailemariam	B.Sc	McGill University Health Centre (MUHC)	Montreal, Quebec, Canada	Study Coordinator	
Robert	Hamilton	MD, MPH	University Health Network	Toronto, ON, Canada	Principal Investigator	
Alejandro	Berlin	MD, MSc	University Health Network	Toronto, ON, Canada	Co-Investigator	
Husam	Abdel-Qadir	MD, FRCPC, PhD, DA	University Health Network	Toronto, ON, Canada	Co-Investigator	
Peter	Chung	MBChB, MRCP, FRCI	University Health Network	Toronto, ON, Canada	Co-Investigator	
Antonio	Finelli	MD, MSc, FRCSC	University Health Network	Toronto, ON, Canada	Co-Investigator	
Neil	Fleshner	MD, MPH	University Health Network	Toronto, ON, Canada	Co-Investigator	
Aaron	Hansen	MBBS, FRACP	University Health Network	Toronto, ON, Canada	Co-Investigator	
Nathan	Perlis	MD, MSc, FRCSC	University Health Network	Toronto, ON, Canada	Co-Investigator	
Najia	Khurram	MBBS, ECFMG, CCRI	University Health Network	Toronto, ON, Canada	Research Coordinator	
Zacharia	Burrafato	BSc	University Health Network	Toronto, ON, Canada	Research Coordinator	
Rajeswari	Aravindan		University Health Network	Toronto, ON, Canada	Data coordinator	
Alexandra	Bojcevski	MD, FRCPC	Cardiology Associates of Niagara, Niagara Health St. Ca	St. Catharines and Thorold, Ontario, Canac	Co-Investigator	
Adnan	Hameed	BSc, MD, FRCPC	Cardiology Associates of Niagara, Niagara Health St. Ca	St. Catharines and Thorold, Ontario, Canac	Co-Investigator	
Marian	Kotrec	MD, FRCPC	Cardiology Associates of Niagara, Niagara Health St. Ca	St. Catharines and Thorold, Ontario, Canac	Co-Investigator	
Ian	Brown	MD, FRCS (C) Urolog	Niagara Health St Catharines, Canadian Urological Asso	St. Catharines, Ontario, Canada	Principal Investigator	
Abhirami	Hallock	MD	Niagara Health St Catharines, Canadian Urological Asso	St. Catharines, Ontario, Canada	Co-Investigator	
David	Cuthbert	MD	Walker Family Cancer Center, St Catharines	St. Catharines, Ontario, Canada	Collaborator	
Adrian	Ishkanian	MD	Walker Family Cancer Center, St Catharines	St. Catharines, Ontario, Canada	Collaborator	
Theorodos	Tsakiridis	MD	Walker Family Cancer Center, St Catharines	St. Catharines, Ontario, Canada	Collaborator	
Melissa	Huynh	MD	London Health Sciences Centre	London, Ontario, Canada	Principal Investigator	
Joseph	Chin	MD	London Health Sciences Centre	London, Ontario, Canada	Principal Investigator	
George	Dresser	MD	London Health Sciences Centre	London, Ontario, Canada	Co-Investigator	
Arnon	Lavi	MD	London Health Sciences Centre	London, Ontario, Canada	Co-Investigator	
Nidhi	Shah	BSc, MSc	London Health Sciences Centre	London, Ontario, Canada	Study Coordinator	
Corrine	Hickling	BSc	London Health Sciences Centre	London, Ontario, Canada	Study Coordinator	
Stephen	Mardell	BSc	London Health Sciences Centre	London, Ontario, Canada	Study Coordinator	
Raveen	Pal	MD	Queen's University/Kingston Health Sciences Centre	Kingston ON	Co-Investigator	Dept of Cardiology
Melissa J	Huynh	MD, MPH	Western University			
Darindra	Gopaul	MD	Waterloo Regional Health Network	Kitchener, Ontario, Canada	Principal Investigator	
Joda	Kuk	BSc, MD, FRCPC	Waterloo Regional Health Network	Kitchener, Ontario, Canada	Co-Investigator	
Anthony	Wassef	MD, MSc, FRCPC	Waterloo Regional Health Network	Kitchener, Ontario, Canada	Co-Investigator	
Daniel	Glick	MD	Waterloo Regional Health Network	Kitchener, Ontario, Canada	Co-Investigator	
Jason	Ding	MD	Waterloo Regional Health Network	Kitchener, Ontario, Canada	Co-Investigator	
Elyse	Wellhauser		Waterloo Regional Health Network	Kitchener, Ontario, Canada	Study Coordinator	
Stephanie	Thompson	BA	Waterloo Regional Health Network	Kitchener, Ontario, Canada	Study Coordinator	
Carol	Ballantyne		Waterloo Regional Health Network	Kitchener, Ontario, Canada	Ethics Coordinator	
Stephanie	Nemirov	BSc	Waterloo Regional Health Network	Kitchener, Ontario, Canada	Study Coordinator and Ethics Coordinator	
Rachel E.	Gutschon	MD (in progress, antici	University of Western Australia Faculty of Medicine, M	Perth, WA, Australia	Study Coordinator	
Dana L.	Martin	RPN	Woodstock Hospital	Kingston, ON, Canada	Research Nurse	
Scott	Tyldesley	MD, MPA, ABR, FRCPC	British Columbia Cancer Agency / University of British	Vancouver, Canada	Co-Investigator	
Rosalie SF.	Ho	MSc	Prostate Supportive Care Program,Vancouver Prostate C	Vancouver, Canada	Study Coordinator	Prostate Cancer Supportive Care Program
Maria Daniella	Sare	BSc	Prostate Supportive Care Program,Vancouver Prostate C	Vancouver, Canada	Study Coordinator	Prostate Cancer Supportive Care Program
Monita R.	Sundar	MA	Prostate Supportive Care Program,Vancouver Prostate C	Vancouver, Canada	Study Coordinator	Prostate Cancer Supportive Care Program
Abigail C.	O'Connor	BSc	Prostate Supportive Care Program,Vancouver Prostate C	Vancouver, Canada	Study Coordinator	Prostate Cancer Supportive Care Program
Margot	Davis	MD, SM, FRCPC	University of British Columbia - Faculty of Medicine	Vancouver, Canada	Principal Investigator	Department of Cardiology
Sean	Virani	MD, MSc, MPH, FRCI	University of British Columbia - Faculty of Medicine	Vancouver, Canada	Co-Investigator	Department of Cardiology
Brian	Clarke	MD FRCPC, FACC, F(	University of British Columbia - Faculty of Medicine	Vancouver, Canada	Co-Investigator	Department of Cardiology
Michael	Luong	MD	University of British Columbia - Faculty of Medicine	Vancouver, Canada	Co-Investigator	Department of Cardiology
Lynn	Straatman	MD	University of British Columbia - Faculty of Medicine	Vancouver, Canada	Co-Investigator	Department of Cardiology
Jaclyn	Chow	BSN	University of British Columbia/ Center for Cardiovascu	Vancouver, Canada	Research Manager	
Himantha	De Silva	BSc, BEng	University of British Columbia/ Center for Cardiovascu	Vancouver, Canada	Study Coordinator	
Audrey	Lamoureux	B. Sc	CIUSSS de l'Est de l'ile de Montreal	Montreal, Canada	Study Coordinator	
Marie-Pier	Lessard	RN	CIUSSS de l'Est de l'ile de Montreal	Montreal, Canada	Study nurse	
Diana	Kaddour	Dess	CIUSSS de l'Est de l'ile de Montreal	Montreal, Canada	Study Coordinator	
Jessica	Bou Abdallah	MSc	CIUSSS de l'Est de l'ile de Montreal	Montreal, Canada	Study Coordinator	
Nawar	Hanna	MD	CIUSSS de l'Est de l'ile de Montreal	Montreal, Canada	Principal Investigator	
Turcotte	Andrée-Anne	B.Sc	Centre intégré universitaire de santé et services sociaux de l'Estrie – Centre hospitalier universitaire de	Sherbrooke, QC, Canada	Study Coordinator	

Sabbagh	Robert	MD, M.SC, FRCSC,	Centre intégré universitaire de santé et services sociaux	Sherbrooke, QC, Canada	Principal Investigator
Richard	Patrick	MD, M.SC, FRCSC	Centre intégré universitaire de santé et services sociaux	Sherbrooke, QC, Canada	Co-Investigator
Lagabrielle	Samuel	MD, M.SC	Centre intégré universitaire de santé et services sociaux	Sherbrooke, QC, Canada	Co-Investigator
Jeldres	Claudio	MD, M.SC, FRCSC	Centre intégré universitaire de santé et services sociaux	Sherbrooke, QC, Canada	Co-Investigator
Ankita	Tiwari		Uiniveristy of Kansas Medical Center	Kansas City, Kansas USA	Study Coordinator
Amy L	Gorsuch	BA	University of Kansas Medical Center	Kansas City, Kansas USA	Study Coordinator
Elizabeth	Wulff	MD	University of Kansas Medical Center	Kansas City, Kansas USA	
Brian	Weiford	MD	University of Kansas Medical Center	Kansas City, Kansas USA	
Jennifer	Heins	PA	University of Kansas Medical Center	Kansas City, Kansas USA	
Kartik	Munshi		University of Kansas Medical Center	Kansas City, Kansas USA	
Rahul	Parikh	MD	University of Kansas Medical Center	Kansas City, Kansas USA	
Saqib	Abbasi	MD	University of Kansas Medical Center	Kansas City, Kansas USA	
Charles	Porter	MD	University of Kansas Medical Center	Kansas City, Kansas USA	Site PI (Co-Investigator}
Terry	Christsenson	RN	University of Kansas Medical Center	Kansas City, Kansas USA	Site Study Coordinator
A.E	Eisen	PhD	Rabin M.C Beilinson Campus	Prtah Tikva, Israel	Principal investigator
N.O	Oginets	B.Sc	Rabin M.C Beilinson Campus	Prtah Tikva, Israel	Study Coordinator
Daniel	Keizman	MD	Tel Aviv Sourasky medical Center	Tel Aviv	Principal investigator
Elinor	Harel	BSc	Tel Aviv Sourasky medical Center	Tel Aviv	Study coordinator
Li Ling	Tan	MBBS, MRCP (UK), M	National University Hospital Singapore	Singapore, Singapore	Principal Investigator
Chieh Yang Christopher	Koo	MBBS	National University Hospital Singapore	Singapore, Singapore	Co-Investigator
Edmund	Chiong	MBBS, FRCS	National University Hospital Singapore	Singapore, Singapore	Co-Investigator
Arshvin	Kesavan	MBBS	National University Hospital Singapore	Singapore, Singapore	Co-Investigator
Woon Chau	Tsang	MB BCh, FRCS	National University Hospital Singapore	Singapore, Singapore	Co-Investigator
Zhijiang	Zang	MB, Dip (Urol)	National University Hospital Singapore	Singapore, Singapore	Co-Investigator
Seng Cheong Alvin	Wong	MBBS, MRCP (Int Me	National University Hospital Singapore	Singapore, Singapore	Co-Investigator
Jolene	Leong	BSc	National University Hospital Singapore	Singapore, Singapore	Study Coordinator
Li Xiang Felicia	Ong	BN, MPAcc	National University Hospital Singapore	Singapore, Singapore	Study Coordinator
Shu Yi	Tan	BSc	National University Hospital Singapore	Singapore, Singapore	Study Coordinator
Lalit Mohan	Sharma	MBBS,MD	Mahatma Gandhi Hospital	Jaipur, Rajasthan, India	Principal Investigator
Ameesha	Saxena	B. Pharm, PGDM	Mahatma Gandhi Hospital	Jaipur, Rajasthan, India	Study Coordinator
Rounak	Vaishnav	BSc, MSc	Mahatma Gandhi Hospital	Jaipur, Rajasthan, India	Study Coordinator
C B	Patil	MBBS, MD (General M	Sri Shankara Cancer Hospital and Research Centre	Bangalore, Karnataka, India	Co-investigator
Ranjitha	Karanth	MBBS, Advance diplor	Sri Shankara Cancer Hospital and Research Centre	Bangalore, Karnataka, India	Co-investigator
John	Chungath	BPT,MBA(Hospital Ad	Sri Shankara Cancer Hospital and Research Centre	Bangalore, Karnataka, India	Study team (physiotherapist)
Shobha	Chandrashekaraiah	MSc(Biotechnology), P	Sri Shankara Cancer Hospital and Research Centre	Bangalore, Karnataka, India	Study Coordinator
Manila	Manjunath	Masters in Dietetics and	Sri Shankara Cancer Hospital and Research Centre	Bangalore, Karnataka, India	Study team (Dietician)
Somanagouda	Biradar	Pharm.D, PD in Clinica	Sri Shankara Cancer Hospital and Research Centre	Bangalore, Karnataka, India	Study Coordinator
Hari	Menon	MBBS,MD,DM Medic	St John's Medical college and Hospital	Bangalore, Karnataka, India	Principal Investigator
Ganesha	Vashishta	MBBS,MD,DM Medic	St John's Medical college and Hospital	Bangalore, Karnataka, India	Co-investigator
Annie	Kanchan Baa	MBBS,MD,DM Medic	St John's Medical college and Hospital	Bangalore, Karnataka, India	Co-investigator
Avinash	HU	MBBS, MD (Radiother	St John's Medical college and Hospital	Bangalore, Karnataka, India	Co-investigator
Mohan	A	MBBS MS General sur	St John's Medical college and Hospital	Bangalore, Karnataka, India	Co-investigator
Francis	Baby	MSW	St John's Medical college and Hospital	Bangalore, Karnataka, India	Study Coordinator
Akshay	L	Pharm D, RPCPO	St John's Medical college and Hospital	Bangalore, Karnataka, India	Study Coordinator
Clera	Lewis	MSW	St John's Medical college and Hospital	Bangalore, Karnataka, India	Study Coordinator
Vaddi	Surya Prakash	MBBS MS FRCS M.Cl	Kamineni academy of medical sciences and research cen	HYDERABAD , INDIA	Pincipal investigator
Palla	Shilpa	PHARM D	kamineni academy of medical sciences and research cent	HYDERABAD ,INDIA	study coordinator
Tonia	Bromley	RN	Department of Cardiovascular Medicine, Flinders Medi	Adelaide, Australia	Study Coordinator
Amanda	Weragoda	BMedSci, BMedSci Hc	Department of Cardiovascular Medicine, Flinders Medi	Adelaide, Australia	Study Coordinator
Briella K.	Egberts	BioMedSci, BMedSci I	Department of Cardiovascular Medicine, Flinders Medi	Adelaide, Australia	Study Coordinator
Sindhu	Mohanty	PhD	Crown Pincess Mary Cancer Care Centre, Westmead	Sydney, Australia	Study Coordinator
Tania	Moujaber	BMed Sci, MBBS, FRA	Crown Pincess Mary Cancer Care Centre, Westmead	Sydney, Australia	Principal Investigator
Ryan J	Appleton		Royal Adelaide Hospital	Adelaide, SA, Australia	Study Coordinator
Braden	Higgs	MBBS, FRANZCR	Royal Adelaide Hospital	Adelaide, SA, Australia	
Hien	Le	MBBS, FRANZCR	Royal Adelaide Hospital	Adelaide, SA, Australia	
Phoebe	Allison	MBBS	Royal Adelaide Hospital	Adelaide, SA, Australia	
Peter	Gorayski	BSc, MBBS,	Royal Adelaide Hospital	Adelaide, SA, Australia	
Ross	Roberts-Thomson	BMSc, MBBS	Royal Adelaide Hospital	Adelaide, SA, Australia	
Pooja	Panchal	BMS	Royal Adelaide Hospital	Adelaide, SA, Australia	
Rachael	Chang Lee	MBBS, FRACP	Royal Adelaide Hospital	Adelaide, SA, Australia	
Ashwathy	Mathew	MBBS, MD, DNB	Royal Adelaide Hospital	Adelaide, SA, Australia	
Thomas	Goode	MBBS	Royal Adelaide Hospital	Adelaide, SA, Australia	
Emma	Quinn	BClinSc, MD	Royal Adelaide Hospital	Adelaide, SA, Australia	
Boris E	Vesga	MD cardiologist	Fundación Oftalmologica de santander - FOSCAL	Floridablanca, Colombia	Co-Investigator
Valentina	Ortegón	MD	Fundación Oftalmologica de santander - FOSCAL	Floridablanca, Colombia	Sub investigator
Daniela	Castillo	MD	Fundación Oftalmologica de santander - FOSCAL	Floridablanca, Colombia	Sub investigator
Nathalia K	Amado	MD	Fundación Oftalmologica de santander - FOSCAL	Floridablanca, Colombia	Sub investigator
Laura	Celis	MD	Fundación Oftalmologica de santander - FOSCAL	Floridablanca, Colombia	Sub investigator
Sandra M	Rodriguez	Bacteriologist	Fundación Oftalmologica de santander - FOSCAL	Floridablanca, Colombia	Study Coordinator
Mayra L	Arciniegas	RN	Fundación Oftalmologica de santander - FOSCAL	Floridablanca, Colombia	Study Coordinator
Francy	Gutierrez	psychologist	Fundación Oftalmologica de santander - FOSCAL	Floridablanca, Colombia	Study Coordinator
Laura F	Neira	Bacteriologist	Fundación Oftalmologica de santander - FOSCAL	Floridablanca, Colombia	Bacteriologist
Karina B	Mejia	Bacteriologist	Fundación Oftalmologica de santander - FOSCAL	Floridablanca, Colombia	Bacteriologist
Jesus D	Polo	Pharmaceutical Chemis	Fundación Oftalmologica de santander - FOSCAL	Floridablanca, Colombia	Pharmaceutical Chemist
yudy A	Gamboa	Pharmaceutical Chemis	Fundación Oftalmologica de santander - FOSCAL	Floridablanca, Colombia	Pharmaceutical Chemist
Claudia L	Angarita	Pharmacy manager	Fundación Oftalmologica de santander - FOSCAL	Floridablanca, Colombia	Pharmacy manager
Luz J	Carvajal	Nursing assistant	Fundación Oftalmologica de santander - FOSCAL	Floridablanca, Colombia	nursing assistant
Katherine	Castrillon	Bacteriologist	Hospital Universitario San Ignacio	Bogota , Colombia	Stydy Coordinator
Viviana M	Mendez	Bacteriologist	Hospital Universitario San Ignacio	Bogota , Colombia	Study Coordinator
José E	Cita	MD, MSc	Hospital Universitario San Ignacio	Bogota , Colombia	Principal Investigator

Jorge O	Reyes	MD	Hospital Universitario San Ignacio	Bogota , Colombia	Sub Investigator
Angel A	Garcia	MD, MSc, PHD (c)	Hospital Universitario San Ignacio	Bogota , Colombia	Sub Investigator
Paola A	Pinilla	MD	Hospital Universitario San Ignacio	Bogota , Colombia	Sub Investigator
Juan E.	Gomez	MD	Fundacion Valle del Lili	Cali , Colombia	Principal Investigator
Carlos E.	Vesga	MD	Fundacion Valle del Lili	Cali , Colombia	Co-Investigator
Diana C.	Carrillo	MD	Fundacion Valle del Lili	Cali , Colombia	Co-Investigator
Brayan D.	Cordoba	MD	Fundacion Valle del Lili	Cali , Colombia	Co-Investigator
Evelyn E.	Peña	MD	Fundacion Valle del Lili	Cali , Colombia	Co-Investigator
Pablo	Sierra	MD	Fundacion Valle del Lili	Cali , Colombia	Co-Investigator
Lady T.	Delgado	MD	Fundacion Valle del Lili	Cali , Colombia	Co-Investigator
Diana M.	Rangel	PT	Fundacion Valle del Lili	Cali , Colombia	Study Coordinator
Sara N.	Burbano	MD	Fundacion Valle del Lili	Cali , Colombia	Co-Investigator
Gloria I	Jimenez	PT	Fundacion Valle del Lili	Cali , Colombia	Study Coordinator
Laura A	Trujillo	SLP	Fundacion Valle del Lili	Cali , Colombia	Study Coordinator
Harrison A	Vargas	RN	Fundacion Valle del Lili	Cali , Colombia	Study Coordinator
Ana L	Cuaran	PT	Fundacion Valle del Lili	Cali , Colombia	Study Coordinator
Melo	Edielle		Instituto do Coração do Hospital das Clínicas da Faculda	São Paulo, SP, Brazil	Co-Investigator
Moulin	Silvia		Instituto do Coração do Hospital das Clínicas da Faculda	São Paulo, SP, Brazil	Co-Investigator
Nascimento da Silva	Beatriz		Instituto do Coração do Hospital das Clínicas da Faculda	São Paulo, SP, Brazil	Study Coordinator
Fellini	Mariella		Instituto do Coração do Hospital das Clínicas da Faculda	São Paulo, SP, Brazil	Study Coordinator
Carneiro	Sabrina		Instituto do Coração do Hospital das Clínicas da Faculda	São Paulo, SP, Brazil	Study Coordinator
Rafael A. B.	Nunes	MD, PhD	Hospital Alemão Oswaldo Cruz	São Paulo, SP, Brazil	Co-Investigator
Precil D. M. M.	Neves	MD, PhD	Hospital Alemão Oswaldo Cruz	São Paulo, SP, Brazil	Co-Investigator
Carlo C.	Passerotti	MD	Hospital Alemão Oswaldo Cruz	São Paulo, SP, Brazil	Co-Investigator
Emili G. M.	Ayoub	MD	Hospital Alemão Oswaldo Cruz	São Paulo, SP, Brazil	Co-Investigator
Eliza D.	Ricardo	MD	Hospital Alemão Oswaldo Cruz	São Paulo, SP, Brazil	Co-Investigator
José	Pontes	MD	Hospital Alemão Oswaldo Cruz	São Paulo, SP, Brazil	Co-Investigator
Antonia L. L.	Rodrigues	BSc, MSc	Hospital Alemão Oswaldo Cruz	São Paulo, SP, Brazil	Study Coordinator
Juliete J.	Vidotti	Bsc	Hospital Alemão Oswaldo Cruz	São Paulo, SP, Brazil	Research Assistant
Beatriz M.	Sabino	Bsc	Hospital Alemão Oswaldo Cruz	São Paulo, SP, Brazil	Regulatory
Severina M. F.	Da Silva	Bsc	Hospital Alemão Oswaldo Cruz	São Paulo, SP, Brazil	Research Assistant
Luana O.	Dos Santos	Bsc	Hospital Alemão Oswaldo Cruz	São Paulo, SP, Brazil	Study Coordinator
Roberto	Odebrecht R.	MD	Hospital Santa Marcelina	São Paulo, SP, Brazil	Principal Investigator
Tércia	Soares S.	MD	Hospital Santa Marcelina	São Paulo, SP, Brazil	Co-investigator
Camila	Camargo	BSc	Hospital Santa Marcelina	São Paulo, SP, Brazil	Study Coordinator
Denise	Sousa	BSc	Hospital Santa Marcelina	São Paulo, SP, Brazil	Study Coordinator
Agatha	Rogério D.	BSc	Hospital Santa Marcelina	São Paulo, SP, Brazil	Study Coordinator
Maria	Grapeia A.	Technician	Hospital Santa Marcelina	São Paulo, SP, Brazil	Nursing technician
Juliana	Paula N.	BSc	Hospital Santa Marcelina	São Paulo, SP, Brazil	Study Coordinator
Antônio José	Mata F.	MD	Hospital Santa Marcelina	São Paulo, SP, Brazil	Co-investigator
Camila G.	Ferrari	MD	Faculdade de Medicina de São José do Rio Preto FAME	São José do Rio Preto, SP, Brazil	Principal Investigator
Fabio L. Couto	Fernandez	M	Faculdade de Medicina de São José do Rio Preto FAME	São José do Rio Preto, SP, Brazil	Co-Investigator
Luiza A. B. Leite	Ferreira	MD	Faculdade de Medicina de São José do Rio Preto FAME	São José do Rio Preto, SP, Brazil	Co-Investigator
João A.	Soler	MD	Faculdade de Medicina de São José do Rio Preto FAME	São José do Rio Preto, SP, Brazil	Co-Investigator
Bruno C. M. Uchoa	Junior	MD	Faculdade de Medicina de São José do Rio Preto FAME	São José do Rio Preto, SP, Brazil	Co-Investigator
João Daniel C.	Guedes	MD	Faculdade de Medicina de São José do Rio Preto FAME	São José do Rio Preto, SP, Brazil	Co-Investigator
Kathia C.	Abdalla	MD	Faculdade de Medicina de São José do Rio Preto FAME	São José do Rio Preto, SP, Brazil	Co-Investigator
Ana Paula D.	Martins	BSN	Faculdade de Medicina de São José do Rio Preto FAME	São José do Rio Preto, SP, Brazil	Site coordinator
Katia	Nishyama	BSN	Faculdade de Medicina de São José do Rio Preto FAME	São José do Rio Preto, SP, Brazil	Site coordinator
Osvaldo L. da Silva	Junior	MA	Faculdade de Medicina de São José do Rio Preto FAME	São José do Rio Preto, SP, Brazil	Site coordinator
Claudio Luis	Tedokon		Faculdade de Medicina de São José do Rio Preto FAME	São José do Rio Preto, SP, Brazil	Research assistant
Denise Alexandra	Santos		Faculdade de Medicina de São José do Rio Preto FAME	São José do Rio Preto, SP, Brazil	Research assistant
Isabelly C. M.	Neves		Faculdade de Medicina de São José do Rio Preto FAME	São José do Rio Preto, SP, Brazil	Research assistant
Fabiola	Martos		Faculdade de Medicina de São José do Rio Preto FAME	São José do Rio Preto, SP, Brazil	Research assistant
Fernanda Lima	Paglione	BSN	Faculdade de Medicina de São José do Rio Preto FAME	São José do Rio Preto, SP, Brazil	Study coordinator
Camila Costa	Silva	BSN	Faculdade de Medicina de São José do Rio Preto FAME	São José do Rio Preto, SP, Brazil	Study coordinator
Gabriela Taparo	Castro	BSN	Faculdade de Medicina de São José do Rio Preto FAME	São José do Rio Preto, SP, Brazil	Study coordinator
Bruna C.	Marques	BSC	Faculdade de Medicina de São José do Rio Preto FAME	São José do Rio Preto, SP, Brazil	Study coordinator
Beatriz P.	Scarpim	BSN	Faculdade de Medicina de São José do Rio Preto FAME	São José do Rio Preto, SP, Brazil	Study coordinator
Luciana R. Cubas	Volpe	BSN	Faculdade de Medicina de São José do Rio Preto FAME	São José do Rio Preto, SP, Brazil	Study coordinator
Thamyres S. A.	Cruz	BSN	Faculdade de Medicina de São José do Rio Preto FAME	São José do Rio Preto, SP, Brazil	Study coordinator
Thiago B.	Souza	MD	Faculdade de Medicina de São José do Rio Preto FAME	São José do Rio Preto, SP, Brazil	Regulatory
Juliane M.	Matos	BSC	Faculdade de Medicina de São José do Rio Preto FAME	São José do Rio Preto, SP, Brazil	Regulatory
Jaqueline R.	Mastro		Faculdade de Medicina de São José do Rio Preto FAME	São José do Rio Preto, SP, Brazil	Regulatory
Thamires W. L.	Santana	BSN	Faculdade de Medicina de São José do Rio Preto FAME	São José do Rio Preto, SP, Brazil	Regulatory
Gabriela P.	Bessa		Faculdade de Medicina de São José do Rio Preto FAME	São José do Rio Preto, SP, Brazil	Technical nurse
Maria Fernanda	Santos		Faculdade de Medicina de São José do Rio Preto FAME	São José do Rio Preto, SP, Brazil	Technical nurse
Elaine B.	Gallo		Faculdade de Medicina de São José do Rio Preto FAME	São José do Rio Preto, SP, Brazil	Technical nurse
Lilian M. S.	Pinho	MS	Faculdade de Medicina de São José do Rio Preto FAME	São José do Rio Preto, SP, Brazil	Nurse
Joelma I.	Silva		Faculdade de Medicina de São José do Rio Preto FAME	São José do Rio Preto, SP, Brazil	Technical nurse
João Pedro H.	Netto	MD	Faculdade de Medicina de São José do Rio Preto FAME	São José do Rio Preto, SP, Brazil	Co-Investigator
Mayra C. J.	Frankenfeld	MD	Unidade de Pesquisa Clínica da Faculdade de Medicina	Botucatu, SP, Brazil	Principal Investigator
Ana L.	Coradazzi	PhD	Unidade de Pesquisa Clínica da Faculdade de Medicina	Botucatu, SP, Brazil	Co-investigator
Claudia V.	Cassaro	MSc	Unidade de Pesquisa Clínica da Faculdade de Medicina	Botucatu, SP, Brazil	Study Coordinator
Marina N	Crispim	BSc	Hospital de Clínicas de Porto Alegre	Porto Alegre, RS, Brazil	Study Coordinator
Henrique I.	Garbin	BSc, MSc, Phd, MD	Hospital de Clínicas de Porto Alegre	Porto Alegre, RS, Brazil	Co-investigator
Andrei D. P.	Rickrot	Clinical Laboratory Tec	Hospital de Clínicas de Porto Alegre	Porto Alegre, RS, Brazil	Research Assistant
André B.	Amon	BSc, MD	Hospital de Clínicas de Porto Alegre	Porto Alegre, RS, Brazil	Co-investigator
Milene F.	Sehn	BSc, MD	Hospital de Clínicas de Porto Alegre	Porto Alegre, RS, Brazil	Co-investigator
Raquel O.	Luiz	BSc, RN	Hospital de Clínicas de Porto Alegre	Porto Alegre, RS, Brazil	Study Coordinator

Lais P.	Lopes	MSc, BSc	Hospital de Clínicas de Porto Alegre	Porto Alegre, RS, Brazil	Research Assisstant
Vera L. O.	Freitas	MSc, PhD	Hospital de Clínicas de Porto Alegre	Porto Alegre, RS, Brazil	Study Coordinator
Fernanda R.	Cunegatto	MSc, BSc	Hospital de Clínicas de Porto Alegre	Porto Alegre, RS, Brazil	Study Coordinator
Taina dos. S	Soares	BSc (Biomedical), MSc	Hospital de Clínicas de Porto Alegre	Porto Alegre, RS - Brasil	Study Coordinator
Rafaela S.	Da Silva	BSc (Nutritionist)	Hospital de Clínicas de Porto Alegre	Porto Alegre, RS - Brasil	Research Assistant
Estefania V. A.	Andrade	BSc (MD), Cardiologist	Hospital de Clínicas de Porto Alegre	Porto Alegre, RS - Brasil	Co-investigator
Lívia F.A.	Oliveira	MD. MSC	Hospital de Clínicas da Universidade Federal do Triangulo Mineiro	Uberaba, MG, Brasil	Principal Investigator
Fabio R.	Oliveira	MD	Hospital de Clínicas da Universidade Federal do Triangulo Mineiro	Uberaba, MG, Brasil	Co- Investigator
Karen B.	Ribeiro	MD, PHD	Hospital de Clínicas da Universidade Federal do Triangulo Mineiro	Uberaba, MG, Brasil	Co-Investigator
Giovani L.	Santi	MD, PHD	Hospital de Clínicas da Universidade Federal do Triangulo Mineiro	Uberaba, MG, Brasil	Co- investigator
Lucinda C.	Guimarães	MD, PHD	Hospital de Clínicas da Universidade Federal do Triangulo Mineiro	Uberaba, MG, Brasil	Co- Investigator
Paulo H.M.	Vilela	MD	Hospital de Clínicas da Universidade Federal do Triangulo Mineiro	Uberaba, MG, Brasil	Co-Investigator
Paula M.	Camasmie	PHD (in progress)	Hospital de Clínicas da Universidade Federal do Triangulo Mineiro	Uberaba, MG, Brasil	Study Coordinator
Raíssa B.	Luiz	PHD	Hospital de Clínicas da Universidade Federal do Triangulo Mineiro	Uberaba, MG, Brasil	Study Coordinator
Aline M.	Carlos	PHD	Hospital de Clínicas da Universidade Federal do Triangulo Mineiro	Uberaba, MG, Brasil	Study Coordinator
Katsuki A.	Tiscoski	MD	Santa Casa de Misericordia de Porto Alegre	Porto Alegre, RS, Brazil	Principal Investigator
Bruna B.	Motke	MD	Santa Casa de Misericordia de Porto Alegre	Porto Alegre, RS, Brazil	Co-investigator
Anita M.	Maciel	BSc	Santa Casa de Misericordia de Porto Alegre	Porto Alegre, RS, Brazil	Data Manager
Antonella	Kaul	BSc	Santa Casa de Misericordia de Porto Alegre	Porto Alegre, RS, Brazil	Study Coordinator
Gustavo M.	Escott	PhD	Santa Casa de Misericordia de Porto Alegre	Porto Alegre, RS, Brazil	Study Coordinator
Celina F	Silva	BSc	Santa Casa de Misericordia de Porto Alegre	Porto Alegre, RS, Brazil	Research assistant
Leticia M. S.	Souza	BSc	Santa Casa de Misericordia de Porto Alegre	Porto Alegre, RS, Brazil	Study Coordinator
Giovanna M.	Barros	Underdegree Pharmacis	Santa Casa de Misericordia de Porto Alegre	Porto Alegre, RS, Brazil	Pharmacist
Guilherme L.	Santos	Underdegree BSc	Santa Casa de Misericordia de Porto Alegre	Porto Alegre, RS, Brazil	Regulatory/Financial Analyst
Lorenzo S.	Barcelos	BSc	Santa Casa de Misericordia de Porto Alegre	Porto Alegre, RS, Brazil	Data Manager
Carolina P.	Pereira	BSc	Santa Casa de Misericordia de Porto Alegre	Porto Alegre, RS, Brazil	Pharmacist
Patrícia E. C.	Silva	BSc	Santa Casa de Misericordia de Porto Alegre	Porto Alegre, RS, Brazil	Study Coordinator
Leonardo M.	Santos	BSc	Santa Casa de Misericordia de Porto Alegre	Porto Alegre, RS, Brazil	Study Coordinator
Tatiane M. R.	Reis	BSc	Santa Casa de Misericordia de Porto Alegre	Porto Alegre, RS, Brazil	Study Coordinator
Lavinia P.	Carvalho	Msc	Santa Casa de Misericordia de Porto Alegre	Porto Alegre, RS, Brazil	Study Coordinator
Lauren M.	Xavier	BSc	Santa Casa de Misericordia de Porto Alegre	Porto Alegre, RS, Brazil	Study Coordinator
Camila G.	Soares	BSc	Santa Casa de Misericordia de Porto Alegre	Porto Alegre, RS, Brazil	Regulatory Assistant
Júlia L.	Deimling	BSc	Santa Casa de Misericordia de Porto Alegre	Porto Alegre, RS, Brazil	Study Coordinator
Alessandra O.S	Santos	BSc	Irmandade da Santa casa de Misericordia de São Paulo	São Paulo, Brasil	Study Coordinator
Daniela S.	Santana	BSc	Irmandade da Santa casa de Misericordia de São Paulo	São Paulo, Brasil	Study Coordinator
Luis E.S.M	Moz	MD, PhD	Irmandade da Santa casa de Misericordia de São Paulo	São Paulo, Brasil	Principal investigator
Lin I	Ter	PhD	Irmandade da Santa casa de Misericordia de São Paulo	São Paulo/SP, Brazil	
Luana Carolina F F	Gelschyn	MSc	Irmandade da Santa casa de Misericordia de São Paulo	São Paulo/SP, Brazil	
Roni C	Fernandes	PhD	Irmandade da Santa casa de Misericordia de São Paulo	São Paulo/SP, Brazil	
Paula	Lemos	BSc	Irmandade da Santa casa de Misericordia de São Paulo		
Laise M M	de Sousa	BSc	Irmandade da Santa casa de Misericordia de São Paulo	São Paulo/SP, Brazil	
Luis Eduardo S	Móz	PhD	Irmandade da Santa casa de Misericordia de São Paulo	São Paulo/SP, Brazil	
Eduardo	Schlabendorff	MD	IMV Pesquisa Cardiológica	Porto Alegre, RS, Brazil	Principal Investigator
Vanessa Santos	dos Santos	MD	IMV Pesquisa Cardiológica	Porto Alegre, RS, Brazil	Co-Investigator
Ana Luiza	Abrahao	MD	IMV Pesquisa Cardiológica	Porto Alegre, RS, Brazil	Co-Investigator
Cristine Erdmann	Nunes	MD	IMV Pesquisa Cardiológica	Porto Alegre, RS, Brazil	Co-Investigator
Carollainy Nascimento	Silva	B.Sc.	IMV Pesquisa Cardiológica	Porto Alegre, RS, Brazil	Study Nurse/Coordinator
Aline Gonçalves	Borba	BBA	IMV Pesquisa Cardiológica	Porto Alegre, RS, Brazil	Regulatory Assistant
Amanda Fernandes	Barbosa	B.Sc.	IMV Pesquisa Cardiológica	Porto Alegre, RS, Brazil	Study Nurse/Coordinator
Gabriela Petry	Crestani	B.Sc.	IMV Pesquisa Cardiológica	Porto Alegre, RS, Brazil	Study Nurse/Coordinator
Cristiano Pederneiras	Jaeger	MSc	IMV Pesquisa Cardiológica	Porto Alegre, RS, Brazil	Co-Investigator
Karen Brasil	Ruschel	PhD	IMV Pesquisa Cardiológica	Porto Alegre, RS, Brazil	Site Manager
Murillo O	Antunes	MD, PHD	Hospital Universitário São Francisco na Providência de Deus	Bragança Paulista, SP, Brazil	Principal Investigator
Mariana R	Gonçalves	RN	Hospital Universitário São Francisco na Providência de Deus	Bragança Paulista, SP, Brazil	Stydy Coordinator
Simone	Felitti	MD	Hospital Universitário São Francisco na Providência de Deus	Bragança Paulista, SP, Brazil	Sub Investigator
Julia V	Matos	BSc	Hospital Universitário São Francisco na Providência de Deus	Bragança Paulista, SP, Brazil	Study Coordinator
Karollyny A	Salgueiro	BSc	Hospital Universitário São Francisco na Providência de Deus	Bragança Paulista, SP, Brazil	Study Coordinator
Joice C	Couto	BSc	Hospital Universitário São Francisco na Providência de Deus	Bragança Paulista, SP, Brazil	Pharmacist
Laura D	Cassab	BSc	Hospital Universitário São Francisco na Providência de Deus	Bragança Paulista, SP, Brazil	Study Coordinator
Bianca A. B.	Kuhn	B.Sc	Associação Hopital de Caridade de Ijuí - Núcleo de Ensino e Pesquisa	Ijuí, RS, Brazil	Study Coordinator
Gabriela	Strucker	B.Sc	Associação Hopital de Caridade de Ijuí - Núcleo de Ensino e Pesquisa	Ijuí, RS, Brazil	Study Coordinator
Gabrielly M.	Ribeiro	B.Sc	Associação Hopital de Caridade de Ijuí - Núcleo de Ensino e Pesquisa	Ijuí, RS, Brazil	Study Coordinator
Juliana S.	Pereira	B.Sc	Associação Hopital de Caridade de Ijuí - Núcleo de Ensino e Pesquisa	Ijuí, RS, Brazil	Study Coordinator
Fernanda B.	Uggeri	M.Sc	Associação Hopital de Caridade de Ijuí - Núcleo de Ensino e Pesquisa	Ijuí, RS, Brazil	Study Coordinator
Amanda	Panisson	B.Sc	Associação Hopital de Caridade de Ijuí - Núcleo de Ensino e Pesquisa	Ijuí, RS, Brazil	Study Coordinator
Francini O.	Rodrigues	M.Sc	Associação Hopital de Caridade de Ijuí - Núcleo de Ensino e Pesquisa	Ijuí, RS, Brazil	Study Coordinator
Leticia Y	Castro	MD, M.Sc	Associação Hopital de Caridade de Ijuí - Núcleo de Ensino e Pesquisa	Ijuí, RS, Brazil	Co-Investigator
Edilson	Walter	MD, B.Sc	Associação Hopital de Caridade de Ijuí - Núcleo de Ensino e Pesquisa	Ijuí, RS, Brazil	Co-Investigator
Cristiano F	Souza	MD	Instituto Brasileiro de Controle do Cancer - IBCC	São Paulo/SP, Brazil	Principal Investigator
Gabriel R	Figueiredo	MD	Instituto Brasileiro de Controle do Cancer - IBCC	São Paulo/SP, Brazil	Co-investigator
Maria E. M.	Siqueira	MD	Instituto Brasileiro de Controle do Cancer - IBCC	São Paulo/SP, Brazil	Co-investigator
Adriana N. M.	Basílio	PHD	Instituto Brasileiro de Controle do Cancer - IBCC	São Paulo/SP, Brazil	Study Coordinator (secondary)
Giovanna	Uehara	BSc	Instituto Brasileiro de Controle do Cancer - IBCC	São Paulo/SP, Brazil	Study Coordinator (primary)
Michelle P	Silva	BSc	Instituto Brasileiro de Controle do Cancer - IBCC	São Paulo/SP, Brazil	Study Nurse
Marilia F	Nascimento	BSc	Instituto Brasileiro de Controle do Cancer - IBCC	São Paulo/SP, Brazil	Study Nurse
Rosangela S	Costa	BSc	Instituto Brasileiro de Controle do Cancer - IBCC	São Paulo/SP, Brazil	Study Nurse
Lenilda A M	Santos	BSc	Instituto Brasileiro de Controle do Cancer - IBCC	São Paulo/SP, Brazil	Study Nurse
Marina	Leonardo	BSc	Instituto Brasileiro de Controle do Cancer - IBCC	São Paulo/SP, Brazil	Study Coordinator
Giulia O	Toffolo	BSc	Instituto Brasileiro de Controle do Cancer - IBCC	São Paulo/SP, Brazil	Study Coordinator
Alayne M. T. D.	Yamada	PHD	Instituto Brasileiro de Controle do Cancer - IBCC	São Paulo/SP, Brazil	Study Coordinator

Graziele O.	Santos		BSc	Instituto Brasileiro de Controle do Cancer - IBCC	São Paulo/SP, Brazil	Study Nurse	
Patricia M. M.	Beato		BSc (MD), Oncologist	Fundação Doutor Amaral Carvalho	Jaú, SP - Brazil	Principal investigator	
Luana D.	Vieira		BSc	Fundação Doutor Amaral Carvalho	Jaú, SP - Brazil	Study coordinator	
Gustavo S.	Santos		BSc, MSc	Fundação Doutor Amaral Carvalho	Jaú, SP - Brazil	Study coordinator	
Felipe A. C.	Carboni		BSc (MD), Cardiologist	Fundação Doutor Amaral Carvalho	Jaú, SP - Brazil	Co-investigator	
Thales G.	May		BSc (MD), Cardiologist	Fundação Doutor Amaral Carvalho	Jaú, SP - Brazil	Co-investigator	
Brigida	Ranzani		BSc (MD), Oncologist	Fundação Doutor Amaral Carvalho	Jaú, SP - Brazil	Co-investigator	
Hellen C. G.	Costa		BSc (MD), Oncologist	Fundação Doutor Amaral Carvalho	Jaú, SP - Brazil	Co-investigator	
Mariana G.	Cardoso		BSc (MD), Oncologist	Fundação Doutor Amaral Carvalho	Jaú, SP - Brazil	Co-investigator	
Natalia L.	Rodrigues		BSc (MD), Oncologist	Fundação Doutor Amaral Carvalho	Jaú, SP - Brazil	Co-investigator	
Thais P.	Ribeiro		RN	Hospital Felicio Rocho	Belo Horizonte, Minas Gerais, Brazil	Study Coordinator	
Luanna L. F.	Gonçalves			Hospital Felicio Rocho	Belo Horizonte, Minas Gerais, Brazil	Study Coordinator	
Livia A.	Pereira		MD Cardiologist	Hospital Felicio Rocho	Belo Horizonte, Minas Gerais, Brazil	Principal investigator	
Renata P.	Costa		MD Oncologist	Hospital Felicio Rocho	Belo Horizonte, Minas Gerais, Brazil	Co-investigator	
Maria J. A.	Tostes		RN	Hospital Felicio Rocho	Belo Horizonte, Minas Gerais, Brazil	Study Coordinator	
Carlos A. K.	Nakashima		MD, PhD	Sociedade Hospitalar Angelina Caron	Campina Grande do Sul, PR, Brazil	Principal Investigator	
Dalton B.	Precoma		MD, PhD	Sociedade Hospitalar Angelina Caron	Campina Grande do Sul, PR, Brazil	Co-investigator	
Lilian	Belinaso		MD	Sociedade Hospitalar Angelina Caron	Campina Grande do Sul, PR, Brazil	Co-investigator	
Camila	Richter		MD	Sociedade Hospitalar Angelina Caron	Campina Grande do Sul, PR, Brazil	Co-investigator	
Cristina P.	Correa		RN	Sociedade Hospitalar Angelina Caron	Campina Grande do Sul, PR, Brazil	Study Coordinator	
Eliane	Sehnem		RN	Sociedade Hospitalar Angelina Caron	Campina Grande do Sul, PR, Brazil	Study Coordinator	
Ariella P. B.	Bongiorno		BSc	Sociedade Hospitalar Angelina Caron	Campina Grande do Sul, PR, Brazil	Study Coordinator	
Méllanie A. S.	Ferreira		MSc, BSc	Sociedade Hospitalar Angelina Caron	Campina Grande do Sul, PR, Brazil	Study Coordinator	
Ketlyn C. P.	Silva		BSc	Sociedade Hospitalar Angelina Caron	Campina Grande do Sul, PR, Brazil	Research assistant	
Gustavo L	Marques			Universidade Federal do Paraná	Curitiba, Brazil	Principal Investigator	
Rafael M	Miyazima		MD	Universidade Federal do Paraná	Curitiba, Brazil		
Eduarda Larissa Soares	Silva		PgDip, NURSE	Instituto de Medicina Integral Professor Fernando Figue	Recife, Pernambuco, Brazil	Study Coordinator	
Nathalia Santos Barbosa da	Silva		PgDip, NURSE	Instituto de Medicina Integral Professor Fernando Figue	Recife, Pernambuco, Brazil	Study Coordinator	
Laíse Maria Xavier da	Silva		Graduate	Instituto de Medicina Integral Professor Fernando Figue	Recife, Pernambuco, Brazil	regulatory affairs analyst	
Jurema Telles de Oliveira	Lima		PhD	Instituto de Medicina Integral Professor Fernando Figue	Recife, Pernambuco, Brazil	Principal investigator	
Maria Isabelly de Melo	Canejo		PgDip, NURSE	Instituto de Medicina Integral Professor Fernando Figue	Recife, Pernambuco, Brazil	Study Coordinator	
Andrea Lopes Ponte de	Souza		PhD	Instituto de Medicina Integral Professor Fernando Figue	Recife, Pernambuco, Brazil	Co-investigator	
Danielle Menor	Vasconcelos		PhD	Instituto de Medicina Integral Professor Fernando Figue	Recife, Pernambuco, Brazil	Study Coordinator	
Henrique de Sá Carneiro	Mota		PgDip, cardiologist	Instituto de Medicina Integral Professor Fernando Figue	Recife, Pernambuco, Brazil	Co-investigator	
Mateus Couto Tavares	Feitosa		PgDip	Instituto de Medicina Integral Professor Fernando Figue	Recife, Pernambuco, Brazil	Co-investigator	
Júlia Marthy Soares Santos	Santiago		PgDip	Instituto de Medicina Integral Professor Fernando Figue	Recife, Pernambuco, Brazil	Clinical Research Analyst	
Carla Rameri Alexandre Silva	Azevedo		PhD	Instituto de Medicina Integral Professor Fernando Figue	Recife, Pernambuco, Brazil	Co-investigator	
João	Borges		MD	Associação de Amparo à Saúde, Ensino e Pesquisa	Lago Sul, Brasília, Brazil	Principal Investigator	
Beatriz	Montenegro		MD	Associação de Amparo à Saúde, Ensino e Pesquisa	Lago Sul, Brasília, Brazil	Sub Investigator	
Greyce	Campos		RN	Associação de Amparo à Saúde, Ensino e Pesquisa	Lago Sul, Brasília, Brazil	Study Coordinator	
Sarah	Fernandes		RN	Associação de Amparo à Saúde, Ensino e Pesquisa	Lago Sul, Brasília, Brazil	Study Coordinator	
TC	GUIMARAES		PHD NURSE IN CARI	INSTITUTO NACIONA DE CARDIOLOGIA	RIO DE JANEIRO - RJ - BRASIL	STUDY NURSE/ STUDY COORDINATOR	
AF	ISSA		PhD and Master's Degre	INSTITUTO NACIONAL DE CARDIOLOGIA	RIO DE JANEIRO - RJ - BRASIL	PRINCIPAL INVESTIGATOR	Director of the National Institute of Cardiology - SAES/MS/RJ
MR	FARIA		MEDICINE DEGREE	INSTITUTO NACIONAL DE CARDIOLOGIA	RIO DE JANEIRO - RJ - BRASIL	SUBINVESTIGATOR	
FL	ARAGÃO		RN	INSTITUTO NACIONAL DE CARDIOLOGIA	RIO DE JANEIRO - RJ - BRASIL	STUDY NURSE	
LC	FIALHO		BUSINESS AND ADM	INSTITUTO NACIONAL DE CARDIOLOGIA	RIO DE JANEIRO - RJ - BRASIL	STUDY COORDINATOR	
PA	LEITE		PHYSICIAN STUDEN	INSTITUTO NACIONAL DE CARDIOLOGIA	RIO DE JANEIRO - RJ - BRASIL		
TA	MARINHO		Master's Degree in Me	INSTITUTO NACIONAL DO CANCER	RIO DE JANEIRO - RJ - BRASIL	SUBINVESTIGATOR	
Fernando	Alfieri	Jr	MD GI Specialist	Beneficência Nipo-Brasileira de São Paulo - Hospital Ni	São Paulo/SP, Brazil	Beneficência Nipo-Brasileira de São Paulo - Hospital Nipo-Brasileiro Medical Superint.	
Maristela	Miranda		MD Oncologist	Beneficência Nipo-Brasileira de São Paulo - Hospital Ni	São Paulo/SP, Brazil	Principal Investigator (replaced by Marcio Kawano, MD)	
Manfredo K	Naritomi		MD Cardiologist	Beneficência Nipo-Brasileira de São Paulo - Hospital Ni	São Paulo/SP, Brazil	Sub-Investigator	
Walquiria C R	Ritter		BSc	Beneficência Nipo-Brasileira de São Paulo - Hospital Ni	São Paulo/SP, Brazil	Study Coordinator (replaced by Rosana C Pagliarini)	
Marcio	Kawano		MD Oncologist	Beneficência Nipo-Brasileira de São Paulo - Hospital Ni	São Paulo/SP, Brazil	Principal Investigator	
Rosana C	Pagliarini		BSc	Beneficência Nipo-Brasileira de São Paulo - Hospital Ni	São Paulo/SP, Brazil	Study Coordinator	
Ayrton C G	Santos		BSc grad	Beneficência Nipo-Brasileira de São Paulo - Hospital Ni	São Paulo/SP, Brazil	Clinical Trials Assistant	
Cesar	Dusilek		MD	Hospital do Rocio	Campo Largo, PR, Brazil	Principal Investigator	
Diego	Maia		MD	Hospital do Rocio	Campo Largo, PR, Brazil	Co-Investigator	
Gabrielly	Lech		BCs	Hospital do Rocio	Campo Largo, PR, Brazil	Study Coordinator	
Bianca	Bocalon		Msc	Hospital Municipal de Barueri	Barueri, SP - Brazil	Study coordinator	
Ana Paula	Araujo		Bsc	Hospital Municipal de Barueri	Barueri, SP - Brazil	Study coordinator	
Paulo	Tierno		Bsc	Hospital Municipal de Barueri	Barueri, SP - Brazil	Principal Investigator	
Felipe	Grabarz		BSc, MSc, PhD	InCor - HCFMUSP	São Paulo, SP - Brazil	National Leader Office - CRA	
Adriadne Justi	Bertolin		MD, PhD	InCor - HCFMUSP	São Paulo, SP - Brazil	National Leader Office - Medical Coordinator	
Marcelly Soares	Rosal		BSc	InCor - HCFMUSP	São Paulo, SP - Brazil	National Leader Office - Regulatory Affairs Monitor	
Alexandra	Vieira		Pharm, MSc	InCor - HCFMUSP	São Paulo, SP - Brazil	National Leader Office - CRA	
Felipe	Martins		BSc, MSc	InCor - HCFMUSP	São Paulo, SP - Brazil	National Leader Office - CRA	
Marina	Dannias		Pharm	InCor - HCFMUSP	São Paulo, SP - Brazil	National Leader Office - Regulatory Affairs Monitor	
José	Leon		MD	InCor - HCFMUSP	São Paulo, SP - Brazil	National Leader Office - CRA	
Amanda	Pedroso		BSc, PhD	InCor - HCFMUSP	São Paulo, SP - Brazil	National Leader Office - Regulatory Affairs Monitor	
Marisol	Lopez-Pico		Msc	Masira Research Institute, Universidad de Santander (U	Bucaramanga, Colombia	National Leader Office Staff	
Deepa	Srinivasan		MD	St. John's Research Institute	Bangalore, Karnataka, India	National Leader Office - Co-Investigator	
Aakanksha	Singh		BDS, MPH	St. John's Research Institute	Bangalore, Karnataka, India	National Leader Office - Study Coordinator	
Freeda	Xavier		MSc	St. John's Research Institute	Bangalore, Karnataka, India	National Leader Office - Study Coordinator	
Preeti	Girish		MSc	St. John's Research Institute	Bangalore, Karnataka, India	National Leader Office - Study Coordinator	
Anthea M	Rayan		MSc	St. John's Research Institute	Bangalore, Karnataka, India	National Leader Office - Study Coordinator	
Pablo	Lamellas		MD, MSc	Fundación Favaloro	Buenos Aires, Argentina	Adjudication Committee	
David	Margel		MD, PhD	Assuta Medical Center/Rabin Medical Center	Tel Aviv	Adjudication Committee	
Francois	Caron		MD	Population Health Research Institute	Hamilton, Ontario, Canada	Adjudication Committee	
Malik	Farooqi		MD	Population Health Research Institute	Hamilton, Ontario, Canada	Adjudication Committee	
Som	Mukherjee		MD	Hamilton Health Sciences / McMaster University	Hamilton, Canada	Adjudication Committee	

Germano D.	Cavalli	MD	Population Health Research Institute	Hamilton, Ontario, Canada	Adjudication Committee, Cardio-Oncology Fellowship
Rocio C.	Baro Vila	MD	Population Health Research Institute	Hamilton, Ontario, Canada	Adjudication Committee, Cardio-Oncology Fellowship
Alexander	Benz	MD	Population Health Research Institute	Hamilton, Ontario, Canada	Adjudication Committee
Rachel	Richard	MD	Université de Sherbrooke	Sherbrooke, QC, Canada	Adjudication Committee, Cardio-Oncology Fellowship
George	Wells	MSc, PhD, MD	University of Ottawa Heart Institute / Ottawa Hospital Research Institute	Ottawa, Ontario, Canada	Data Safety Monitoring Board (DSMB)
Jean	Rouleau	MD	Université de Montréal	Montreal, Quebec, Canada	Data Safety Monitoring Board (DSMB)
Michael L.	Cher	MD	Karmanos Cancer Center	Detroit, Michigan, USA	Data Safety Monitoring Board (DSMB)
Cissy	Tang	MSc	Population Health Research Institute	Hamilton, Ontario, Canada	Study Statisticians and Programmers
Mark	Molec		Population Health Research Institute	Hamilton, Ontario, Canada	Study Statisticians and Programmers
Nishitha	Jeyachandradhas	M.E	Population Health Research Institute	Hamilton, Ontario, Canada	Study Statisticians and Programmers
Matthew	Smart		Population Health Research Institute	Hamilton, Ontario, Canada	ICT
Les	Farago		Population Health Research Institute	Hamilton, Ontario, Canada	ICT
Gianluca	Rigitano		Population Health Research Institute	Hamilton, Ontario, Canada	ICT
Miroslaw	Malysz		Population Health Research Institute	Hamilton, Ontario, Canada	ICT
Shrouk	Saed		Population Health Research Institute	Hamilton, Ontario, Canada	ICT
Nidhi	Jethoo		Population Health Research Institute	Hamilton, Ontario, Canada	ICT
Peter	Accardo	BSc, MSc	Population Health Research Institute	Hamilton, Ontario, Canada	Project Office Study Staff
Amanda	DeJesus	BA	Population Health Research Institute	Hamilton, Ontario, Canada	Project Office Study Staff
Maxwell	Crispo		Population Health Research Institute	Hamilton, Ontario, Canada	Project Office Study Staff
Rhonda	Masney		Population Health Research Institute	Hamilton, Ontario, Canada	Project Office Study Staff
Sabrina	Woolgar		Population Health Research Institute	Hamilton, Ontario, Canada	Project Office Study Staff
Ruchi	Patel		Population Health Research Institute	Hamilton, Ontario, Canada	Project Office Study Staff
Edith	Wagan		Population Health Research Institute	Hamilton, Ontario, Canada	Project Office Study Staff
Kayla	Pohl		Population Health Research Institute	Hamilton, Ontario, Canada	Project Office Study Staff
Sara	Chenier	BSc	Population Health Research Institute	Hamilton, Ontario, Canada	Project Office Study Staff
Steven	Chen		Population Health Research Institute	Hamilton, Ontario, Canada	Project Office Study Staff (Student Volunteer)
Riya	Srivastava		Population Health Research Institute	Hamilton, Ontario, Canada	Project Office Study Staff (Student)
Frances	Carandang		Population Health Research Institute	Hamilton, Ontario, Canada	Cardio-Oncology Fellowship
Enrique	Gonzalez		Population Health Research Institute	Hamilton, Ontario, Canada	Cardio-Oncology Fellowship
Harry	Klimis		Population Health Research Institute	Hamilton, Ontario, Canada	Cardio-Oncology Fellowship
Sophia	Laird		Population Health Research Institute	Hamilton, Ontario, Canada	Cardio-Oncology Fellowship
