# Supplementary material for: Specialist Referral for Cardiovascular Risk in Patients With Prostate Cancer: A Randomized Clinical Trial
Source: JAMA Intern Med. 2026 Aug 30:e264773. Online ahead of print. doi: 10.1001/jamainternmed.2026.4773 (PMC13527639; doi:10.1001/jamainternmed.2026.4773)
Supplement: Supplementary file 5 [file jamainternmed-e264773-s005.pdf]

Data Sharing Statement

Data
Additional Information: NCT03127631 www.clinicaltrials.gov
Data available: No

Additional Information
Explanation for why data not available: No individual participant data will be shared. The study protocol and statistical analysis plan are available in the Supplementary Material. Requests for access to study data cannot be accommodated because participant consent and institutional ethics approvals do not permit sharing of individual-level data outside the study team.
